# A telomere-to-telomere phased genome of an octoploid strawberry reveals a receptor kinase conferring anthracnose resistance

**DOI:** 10.1093/gigascience/giaf005

**Published:** 2025-03-12

**Authors:** Hyeondae Han, Natalia Salinas, Christopher R Barbey, Yoon Jeong Jang, Zhen Fan, Sujeet Verma, Vance M Whitaker, Seonghee Lee

**Affiliations:** Horticultural Sciences Department, University of Florida, IFAS Gulf Coast Research and Education Center, Wimauma, FL, 33598, USA; Vegetable Research Division, National Institute of Horticultural and Herbal Science, Rural Development Administration, Wanju 55365, Korea; Horticultural Sciences Department, University of Florida, IFAS Gulf Coast Research and Education Center, Wimauma, FL, 33598, USA; Horticultural Sciences Department, University of Florida, IFAS Gulf Coast Research and Education Center, Wimauma, FL, 33598, USA; Horticultural Sciences Department, University of Florida, IFAS Gulf Coast Research and Education Center, Wimauma, FL, 33598, USA; Vegetable Research Division, National Institute of Horticultural and Herbal Science, Rural Development Administration, Wanju 55365, Korea; Horticultural Sciences Department, University of Florida, IFAS Gulf Coast Research and Education Center, Wimauma, FL, 33598, USA; Horticultural Sciences Department, University of Florida, IFAS Gulf Coast Research and Education Center, Wimauma, FL, 33598, USA; Horticultural Sciences Department, University of Florida, IFAS Gulf Coast Research and Education Center, Wimauma, FL, 33598, USA; Horticultural Sciences Department, University of Florida, IFAS Gulf Coast Research and Education Center, Wimauma, FL, 33598, USA

**Keywords:** Anthracnose, fruit rot, root necrosis, *Colletotrichum acutatum*, *FaRCa1*

## Abstract

**Background:**

Cultivated strawberry (*Fragaria xananassa* Duch.), an allo-octoploid species arising from at least 3 diploid progenitors, poses a challenge for genomic analysis due to its high levels of heterozygosity and the complex nature of its polyploid genome.

**Results:**

This study developed the complete haplotype-phased genome sequence from a short-day strawberry, ‘Florida Brilliance’ without parental data, assembling 56 chromosomes from telomere to telomere. This assembly was achieved with high-fidelity long reads and high-throughput chromatic capture sequencing (Hi-C). The centromere core regions and 96,104 genes were annotated using long-read isoform RNA sequencing. Using the high quality of the haplotype-phased reference genome, FaFB1, we identified the causal mutation within the gene encoding *Leaf Rust 10 Disease-Resistance Locus Receptor-like Protein Kinase* (*LRK10*) that confers resistance to anthracnose fruit rot (AFR). This disease is caused by the *Colletotrichum acutatum* species complex and results in significant economic losses in strawberry production. Comparison of resistant and susceptible haplotype assemblies and full-length transcript data revealed a 29-bp insertion at the first exon of the susceptible allele, leading to a premature stop codon and loss of gene function. The functional role of *LRK10* in resistance to AFR was validated using a simplified *Agrobacterium*-based transformation method for transient gene expression analysis in strawberry fruits. Transient knockdown and overexpression of *LRK10* in fruit indicate a key role for *LRK10* in AFR resistance in strawberry.

**Conclusions:**

The FaFB1 assembly along with other resources will be valuable for the discovery of additional candidate genes associated with disease resistance and fruit quality, which will not only advance our understanding of genes and their functions but also facilitate advancements in genome editing in strawberry.

## Background

Cultivated strawberry (*Fragaria xananassa* Duch. NCBI:txid3747) is an allo-octoploid species (2n = 8x = 56) that originated from spontaneous interspecific hybrids of two wild octoploid species *Fragaria chiloensis* Duch. and *Fragaria virginiana* Duch [1]. Moreover, strawberry is the most widely distributed fruit crop in the world and serves as a model species for the *Rosaceae* family, which includes 27 fruit crops. Until recently, sequencing technologies were not sufficiently advanced to produce highly accurate assemblies of complex genomes. A complete and accurate sequence for both alleles at any given genomic region is a powerful resource for functional genomics research. The first chromosome-scale genome assembly for octoploid strawberry ‘Camarosa’ [2] contributed to an examination of the evolutionary history of the octoploid strawberry, its domestication, and studies of disease resistance and fruit quality [3–5]. It is proposed that the octoploid progenitors of cultivated strawberry emerged through consecutive stages of polyploidization of at least 3 diploid progenitor species over a million years ago, with the major diploid progenitors identified as *Fragaria vesca* and *Fragaria iinumae* [2, 6]. In addition, chromosome-scale genome assembly has shown that diploid subgenomes are not static subgenomes. Instead, the subgenomes dynamically evolved through homoeologous exchanges, commonly observed in neopolyploids. Because the cultivated strawberry chromosomes are a complex mix of genes from various phylogenetic backgrounds through homoeologous exchanges, Edger et al. [2] have introduced a nomenclature system (A–D) that avoids overly simplified direct assignments to any specific diploid progenitor. Hardigan et al. [4] recently proposed a subgenome nomenclature to assign the 28 homoeologous chromosomes to subgenomes A, B, C, and D based on similarity to *F. vesca, F. innumae*, and 2 remaining diploid ancestors. Because of its complex genome, functional genomics for octoploid strawberry have lagged compared to diploids. Yet the commercial importance of cultivated strawberry highlights a need for high-quality genomes to drive selective breeding and gene-editing applications.

The reference genome assembly is a fundamental platform to investigate the biological features of organisms. To produce a genome assembly, parental haplotypes are often collapsed to consensus sequences, referred to as a haplotype-merged assembly. The haplotype-merged assembly of a highly heterozygous and polyploidy genome means loss of genetic variation from 1 of the 2 haplotypes derived from both parents, resulting in a limited view of genetic diversity. In this context, the haplotype-phased genome assembly is of great importance to thoroughly understand the genetic variation that characterizes the genome. Recently, using high-fidelity (HiFi) long-read sequencing, the haplotype-phased genome of the day-neutral strawberry ‘Royal Royce’ was reported by applying the trio-binning pipeline with parental short reads [7]. In this study, the fully haplotype-phased genome from a short-day strawberry, ‘Florida Brilliance,’ was completed from telomere to telomere (T2T) without parental data by combining PacBio HiFi reads, Hi-C chromatin interaction data, and full-length transcriptome sequencing (Isoform sequencing) [8, 9]. Long-read sequencing technology combined with computational algorithms has contributed to completing not only the T2T human genome but also plant species such as corn [10], banana [11], diploid strawberry [12], and octoploid strawberry [13].

High-quality assemblies of highly heterozygous polyploid genomes are powerful for investing structural variations and identifying candidate genes associated with plant traits. The genome of ‘Florida Brilliance,’ FaFB1, has particular value as this cultivar is widely grown and used in breeding and thus possesses economically important gene-trait mechanisms. For instance, a major disease resistance locus, *FaRCa1*, was recently identified in strawberry that confers strong resistance to anthracnose fruit rot (AFR), explaining at least 50% of the phenotypic variation across trials, and moderate resistance to anthracnose root necrosis (ARN) caused by the *Colletotrichum acutatum* species complex [14, 15]. Previous European research demonstrated that a dominant allele at the single locus *Rca2* contributes resistance to *C. acutatum* isolate 688b from pathogenicity group (PG) 2 in strawberry [16]. *Rca2* is present in European cultivars Mamie, Gariguette, and Belrubi and in US cultivars Sequoia, Dover, and US15 [17]. *Rca2* is also present in 2 genotypes of *F. vesca* and one of *Fragaria moschata*, which have shown a resistance phenotype [18]. Resistance to *C. acutatum* has also been observed in *F. chiloensis* and *F. virginiana* [16, 19]. It was also reported that minor genes control intermediate levels of resistance in cultivar Addie [19]. Five other quantitative trait loci (QTLs) were found to give moderate resistance to the *C. acutatum* isolate 494a, which was assigned to PG-1, in a cross between ‘Capitola’ and CF111664 [20]. Disease response was recorded on vegetative tissue of the whole plant using the scale from 0 (no visual symptoms) to 5 (death plant). Since the *Rca2* resistance allele was reportedly present in the University of Florida (UF) germplasm, it was originally assumed that *Rca2* was the main resistance source in the UF breeding population. However, molecular markers linked to *Rca2*, which give resistance to *C. acutatum* isolate 688b and 1267b from PG-263, do not explain resistance/susceptibility to AFR in UF cultivars [20, 21]. UF cultivars possessing *FaRCa1* are a source for AFR resistance in other breeding programs worldwide.

One of the major challenges in strawberry genetics has been the availability of high-throughput, subgenome-specific markers. Since the cultivated strawberry has 4 subgenomes, each locus can represent up to 8 different alleles in a single individual, making it difficult to accurately analyze segregation. Recently, strawberry subgenome diversity was estimated based on whole-genome shotgun (WGS) sequencing of 93 genealogically and phylogenetically diverse *F. xananassa, F. chiloensis*, and *F. virginiana* individuals [22]. Subgenome-specific polymorphisms that were able to distinguish homologous from homoeologous DNA sequences on every chromosome were used to develop 850 K and 50 K single nucleotide polymorphism (SNP) arrays [22]. These arrays now provide an abundance of subgenome-specific SNPs for use in genetic studies in strawberries. A robust and easily scored DNA test is now available to breeders for selecting for resistance to both the fruit and root forms of strawberry anthracnose [15]. While this locus has been important in breeding, candidate genes for AFR resistance have not yet been characterized.

Plants use a diverse group of receptors located in the cell membrane that can perceive signals from the exterior, such as changes in temperature, light, nutrition, and presence of pathogens, and induce adequate responses to ensure plant survival. These receptors can be classified into 2 main groups: first, the nucleotide-binding site leucine-rich repeat (NBS-LRR) receptors and histidine kinase receptors and, second, the receptor protein kinases (RPKs). RPKs in plants are grouped into 2 main major subclasses based on their substrate specificity. The first subclass comprises serine/threonine kinases (STKs) that phosphorylate serine and threonine residues, and the second subclass consists of receptor histidine kinases (RHKs) that phosphorylate histidine residues [23]. Most RPKs in plants share a common structure that includes predicted extracellular signal sequence, an LRR, a single-pass transmembrane helix, and a cytoplasmic kinase domain with the serine/threonine consensus sequence. RLKs have been described as resistant genes that initiate disease response to specific pathogens, but RLKs also can play an important role in innate immunity [24]. A gene encoding Leaf Rust 10 Disease-Resistance Locus Receptor-like Protein Kinase (*LRK10*), identified as a candidate gene for AFR resistance in this study, is a member of the RLK family and most closely related to wheat LRK10 associated with disease resistance. In *Arabidopsis, LRK10* is involved in ABA-mediated signaling and drought resistance [25].

‘Florida Brilliance’ is the main short-day cultivar commercially grown in Florida and is heterozygous (*Ca1ca1*) at the *FaRCa1* locus and resistant to ARN and AFR. In this study, we describe a telomere-to-telomere haplotype-phased genome of ‘Florida Brilliance’ (FaFB1). A complete and accurate sequence for both alleles at any given genomic region is a powerful resource for functional genomics research. The highly accurate sequences of both alleles led to the discovery of a causal mutation and functional validation of the receptor kinase *LRK10* for AFR disease resistance in the *FaRCa1* region.

## Results and Discussion

### Telomere-to-telomere haplotype-phased assembly of the octoploid strawberry genome

In our preliminary experiments, we attempted a trio-binning approach using high-fidelity (HiFi) long reads from ‘Florida Brilliance’ and its parents FL 11.31–14 (female parent) and FL 10–153 (male parent) [26]. In the process, we discovered that the male parent was incorrectly identified and is unknown, which did not allow a trio-binning approach. As an alternative, HiFi long reads (Pacific Biosciences of California) and Hi-C chromatin interaction data (Dovetail) were used to assemble the short-day variety of ‘Florida Brilliance’ octoploid strawberry genome (Supplementary Table S1, Supplementary Fig. S1). Using 5 single-molecular real-time cells in the PacBio Sequel II platform, 144.1 Gb of sequences were generated in 9.1 M reads. The average read length was 15,834.8 bp. The Hi-C data were generated on Novaseq6000 and contained 86.5 Gb of sequences in 286 M paired-end reads with an average length of 151 bp. The combined power of these data produced a draft assembly with an N50 of 23.7 Mb for haplotype-1 (H-1) and 26.7 Mb for haplotype-2 (H-2), indicating that on average, 1 contig corresponded to a single chromosome. This compares to N50 for ‘Camarosa’ of 3.9 Mb and ‘Royal Royce’ of 11 Mb [7]. Before scaffolding, the BUSCO scores were 99.2% in the H-1 assembly and 99.1% in H-2 assembly. When comparing the full assembly with the HiFi reads of FaFB1 using Merqury, the results revealed a high base accuracy (quality value [QV] >69.8), indicating that 99.99% of HiFi reads were found in the combined assembly. The final assembly contained 99.1% complete gene models with a majority (96.6%) of the duplicated complete gene models in both H-1 and H-2 genome assemblies (Table 1). Using Merqury, we identified and organized phased blocks containing 2 or more markers of uniquely identified *k*-mers derived from the same genotype. The N50 of the phased blocks was 2.8 Mb for H-1 and 3.7 Mb for H-2, with the longest phased block sizes being 26.2 Mb for H-1 and 30.1 Mb for H-2, respectively. Although we do not have the full parental information for ‘Florida Brilliance,’ we do know the female parent, FL 11.31–14. Therefore, we evaluated the phasing quality using the whole-genome paired-end reads of FL 11.31–14. Due to the inherent ambiguity in Hi-C phasing, where paternal and maternal chromosomes cannot be distinguished in the offspring’s cells, *k*-mers from the female parent FL 11.31–14 were found in both H-1 and H-2 (Supplementary Fig. S2). As shown in Supplementary Fig. S2, the phased contig blocks for FL 11.31–14 were arbitrarily assigned to either H-1 or H-2 assemblies. Chromosome names in the ‘Florida Brilliance’ genome assemblies followed the nomenclature proposed by Hardigan et al. [7] and used in FaRR1, reflecting the proposed diploid origins of each subgenome (A, B, C, and D) [7].

**Table 1:** Statistics of the ‘Florida Brilliance’ genome assembly and annotation

Assembly metrics	Value	
	Haplotype-1	Haplotype-2
**Draft assembly**		
Number of contigs	3,716	1,226
Number of contigs (≥50 kb)	750	618
Length of largest contig (Mb)	34.6	34.8
Assembled genome size (Mb)	866	839.13
GC content (%)	40.67	39.84
Length of contig N50 (Mb)	23.72	26.65
Length of contig N75 (Mb)	12.38	17.30
L50	18	15
L75	31	24
BUSCO (%)	99.2	99.1
Single	2.6	2.5
Duplicated	96.6	96.6
Fragmented	0	0.1
Missing	0.8	0.8
Base accuracy (QV by Merqury)	69.9	68.5
**Final assembly**		
Assembled genome size (Mb)	784.9	781.0
Number of anchored contigs	137	86
BUSCO (%)	99.1	99.1
Single	2.5	2.3
Duplicated	96.6	96.8
Fragmented	0	0.1
Missing	0.9	0.8

Since the first report of a chromosome-scale reference genome [2], several octoploid strawberry (*F. xananassa*) reference genomes using HiFi reads have been generated [2, 7, 27–29]. The high accuracy of HiFi reads notably improved the assembly quality for octoploid strawberry. The N50 of ‘Florida Brilliance’ initial H-1 and H-2 assemblies were 23.7 Mb and 26.7 Mb and comparable to other genomes: Wongyo3115 (9.84 Mb), ‘Royal Royce’ (11 Mb) [7], ‘FL15.89-25’ (12.8 Mb) [30], ‘Yanli’(27.5 Mb) [29], *F. chiloensis* (10.8 Mb) [31], *F. chiloensis × F. virginiana* (11.3 and 8.9 Mb) [32], and ‘Reikou’ (3.9 Mb) [28]. The final assembly consisted of 784.9 Mb for H-1 and 781.0 Mb for H-2, which are similar to ‘Royal Royce’ (784 Mb) [7] and other genomes [27, 29, 30]. When comparing the corresponding chromosome lengths between ‘Florida Brilliance’ and ‘Royal Royce,’ we found high similarity between both varieties. (Supplementary Fig. S3). Overall, these results suggest that high-quality octoploid strawberry genomes can be successfully generated with and without parental data [9].

**Figure 1:**
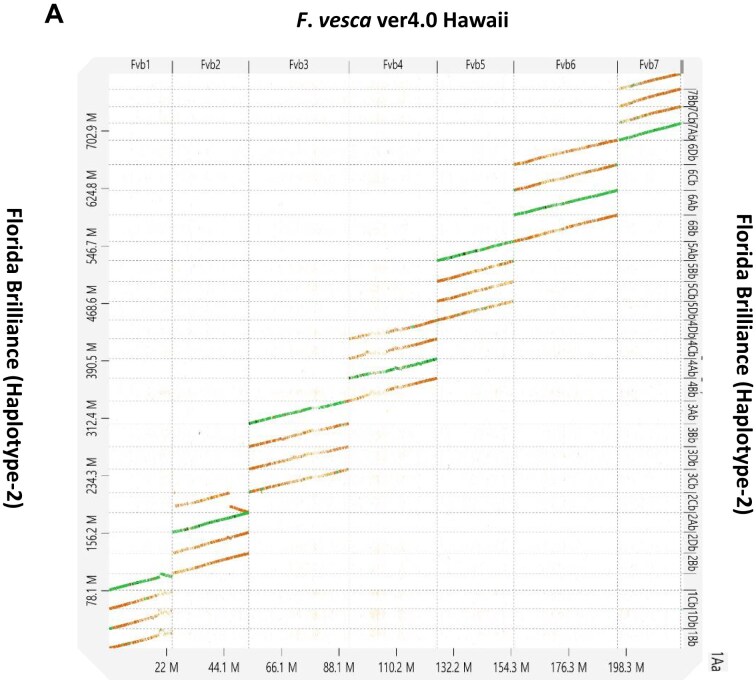
Dotplot of ‘Florida Brilliance’ H-2 assembly showing collinearity with diploid *F. vesca* version 4.0.

Hi-C reads were reanalyzed to complete scaffolding of the H-1 and H-2 assemblies. Among 286 M of Hi-C read pairs, approximately 92% of Hi-C reads were mapped to the assembly (Supplementary Table S2). The smudgeplot displays the genome structure of cultivated strawberry based on *k*-mer analysis, supporting allo-octoploid genome structure (Supplementary Fig. S4). Unique Hi-C read pairs were visualized via a Hi-C contact map of 28 chromosomes in each of the H-1 and H-2 assemblies (Supplementary Fig. S5). The Hi-C read pairs were evenly distributed, indicating a low probability of misassembly. There was high collinearity between the H-1 and H-2 assemblies of ‘Florida Brilliance’ (Supplementary Fig. S6). Alignment of the H-2 assembly against the diploid *F. vesca* v4.0 showed a high degree of collinearity except for major translocations on 1A and 2C (Fig. 1). As expected, ‘Florida Brilliance’ subgenome A showed clearly higher sequence similarity with *F. vesca* than the other 3 subgenomes. From each pair of pseudo-chromosomes (56 total) in the combined assembly, we selected the most continuous pseudomolecule to produce a final FaFB1 assembly. Genetic positions and physical coordinates of markers were collinear with a high degree of agreement between FaFB1 and the linkage map (Supplementary Fig. S7). Collinear gene pairs between ‘Florida Brilliance’ and *F. vesca* were identified (Supplementary Fig. S8) and were consistent with collinearity calculated by D-Genies (Supplementary Fig. S9).

**Figure 2:**
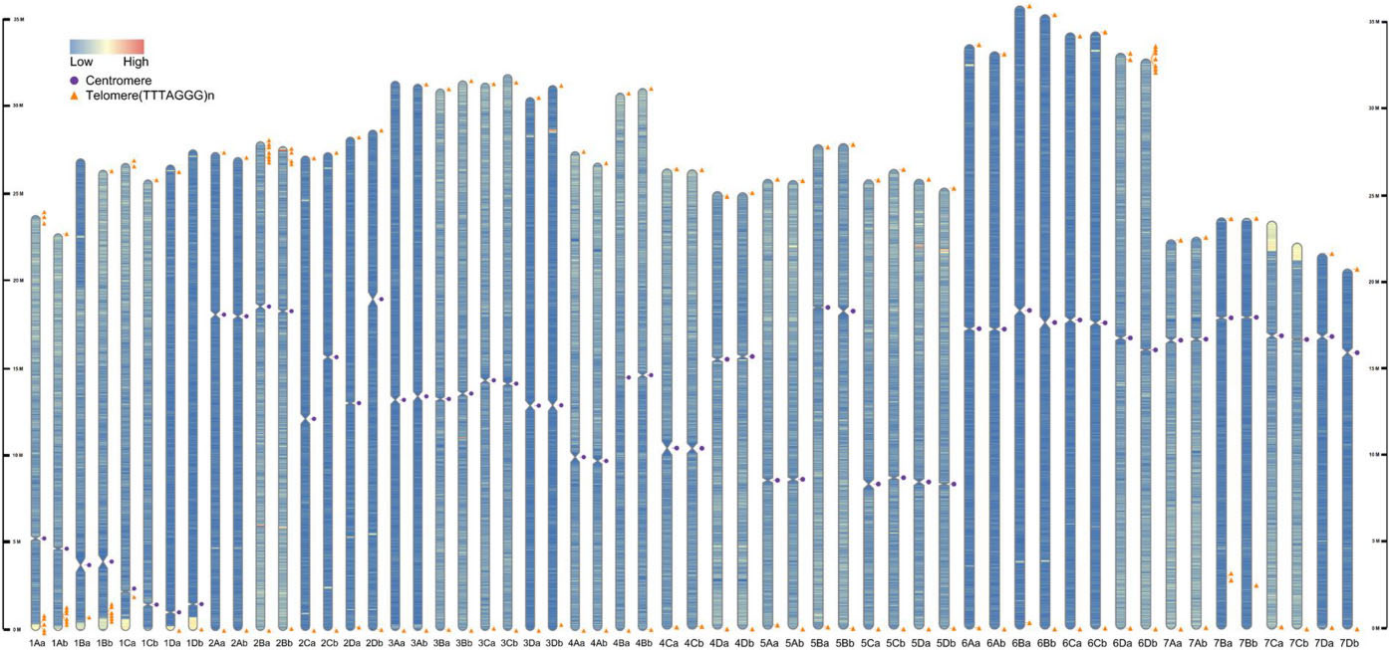
A telomere-to-telomere haplotype-phased genome assembly for octoploid strawberry ‘Florida Brilliance.’ Triangles (orange) represent regions with telomere repeats (5′-TTTAGGG-3′). Multiple triangles represent an interstitial telomere-like sequence. Circles (purple) indicate centromeres, with low gene density and high density of repetitive sequences, including LTR RTs and mini-satellites. Suffixes “a” and “b” were affixed to the chromosomes to denote each haplotype genome such as haplotype-1 or haplotype-2.

**Figure 3:**
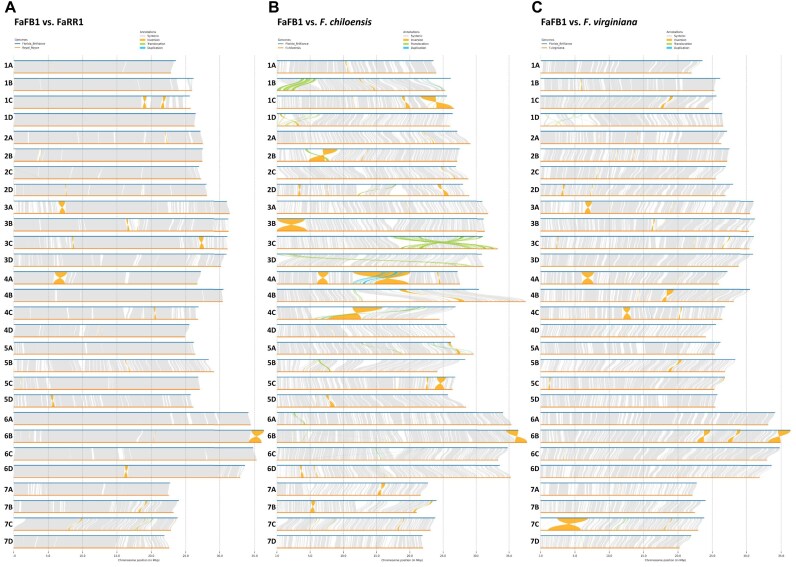
Collinearity analysis between the ‘Florida Brilliance’ (FaFB1) genome and other octoploid strawberry genomes. (A) Comparative genome analysis of FaFB1 and ‘Royal Royce’ (FaRR1), (B) FaFB1 vs. *F. chiloensis*, and (C) FaFB1 vs. *F. virginiana* [32].

Telomeres, the basic structure of eukaryotic chromosomes, are typically tandemly arranged mini-satellites following the formula (TxAyGz)n at the both ends of chromosomes [33]. The putative telomeric sequences (5′-TTTAGGG-3′) of octoploid strawberry were first described at both ends of 7 of the 28 pseudo-chromosomes of *F. chiloensis* [31]. Here, we identified 103 of a possible 112 telomeres across the 2 haplotypes. Putative telomeric sequences were found at or near the 5′ and/or 3′ ends in all 56 pseudo-chromosomes (Fig. 2 ). However, chromosomes 7Ba and 7Bb contained putative telomeric sequences located 6 Mb or 7 Mb from one end. In addition, 7 chromosomes (1Aa, 1Ab, 1Bb, 2Ba, 2Bb, 6Da, and 6Db) had short interstitial telomere-like sequences (for example, ∼10 repeats rather than ∼100), which could be due to misassemblies of these repetitive regions. FaFB1 demonstrated high collinearity with the published genome sequences of other octoploid strawberries: ‘Royal Royce’ (Fig. 3A), *F . chiloensis* (Fig. 3B), and *F . virginiana* (Fig. 3C). Gene distribution along the chromosomes followed the typical distribution of monocentric plant genomes, and the positions of centromeres were similar between the 2 haplotypes (Fig. 2). To evaluate both haplotype assemblies based on core eudicot genes, genome quality was assessed using the LTR Assembly Index (LAI) [34]. LAI for each chromosome ranged from 17.17 to 20.13. LAI for the combined genome was 19.72 (Supplementary Table S3), comparable to ‘Royal Royce’ and *F. vesca* Hawaii 4 [7]. In the combined assembly, there were 652 Mb of repetitive sequences accounting for 41.66% of the genome, which was similar to or higher than other published references, including ‘Royal Royce’ (38.4%), ‘Camarosa’ (36%) and ‘Wongyo 3115’ (38.75%) [27], ‘Benihoppe’ (43.3%), ‘Yanli’ (42.5%), and wild cotoploid strawberry (*F. chiloensis*; 44.3%). Most of these repeat sequences were composed by LTR class transposable elements (Supplementary Table S4). We applied the EDTA used in this study to the most recently reported Benihoppe, Yanli, and *F. chiloensis* to verify the frequency of repeated sequences with almost similar proportions. (Supplementary Table S4). Among them, 223,060 simple sequence repeats (SSRs) representing 0.16% of whole genome sequence were found (Supplementary Table S5).

### Annotation of gene content at the subgenome level of octoploid strawberry

**Figure 4:**
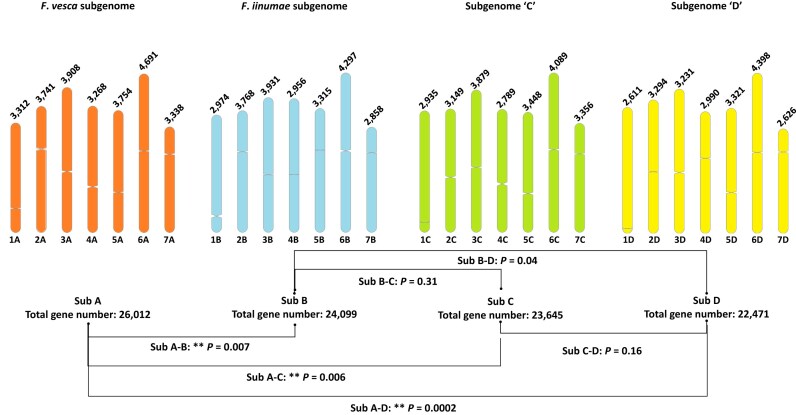
The number of genes predicted in subgenomes A, B, C, and D of the H-1 genome assembly for ‘Florida Brilliance.’ The number of genes is indicated above each chromosome. Subgenome A, *F. vesca*; subgenome B, *F. iinumae*; subgenome C and D, unknown.

To facilitate annotation, we generated a full-length transcriptome, including isoforms, of ‘Florida Brilliance’ using long-read Iso-seq for leaf, root, flower, green fruit, and red fruit samples. Additionally, Iso-seq data for 15 ‘Royal Royce’ tissues or treatments were included for gene prediction [7]. RNA-seq data representing *F. xananassa* tissues, including various tissues such as green achene, red achene, runner, and turning achene, were also downloaded from the NCBI sequence read archive. We obtained a set of 320,979 transcripts by aligning Iso-seq and RNA-seq datasets to the combined H-1 and H-2 assemblies. BUSCO analysis of the transcript assemblies revealed 2,209 complete core embryophyta genes (97.5%, 2.9% single-copy, 94.6% duplicated) with 1.1% fragmented and 1.4% missing core eudicot genes (Supplementary Table S6). Integrating *ab initio* predictions and evidence-based (RNA-seq and Iso-seq) prediction, 254,623 total gene models were predicted, roughly 127,000 models per haploid genome. After excluding transposable element (TE)–related gene models by 2 criteria, 202,456 genes remained in the final annotation with 100,850 models in the H-1 assembly, 101,606 models in the H-2 assembly, and 101,723 in the FaFB1 assembly (Supplementary Tables S7 and S8), similar to ‘Royal Royce’ (101,721) [7] and ‘Camarosa’ (108,087) [2] and quite different from ‘Wongyo3115’ (151,892) [27] and ‘Reikou’ (167,721) [28]. When classifying all predicted genes by subgenome, subgenome A, representing diploid progenitor *F. vesca*, accounted for 27.5% of genes, which is similar to ‘Royal Royce’ (27%) [7]. We observed biased gene distribution among the subgenomes of octoploid strawberry (A vs. B, *df* = 6, *P* = 0.007; A vs. C, *df* = 6, *P* = 0.006; A vs. D, *df* = 6, *P* = 0.0002), while the number of genes in subgenomes B, C, and D were not significantly different, except for the difference in the number of genes between subgenomes B and D (*P* = 0.04). This suggests a lower rate of gene loss in subgenome A (Fig. 4), which was also observed in the ‘Royal Royce’ genome (Supplementary  Tables S9 and S10).

### Haplotype alignment and candidate gene detection associated with the resistance locus *FaRCa1*

Previous studies have extensively documented the wide-ranging resistance variability to AFR within the UF breeding germplasm, with the majority of cultivars exhibiting moderate to high resistance levels [35–37]. Within the UF germplasm, resistance to AFR is primarily governed by a single locus, *FaRCa1* [14, 15]. This locus has been shown to account for a minimum of 50% of the phenotypic variance in AFR incidence across 2 distinct QTL discovery populations and has been corroborated in advanced selections and cultivars [14]. Utilizing 4 SNPs from the Axiom IStraw35 array [38], the *FaRCa1* region was delineated, with probe AX-89838986 demonstrating the highest explanatory power across both discovery and validation populations [14]. Subsequently, the 9 bp indel-based marker located in *FaRCa1* was developed for the marker-assisted selection of the trait [15]. So far, no additional loci were detected, indicating that *FaRCa1* singularly confers the observed resistance in the UF germplasm, as opposed to *Rca2*, which imparts resistance to certain strains of *C. acutatum* in Europe [16].

**Figure 5:**
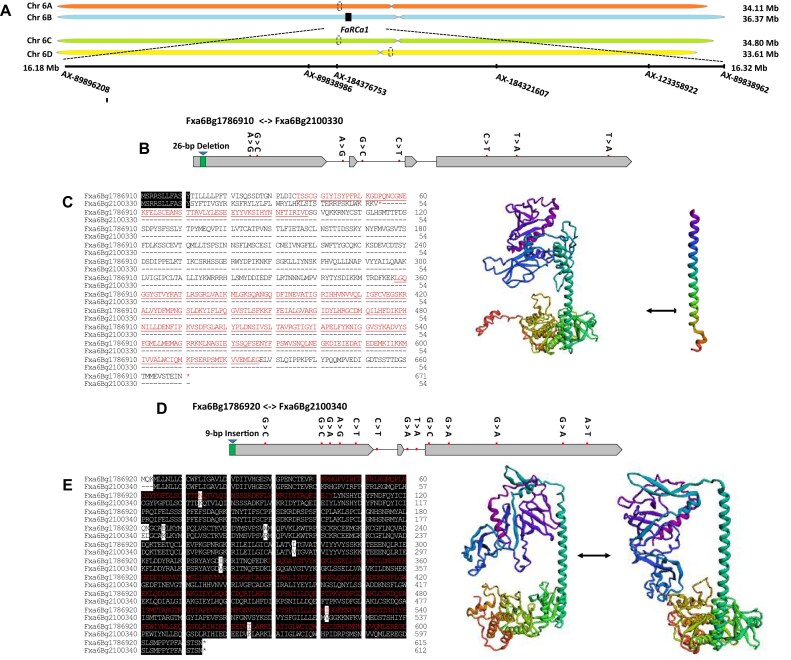
Identification of candidate genes, *LRK10A* (Fxa6Bg1786910) and *LRK10B* (Fxa6Bg1786920), in the *FaRCa1* region of ‘Florida Brilliance’ genome (FaFB1). (A) *FaRCa1* in Chr 6B of FaFB1 and positions of IStraw 35 Affymetrix Axiom and 50 K FanaSNP array SNPs. The 50 K FanaSNP Array probe “AX-89896208” and “AX-89838962” were located at 16.18 Mb and 16.32 Mb, respectively. (B, C) Gene structure, amino acid sequences, and 3-dimensional protein structure of resistant allele (*LRK10A*) and susceptible allele (Fxa6Bg2100330 named *LRK10a*). Gray rectangles and black lines represent exons and introns, respectively. The green box indicates insertions. Red lines represent the SNP position between the resistant allele in H-1 and the susceptible allele in H-2 genome of FaFB1. (D, E) Gene structure, amino acid sequences, and 3-dimensional protein structure of resistant allele (Fxa6Bg1786920 named *LRK10B*) and susceptible allele (Fxa6Bg2100340 named *LRK10b*). Gray rectangles and black lines represent exons and introns, respectively. The green box indicates deletion. Red lines represent the SNP position between the resistant allele in H-1 and susceptible allele in H-2 genome of FaFB1.

Resistant and susceptible haplotype assemblies for the previously described major resistance locus, *FaRCa1* (13, 14), were delimited between the 50 K FanaSNP Array probes “AX-89896208” and “AX-89838962” in chromosome 6B of the ‘Florida Brilliance’ genome (FaFB1) (Fig. 5A). The *FaRCa1* region was defined to 136 kb in the H-1 assembly (Resistant-*FaRCa1*; Chr. 6Ba) containing 13 genes and 158 kb in the H-2 assembly (Susceptible-*farca1*: Chr. 6Bb) containing 22 genes (Supplementary Fig. S10A and Supplementary Table S11). The resistant *FaRCa1* haplotype showed 6 locally collinear blocks (LCBs) when compared to the homoeologous *farca1* regions of chromosomes 6A and 6D (Supplementary Fig. S10A). In contrast, the homoeologous *farca1* haplotype on chromosomes 6A and 6D was 300 kb longer than the resistant haplotype and comprised more than 10 locally collinear blocks LCBs (Supplementary Fig. S10A). The susceptible haplotype of chromosome 6Bb showed 3 LCBs when aligned with the homologous genomic regions of ‘Royal Royce’ (Supplementary Fig. S10B), suggesting that this variety carries 2 susceptible alleles of *farca1*. This was expected as none of the previously assembled strawberry genomes was of a genotype known to carry this resistance locus.

The annotated genes located in the *RCa1* region were compared in the susceptible and resistant haplotype genome (Supplementary Fig. S11). We identified various types of SNPs and indels present in genes located at the *RCa1* region. The number of mutations ranged from 1 bp (Fxa6Bg1786950.m01 ↔ Fxa6Bg2100380.m01) to 60 bp (Fxa6Bg1787000.m01 ↔ Fxa6Bg2100480.m01). We examine sequence variations that could be associated with alterations in gene function and potentially linked to *RCa1*-mediated resistance, such as indels that are ≥2 bp in size, located in exons, causing frameshifts, and affecting gene functions involved in disease resistance. Only 2 genes, Fxa6Bg1786910 (named *LRK10A*) and Fxa6Bg1786920 (named *LRK10B*), were related to plant defense responses and functionally predicted as “*Leaf Rust 10 Disease-Resistance Locus Receptor-Like Protein Kinase*.” Additionally, it was found that the indel located in the promoter of *LRK10A* causes early stop codons and creates a premature termination codon. However, other sequence variations in different genes did not result in major frameshift changes. Instead, they resulted in only a few amino acid changes without stopping the gene from being fully transcribed in genes. *LRK10A* and *LRK10B* are closely located next each other and are 20 kb and 44.6 kb away from the 9 bp indel DNA marker that has been used for high-throughput marker-assisted seedling selection [15]. In a previous study, average AFR occurrence for individuals with the AA, AB, and BB genotypes using 9 bp indel DNA markers were 56.3%, 23.0%, and 17.7%, respectively. The full-length transcript data from ‘Florida Brilliance’ were used to confirm the sequence and structural variations of genes located in the *FaRCa1* region. Notable sequence variations were found at *LRK10A*. Sequence alignments between resistant *LRK10A* and susceptible *lrk10a* alleles showed a 26 bp deletion in the first coding region and 8 SNPs within the remaining coding sequence of the susceptible allele (Fig. 5B). This deletion introduces a premature stop codon that disrupts the downstream protein-coding sequence (Fig. 5C). The alignment of LRK10B between resistant and susceptible haplotypes revealed 99.3% (2082/2097) sequence similarity (Fig. 5E). The Iso-seq data of ‘Florida Brilliance’ showed that the full-length transcript of *LRK10A* is expressed in fruit tissue only, but *LRK10B* expression was not detected in any tissues of Iso-seq data. This finding suggests that *LRK10A* appears to have an important role in resistance to *C. acutatum* in strawberry. The wheat leaf rust kinase (*wlrk*) *RLK* gene family that encodes receptors involved in pathogen recognition was also constitutively expressed in the aerial parts, whereas no expression was detected in the roots [39].

LRK10A conforms to the typical features of RLK: a variable ectodomain for ligand binding, a single-pass transmembrane domain, and a cytoplasmic kinase domain (Fig. 5) [40]. Because the ability of plants’ receptor-like kinase to detect extracellular stimuli and transmit signals across the plasma membrane (PM) is vital for their defense against pathogens, LRK10A localized on the PM are expected to be responsible for detecting conserved microbe-associated molecular patterns (MAMPs) or damage-associated molecular patterns (DAMPs), initiating a cascade of immune responses [41]. The main biological and molecular functions of this protein are phosphorylation, protein kinase activity, and ATP and polysaccharide binding according to the InterPro 87.0 blast results. LRK10-like proteins belong to the receptor-like kinases (RLK) family, which is a subclass of the STKs, a major subclass of the RPKs [23, 24]. RPKs are receptors located in the cell membrane that can perceive changes from the plant exterior, including temperature, light, nutrition, and the invasion of pathogens.

### A receptor-like kinase, *LRK10*, contributes to anthracnose fruit rot resistance

LRK10A encodes a 670–amino acid protein, while the truncated susceptible allele consists of only 53 amino acids, resulting in the loss of the functional kinase domain (Fig. 5C). LRK10A is composed of 6 predicted regions: signal-peptide N-region (1–5 aa), signal peptide H-region (6–17 aa), signal peptide C-region (18–25 aa), noncytoplasmic domain (26–290 aa), transmembrane region (291–314 aa), and cytoplasmic domain (315–670 aa). The protein kinase domain can be found between 352 and 640 amino acids. Along the cytoplasmic domain, 2 sites can be distinguished: the protein kinase ATP binding site (358–380 aa) and the serine/threonine kinase active site (472–484 aa) according to the InterPro 87.0 database results. The main biological function of LRK10 is phosphorylation, and the main molecular functions are protein kinase activity and ATP and polysaccharide binding (InterPro 87.0). For LRK10B, there are only 3 differing amino acids in this gene between resistant and susceptible alleles, which do not alter the functional conserved domain (Fig. 5E). The expression of *LRK10A* and *LRK10B* in strawberry fruit showed significantly different expression patterns among the homozygous susceptible (*ca1ca1*), heterozygous (*Ca1ca1*), and homozygous resistant (*Ca1Ca1*) genotypes. Relative expression of LRK10A after *C. acutatum* inoculation decreased through time in the homozygous susceptible (*ca1ca1*) genotype, 16.74–68. Relative expression of LRK10A in homozygous resistant *Ca1Ca1* and heterozygous resistant *Ca1ca1* genotype increased after *C. acutatum* inoculation and remained at high levels at 24, 48, 72, and 96 hours postinoculation (hpi) (Supplementary Fig. S12). However, relative expression of gene *LRK10B* after *C. acutatum* inoculation was reduced over time in the susceptible genotype *ca1ca1* and the homozygous resistant genotype (*Ca1Ca1*). For the heterozygous resistant (*Ca1ca1*) ‘Florida Brilliance,’ the relative expression of gene *LRK10B* seemed relatively similar at 24, 48, 72, and 96 hpi. This finding indicates the involvement of *LRK10A* in the resistance against the pathogen.

**Figure 6:**
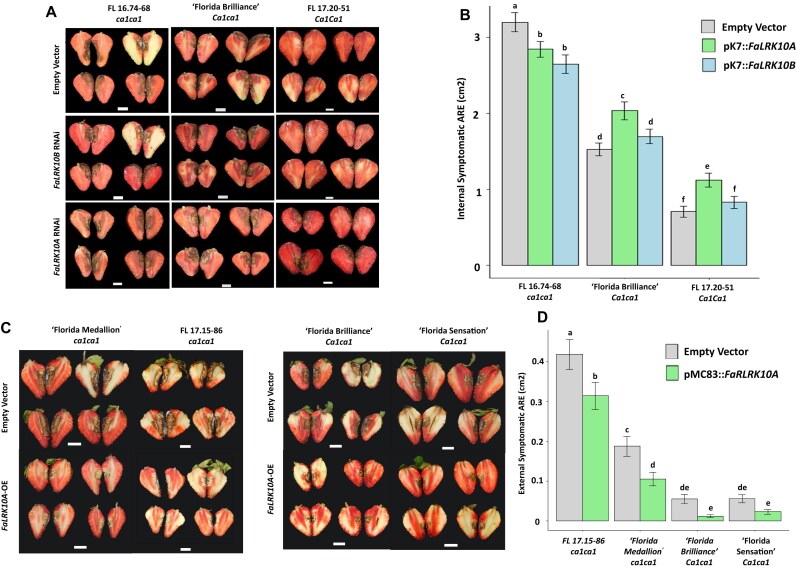
Internal symptoms of anthracnose fruit rot in fruit transient knockdown and transient overexpression. (A) Phenotype of transient knockdown of *LRK10A* and *LRK10B* in strawberry fruit from 3 genotypes: FL 16.74–68 (*ca1ca1*), ‘Florida Brilliance’ (*Ca1ca1*), and FL 17.20–51 (*Ca1Ca1*). *Agrobacterium*-infiltrated fruits with three treatments: Empty Vector, pK7::*LRK10A* RNAi-mediated knockdown, and pK7::*LRK10B* RNAi-mediated knockdown. (B) Internal symptomatic area (cm^2^) in fruits from genotypes and treatments described in (A). (C) Phenotype of transient overexpression of *LRK10A* in strawberry fruit from 4 genotypes: ‘Florida Medallion’ (*ca1ca1*), FL 17.15–86 (*ca1ca1*), ‘Florida Brilliance’ (*Ca1ca1*), and ‘Florida Sensation’ (*Ca1Ca1*). *Agrobacterium*-infiltrated fruits with two treatments: Empty Vector, pMDC83::*LRK10A*. (D) Internal symptomatic area (cm^2^) in fruits from genotypes and treatments described in (C). Error bars represent SE based on 3 biological replicates. The white line represents a scale of 1 cm. Lowercase letters indicate significantly different means using the least significant difference test (α = 0.05). Bars indicate standard errors.

To further validate the functional role of these 2 genes against the *C. acutatum* species complex, we applied robust and efficient *Agrobacterium*-based transformation methods for transient gene expression analysis in fruits of strawberry. RNA interference (RNAi) constructs targeting both *LRK10A* and *LRK10B* were designed (Supplementary Table S12). *Agrobacterium tumefaciens* EH105 containing RNAi constructs targeting each gene, *LRK10A*: RNAi and *LRK10B*: RNAi, was injected in green fruits of homozygous resistant (*Ca1Ca1*) 17.20–51, heterozygous resistant (*Ca1ca1*) ‘Florida Brilliance,’ and homozygous susceptible (*ca1ca1*) 16.74–68. *A. tumefaciens* containing empty vectors was also used as control in each genotype. The mRNA level was reduced by a maximum of approximately 35% in *LRK10A*- and *LRK10B*-knockdown individuals compared to the empty vector control (Supplementary Fig. S13A). The internal symptom area of the *LRK10A-*knockdown individuals increased significantly in resistant genotypes (homozygous—*Ca1Ca1* and heterozygous—*Ca1ca1*) at 16 days after inoculation (DAI) (Fig. 6A, B). In contrast, *LRK10B*-knockdown individuals showed no significant difference in internal symptom area (Supplementary Fig. S13B). In the transient overexpression assay, the internal symptom area of all genotypes in *LRK10A-*overexpressed fruit was significantly reduced compared to the empty vector control fruit (Fig. 6C, D). The expression level of *LRK10A* in the overexpression fruit was 3 to 6 times higher than that in the empty vector control, respectively (Supplementary Fig. S13B). These findings indicate that *LRK10A* is a key genetic factor regulating the *FaRCa1*-mediated resistance to *C. acutatum*.

## Conclusion

In conclusion, we have described a high-quality T2T, haplotype-phased genome of octoploid strawberry assembled without parental information. The accuracy and completeness of this reference genome allow for the precise identification of heterozygous alleles associated with specific traits. Leveraging this haplotype-phased genome alongside comprehensive full-length transcriptome data led to the identification of the candidate gene, *LRK10A*, by comparing the genomic and transcriptomic sequences of resistant and susceptible alleles in the heterozygous ‘Florida Brilliance.’ The *Agrobacterium*-mediated transient knockdown and overexpression experiments in strawberry fruit provided consistent functional evidence for *LRK10A* in *FaRCa1*-mediated resistance against the *C. acutatum* complex in strawberry. This resource, along with other complete strawberry genomes, will continue to be a powerful tool for discovering gene-to-trait mechanisms in cultivated strawberry.

## Methods

### Plant materials and long-read sequencing

Etiolated leaf tissues of ‘Florida Brilliance’ were used for genomic DNA extraction and library preparation at DNALink. The single-molecule real-time sequencing (SMRT) bell library was constructed using a PacBio DNA Template Prep Kit 1.0 (Pacific Biosciences). Quality and quantity of each library were checked using a 2100 Bioanalyzer (Agilent Technologies). The SMRT Bell-Polymerase complex was constructed using a PacBio Binding Kit 2.0 (Pacific Biosciences) based on the manufacturer’s instructions. The complex was loaded onto 5 SMRT cells (Pacific Biosciences, Sequel SMRT Cell 1 M v2) and sequenced using a Sequel Sequencing Kit 2.1 (Pacific Biosciences, Sequel SMRT Cell 1 M v2). For each SMRT cell, 1 × 600-minute movies were captured using the Sequel sequencing platform (Pacific Biosciences) at DNALink. The quality of HiFi data were measured with LongQC [42].

For transcriptome sequencing, 6 ‘Florida Brilliance’ tissues were used for construction of PacBio Iso-seq libraries (Supplementary Table S1). About 10 g of 6 tissues was collected from 5 plants: flower, green fruit, red fruit, crown, young leaf, and root. All tissues were flash frozen at −80°C after washing with water. Total RNA was extracted using Spectrum Plant Total RNA Kit (Sigma-Aldrich) according to the manufacturer’s instructions. The extract was treated with DNAse I (Invitrogen) and resuspended in a total volume of 50 μL RNase-free water. The RNA samples were submitted to DNALink for preparation of the Iso-seq library and sequencing.

Among the Iso-seq sequences, when restricted to HiFi (QV ≥20), 45,577 to 56,707 reads with an average length of about 2,500 bp were obtained from 6 tissues. In total, 12.1 Gb of ‘Royal Royce’ Iso-seq sequence data [7] and 511 Gb of short-read Read-seq were used as input for transcriptome assembly and gene annotation (Supplementary Table S13).

### *De novo* genome assembly and validation

The genome characteristics, including genome size and repetitive elements, were estimated using PacBio HiFi data by *k*-mer spectrum distribution analysis for *k* = 21 in KMC3 [43] and GENOMESCOPE v 2.0 (RRID:SCR_017014) [44]. The HiFi and Hi-C reads were used to produce a haplotype-phased assembly without sequencing of parents using Hifiasm (RRID:SCR_021069) [9]. Hifiasm was run with the following command according to the developer’s recommendation for heterozygous crops: hifiasm -o <outputPrefix> -t <nThreads> -D10 <Hifi-reads.fasta> –h1 <Hi-C_reads1> –h2 <Hi-C_reads2>. SALSA2, which is an Hi-C–based scaffolding program, was used for scaffolding contigs. Hi-C reads were mapped via the Arima-HiC mapping pipeline. After mapping Hi-C reads to each phased genome assembly using BWA v0.7.17 [45], the sequence alignment map (SAM) format was converted to bed format using SAMtools (RRID:SCR_002105) [46] and BEDTools (RRID:SCR_006646) [47] before SALSA2 (RRID:SCR_022013) scaffolding [48], which was run with parameters -e GATC -m yes. Hi-C reads were mapped to chromosomes using HiC-Pro (RRID:SCR_017643) [49] in order to assess the quality of the assembly. The interaction matrix of whole chromosomes was visualized with heatmaps. Remaining contigs were scaffolded and oriented based on the ‘15.89–25’ reference genome using Ragtag [50]. A high-density genetic map was developed using a total of 169 F1 individuals from a cross between ‘Florida Brilliance’ and ‘FL 16.33-8’. Axiom 50 K FanaSNP Genotyping Array was used to genotype all 169 F1 individuals. Markers were filtered to have <5% missing data and fit segregation ratios of 1:1 and 1:2:1 (α = 0.05). Marker genotype calls were recoded to fit the Joinmap 4.1 linkage mapping requirement. For example, markers with paternal segregation (AA × AB or BB × AB) were coded as “nn × np,” markers with maternal segregation (AB × AA or AB × BB) were coded as “lm × ll,” and markers segregating in both parents (AB × AB) were coded as “hk × hk.” Mapping was conducted in an iterative process using the maximum likelihood algorithm in JoinMap 4.1 with default settings. After each round of mapping, a graphical genotyping approach was applied to identify singletons to fix the marker order and regions with low marker density or gaps caused by segregation distortion. The genetic linkage map of ‘Florida Brilliance’ consisting of 10,269 SNP markers was used to validate the scaffolds from the FaFB1 whole-genome assembly. SNP probe sequences used in the construction of linkage maps were mapped to the FaFB1 assembly sequence using the blastn (RRID:SCR_001598) procedure [51]. Alignments were filtered to retain markers if they matched the unique sequence position in the FaFB1 phased genome assembly and with a maximum of 2 mismatches in the second best hit. The alignments were queried to detect problematic scaffolds mapped with SNP probes from different linkage groups (LGs). The number of scaffolds with SNP probes mapped from different LGs was used as a metric in the quality assessment of FaFB1 assembly.

### Validation of assembly quality

Genome assembly statistics were calculated using QUAST (RRID:SCR_001228) version 5.0.266. Merqury (RRID:SCR_022964) version 1.3 was used to measure assembly consensus QV and evaluate assembly based on efficient *k*-mer set operations [52]. The completeness of the haploid assemblies and protein-coding gene annotations was assessed with the BUSCO (RRID:SCR_015008) database [53]. The scaffolds were inspected on the Hi-C contact map. Hi-C reads were trimmed with Homer (RRID:SCR_010881) [54] and mapped to both haploid assemblies using HiC-Pro version 3.0 [49] and visualized in Juicebox (RRID:SCR_021172) version 1.11 [55]. LAI [34] for each subgenome was calculated using LTR-retriever [56] along with whole-genome TE annotations and intact LTR retrotransposons identified by EDTA [57].

Proteins of diploid strawberry *F. vesca* were collected for all-against-all alignments to predicted proteins for octoploid strawberry ‘Florida Brilliance.’ These alignments were passed to MCScanX (RRID:SCR_022067) to identify synteny blocks [58]. DNA-level synteny between *F. vesca, F. xananassa, F. chiloensis*, and 2 phased genome assemblies of ‘Florida Brilliance’ was plotted using D-GENIES (RRID:SCR_018967) [59] with default parameters after aligning with minimap2.

### Genome annotation

TEs were annotated using EDTA (RRID:SCR_022063) v1.9.6 with default parameters [60]. The TE annotation library was generated by EDTA in a separate run. TE regions of both haploid assemblies were masked by RepeatMasker v4.1.1 provided with the repeat library. SSRs or microsatellites were mined using SSR Finder [61] on Genome Sequence Annotation Server v6.0 (GenSAS). Telomeric repeats were annotated using BIOSERF [62].

To increase the accuracy of gene annotation, we generated a transcriptome assembly containing transcripts from ‘Florida Brilliance’ and *F. xananassa* expression data publicly available. Sixty-seven octoploid strawberry RNA-seq libraries were downloaded from the NCBI sequence read archive (SRA) (Supplementary File 1). The ‘Royal Royce’ Iso-seq reads were trimmed using Trimmomatic version 0.39 and mapped to 2 phased assemblies using HISAT (RRID:SCR_015530) v2.2.1 [63] with default parameters. The ‘Florida Brilliance’ Iso-seq reads were aligned to the assemblies using minimap (RRID:SCR_018550) v2.2.1 [64]. Read alignments were converted to binary alignment map (BAM) format with samtools. Reference-guided transcriptome assembly was performed using StringTie (RRID:SCR_016323) v2.1.4 [65] with the Iso-seq and RNA-seq alignments as input. StringTie2 was run with default parameters for RNA-seq alignment and with the addition of long-read (-L) mode for Iso-seq alignments. Mikado (RRID:SCR_016159) v2 [66] was used to generate a nonredundant set of transcript assemblies with best-scoring transcript evidence at each locus. Match scores were measured for all transcriptome assemblies against the UniProt (RRID:SCR_002380) protein database using BLASTX (RRID:SCR_001653). TransDecoder (RRID:SCR_017647) v5.5.0 was used to predict the best 6-frame translations of the transcriptome assemblies from StringTie2, and then splice junctions for all merged RNA alignments were predicted with Portcullis (RRID:SCR_016442) v1.2.2 [67]. The Mikado scoring for any transcript assemblies derived from Iso-seq alignments was modified over RNA-seq alignments. TransDecoder was used to filter nonredundant, polished transcripts generated by Mikado to obtain best ORF scores. The ‘Florida Brilliance’ genome assembly was annotated using GenSAS v6.0 [61]. The Iso-seq and RNA-seq alignments were used to predict gene models using braker2. Functions of predicted gene models were annotated based on alignment using BlastP (RRID:SCR_001010) v2.2.28 to the UniProtKB (RRID:SCR_004426) database [68].

### Construction of RNAi and overexpression vectors

Hairpin structures were designed for *LRK10A* and *LRK10B*. Hairpin inserts were composed of a 300 bp stem, 100 bp loop, and *aatt*B sites for Gateway cloning (Supplementary Table S10). The 300 bp stem in each of the inserts was complementary to the first coding DNA sequence (CDS) region of *LRK10A* and *LRK10B*. The 758 bp fragments were synthesized using GeneArt gene synthesis (Thermo Fisher Scientific). The inserts were individually ligated upstream of the *Escherichia coli* sites to the vector pMK-QR containing the kanamycin selection gene. Vector pMK-QR containing the hairpin fragments was used in the Gateway protocol for *LRK10B* and *LRK10A* independently. Hairpins were cloned into the Gateway pDONR/Zeo vector (Thermo Fisher Scientific) using standard procedures. After checking insert identity, the fragments were inserted into the RNAi Gateway vector pK7GWIWG2(I). Vectors containing the constructs and empty vector were separately inserted into *A. tumefaciens* strain EHA105 by using an adapted freeze–thaw method [69]. The transformed cells were tested using PCR for the presence or absence of RNAi constructs (Supplementary Table S3). For overexpression studies, the coding region of gene (CDS) *LRK10A* was obtained based on the ‘Florida Brilliance’ genome annotation, and the corresponding CDS sequence was synthesized by Novogene. The *LRK10A* synthesized gene fragment was cloned into the Gateway pDONR/Zeo entry vector (Thermo Fisher Scientific) following the manufacturer’s protocol. The pDONR/Zeo:: *LRK10A* entry vector was recombined into an overexpression vector as pMDC83 containing 2 × CaMV35s promoter through the LR reaction to generate an overexpression clone as pMDC83:: *LRK10A*.

### Fruit transient assay

*Agrobacterium*-mediated fruit transformation was performed as described by Pi et al. [70] and Zhao et al. [71] with modifications. Three varieties, 17.20–51 (*Ca1Ca1*), ‘Florida Brilliance’ (*Ca1ca1*), and 16.74–68 (*ca1ca1*), were used for the *Agrobacterium*-mediated fruit transient assay. “Three varieties, 17.20-51 (*Ca1Ca1*), ‘Florida Brilliance’ (*Ca1ca1*), and 16.74-68 (*ca1ca1*), are used for the *Agrobacterium*-mediated fruit transient knockdown assay. Four varieties, ‘Florida Medallion’ (*ca1ca1*), 17.15-86 (*ca1ca1*), ‘Florida Brilliance’ (*Ca1ca1*), and ‘Florida Sensation’ (*Ca1ca1*) were used for *Agrobacterium*-mediated fruit overexpression assay.”The *A. tumefaciens* EHA105 strain containing RNAi and overexpression constructs were revived by transferring cells from the 1:1 water/50% glycerol solution with sterilized toothpicks in 5 mL Luria Broth (LB) medium with rifampicin (15 mg/L) and spectinomycin (100 mg/L) 2 days before fruit agroinfiltration. Revived *A. tumefaciens* was transferred to 5 mL LB medium and was allowed to grow for 24 hours at 28°C and 230 rpm. Concentrations of bacterial cells were measured with OD_600_ using a Biomate 3S UV-visible spectrophotometer (Thermo Scientific). When cultures reached OD_600_ of ∼1, 1.25 mL was transferred to 250 mL LB in a flask. *A. tumefaciens* grew for about 18 hours at 28°C, 230 rpm until reaching an OD_600_ value of ∼1. Bacterial cells were collected by centrifuging the culture in 50-mL tubes at 4,000 rpm for 20 minutes. *Agrobacterium* cells were resuspended in 600 mL of activation buffer (4.43 g/L Murashige and Skoog medium, 10 mM MgCl_2_, 200 µM acetosyringone, 10 mM 2-(N-morpholino) ethane sulfonic acid [MES], pH 5.8). Concentration was fixed at OD_600_ = 0.5 for replication 1 and 0.8 for replications 2, 3, 4, and 5. The activated solution was kept in an orbital shaker for 3 hours at room temperature at 100 rpm.

Ninety green fruits of each genotype were harvested in the early morning. Healthy, uniform, white fruits at a similar development stage were preferred [71]. Pedicels were carefully removed to avoid contamination during the experiment. Fruit was disinfested by immersion in a 0.7% solution of sodium hypochlorite (bleach) for 6 minutes, rinsed with sterilized water, and placed in the hood for 20 minutes to dry [72]. Agroinfiltration was performed by injecting the activated solution for each of the constructs into the green fruit using 5-mL syringes until saturation [71]. Sterilized water was poured into the plastic containers to maintain humidity. Agroinfiltrated fruit was kept under 16 hours of daylight and 8 hours of dark conditions at room temperature.

Agroinfiltrated fruit was inoculated with the *C. acutatum* species complex 5 days after agroinfiltration. Inoculum was produced by growing the 3 isolates of *C. acutatum* (*C. nymphaeae*) 02–163, 02–179, and 03–32 separately on potato-dextrose agar at room temperature with constant light for 7 days until sporulation. Plates were flooded with sterile-distilled water when growth turned orange and reached near the border of the plate. A spore suspension for each isolate was passed through cheesecloth to remove dislodged mycelia. A final suspension was adjusted to 1 × 10^6^ conidia/mL by combining equal concentrations of each isolate. Fruits were inoculated with a single 20-µL drop of *C. acutatum* inoculum in the hood [73]. External and internal symptoms were analyzed using ImageJ 1.53e (National Institutes of Health) software. Area of fruit, area of external symptoms, area of halves of cut fruit, and area of internal symptom were obtained. Agroinfiltration, inoculation, and phenotype scoring were performed 5 times. Means were compared and separated using a 2-way analysis of variance and the least significant difference test in R software (R Core Team 2000).

### RNA extraction and quantitative reverse transcription PCR analysis

RNA was extracted by using the Spectrum Plant Total RNA Kit (Sigma-Aldrich) as recommended by the manufacturer. RNA concentration and quality were assessed using a Nanodrop 8000 Spectrophotometer (Thermo Scientific) and Qubit RNA broad range (BR) assay kits. cDNA for the knockdown and gene expression samples was synthesized in a 20-µL reaction composed of 4 µL LunaScript RT SuperMix Kit (New England Biolabs) and 16 µL containing 500 ng total RNA. The quantitative reverse transcription PCR (qRT-PCR) experiment was performed in triplicates of 5-µL reactions containing 2.5 µL of 2 × EvaGreen-based Forget-Me-Not qPCR (Biotium) master mix, 0.4 µL of 0.5 µM forward primer solution, 0.4 µL of 0.5 µM reverse primer solution, 1 µL diluted cDNA, and 0.7 µL DI H_2_O. The cDNA dilution at 5 ng/µL was used for the PCR reactions containing primers for the housekeeping gene *FaGapDH2* and the cDNA dilution at 20 ng/µL for PCR reactions with primers for genes *LRK10B* and *LRK10A* (Supplementary Table S14). The reactions were carried out in a 384-well PCR plate in a LightCycler 480 II Instrument (Roche) for both PCR and high resolution melting (HRM). Conditions of PCR were as follows: preincubation at 95°C for 5 minutes, initial denaturation at 95°C for 20 seconds, 40 cycles of denaturation at 95°C for 20 seconds, annealing at 62°C for 10 seconds, and extension at 72°C for 10 seconds. HRM conditions were 95°C for 5 seconds, 65°C for 1 minute, and 97°C continuous. Finally, the PCR product was cooled to 40°C for 30 seconds to allow heteroduplex formation. The raw cycle threshold values (Cp) from qRT-PCR runs were used to calculate relative expression using the ΔCt method.

### Gene expression profiling after *C. acutatum* inoculation

Forty-five white or white fruit with slight pink hue from homozygous resistant selection (*Ca1Ca1*) 17.20–51, heterozygous resistant (*Ca1ca1*) ‘Florida Brilliance,’ and homozygous susceptible selection (*ca1ca1*) 16.74–68 were harvested in the early morning. Healthy uniform fruit at similar development stages was preferred. Fruit disinfestation, classification, arrangement, and *C. acutatum* inoculation were done as previously described in the knockdown and overexpression experiments. Three biological replications composed of tissue from 3 fruits each were taken every day at the same time at 5 time points: 0, 24, 48, 72, and 96 hpi for each genotype. Samples collected at 0 hpi were taken from noninoculated fruit. Samples collected at 24, 48, 72, and 96 hpi were obtained by measuring 2-cm squares around the lesion caused by the *C. acutatum* inoculation. Fruit tissue was cut into small pieces and immediately frozen using liquid nitrogen. Samples were kept at −80°C.

## Supplementary Material

giaf005_Supplemental_Files

giaf005_GIGA-D-24-00173_Original_Submission

giaf005_GIGA-D-24-00173_Revision_1

giaf005_GIGA-D-24-00173_Revision_2

giaf005_Response_to_Reviewer_Comments_Original_Submission

giaf005_Response_to_Reviewer_Comments_Revision_1

giaf005_Reviewer_1_Report_Original_Submission Houcheng Zhou -- 6/27/2024

giaf005_Reviewer_2_Report_Original_Submission Thomas Medford Davis, Ph.D. -- 7/1/2024

giaf005_Reviewer_2_Report_Revision_1 Thomas Medford Davis, Ph.D. -- 10/22/2024

giaf005_Reviewer_3_Report_Original_Submission Fei Chen -- 7/7/2024

## Data Availability

High-throughput sequencing data analyzed in the present study are available under NCBI BioProject PRJNA888562. The Hi-C data are available through the NCBI SRA database (SRR21850459), and PacBio long-read sequencing data have been deposited in the NCBI SRA (Accession: SRR21850458). The Iso-Seq data are available through NCBI SRA: crown (SRR21895706), flower (SRR21895705), green fruit (SRR21895704), red fruit (SRR21895703), leaf (SRR21895702), and root (SRR21895701). The chromosome-level genome assembly and annotation files are available via Genome Database for Rosaceae [74]. All supporting data are available via the *GigaScience* repository, GigaDB [75].

## Additional Files

**Supplementary Fig. S1**. Haplotype-phased assembly of an octoploid strawberry genome using HiFi and Hi-C data.

**Supplementary Fig. S2**. Haplotype phasing assessment of ‘Florida Brilliance’ genomes with hapmers.

**Supplementary Fig. S3**. Chromosome length of ‘Florida Brilliance,’ ‘Royal Royce,’ and ‘Camarosa’ assemblies. ‘Florida Brilliance’ and ‘Royal Royce’ consist of 2 phased assemblies (H-1 and H-2).

**Supplementary Fig. S4**. Smudgeplot for ‘Florida Brilliance.’ The brightness of each smudge was determined by the number of *k*-mer pairs. The coloration indicates the approximate number of *k*-mer pairs per bin.

**Supplementary Fig. S5**. Hi-C contact map of H-1 (A) and H-2 (B) genome assemblies of ‘Florida Brilliance.’ Each red pixel represents a Hi-C pair. The dominant visual feature in every Hi-C heatmap is the strong diagonal representing the Hi-C pairs of loci uniquely anchored in each haplotype-phased assembly.

**Supplementary Fig. S6**. Dotplot of ‘Florida Brilliance’ H-1 and H-2 assemblies showing their collinearity.

**Supplementary Fig. S7**. Genetic positions of 10,269 markers from the linkage map for ‘Florida Brilliance’ against the corresponding physical positions in the ‘Florida Brilliance’ genome assembly.

**Supplementary Fig. S8**. Dotplot of collinear gene pairs between ‘Florida Brilliance’ and *F. vesca*. Homologous genes of H-1 (A) and H-2 (B) assemblies were compared against diploid *F. vesca*. Relative expression-level analysis of selected genes by qRT-PCR in different time stages of inoculated strawberry against *C. acutatum*.

**Supplementary Fig. S9**. Dotplot of ‘Florida Brilliance’ H-2 assembly to *F. xananassa* cv. Royal Royce (A), *F*. c*hiloensis* (B), and *F. virginiana* (C). Dot plots are produced using the DGENIE software and alignments with minimap2.

**Supplementary Fig. S10**. Comparative genomics analysis using Mauve alignment in Geneious Prime 2022.0.1 with the *FaRCa1* in Chr 6B from ‘Florida Brilliance’ (A) and regions homologous to *FaRCa1* in subgenomes Chr 6B from published genomes ‘Royal Royce,’ FL 15.89–25 and ‘Camarosa’ (B). Resistant alignments showed 3 locally collinear blocks (LCBs) when aligned with homologous *FaRCa1* regions in Chr 6B from ‘Royal Royce,’ FL15.89–25 and ‘Camarosa.’ *FaRCa1* in Chr 6B from ‘Florida Brilliance’ H-1 assembly showed different length from others.

**Supplementary Fig. S11**. Sequence alignment of genes within *FaRCa1* region. Thirteen genes from resistant haplotype *FaRCa1* were compared with counterparts from the susceptible haplotype. Each variant, including SNP and insertion/deletion, was visualized as an upper bar in sequence alignment.

**Supplementary Fig. S12**. Relative gene expression for *LRK10A* (A) and *LRK10B* (B) after *C. acutatum* inoculation for genotypes *ca1ca1, Ca1ca1*, and *Ca1Ca1*. Each bar depicts the log_2_-transformed mean ± SE of 3 biological and 3 technical replicates.

**Supplementary Fig. S13**. Validation of candidate genes *LRK10A* and *LRK10B* with qRT-PCR in transient assays. (A) Expression levels of *LRK10A* and *LRK10B* in RNAi knockdown transient assays conducted in *ca1ca1, Ca1ca1*, and *Ca1Ca1* cultivars. (B) Expression levels of *LRK10A* in overexpression vector constructs in *ca1ca1* and *Ca1ca1* cultivars. Error bars represent the standard deviation based on 3 biological replicates. Statistical analysis using a Student *t*-test indicated significant differences denoted as ****P* < 0.001, ***P* < 0.01, **P* < 0.05 when compared to the empty vector control in transient assay samples.

## Abbreviations

AFR: anthracnose fruit rot; ANOVA: analysis of variance; ARN: anthracnose root necrosis; BR: broad range; BUSCO: Benchmarking Universal Single-Copy Orthologs; H-1: haplotype-1; H-2: haplotype-2; HiFi: high fidelity; LAI: LTR Assembly Index; LLD: legume lectin domain; LSD: least significant difference; QTL, quantitative trait locus; SRA: sequence read archive; SSR: simple sequence repeat; T2T: telomere to telomere; TE: transposable element.
