# Supplementary material for: A telomere-to-telomere phased genome of an octoploid strawberry reveals a receptor kinase conferring anthracnose resistance
Source: Gigascience. 2025 Mar 12;14:giaf005. doi: 10.1093/gigascience/giaf005 (PMC11899574; doi:10.1093/gigascience/giaf005)
Supplement: giaf005_Supplemental_Files [file giaf005_supplemental_files.zip › Figure S10_Supplementary Material_Revised.pptx]

## Slide 1
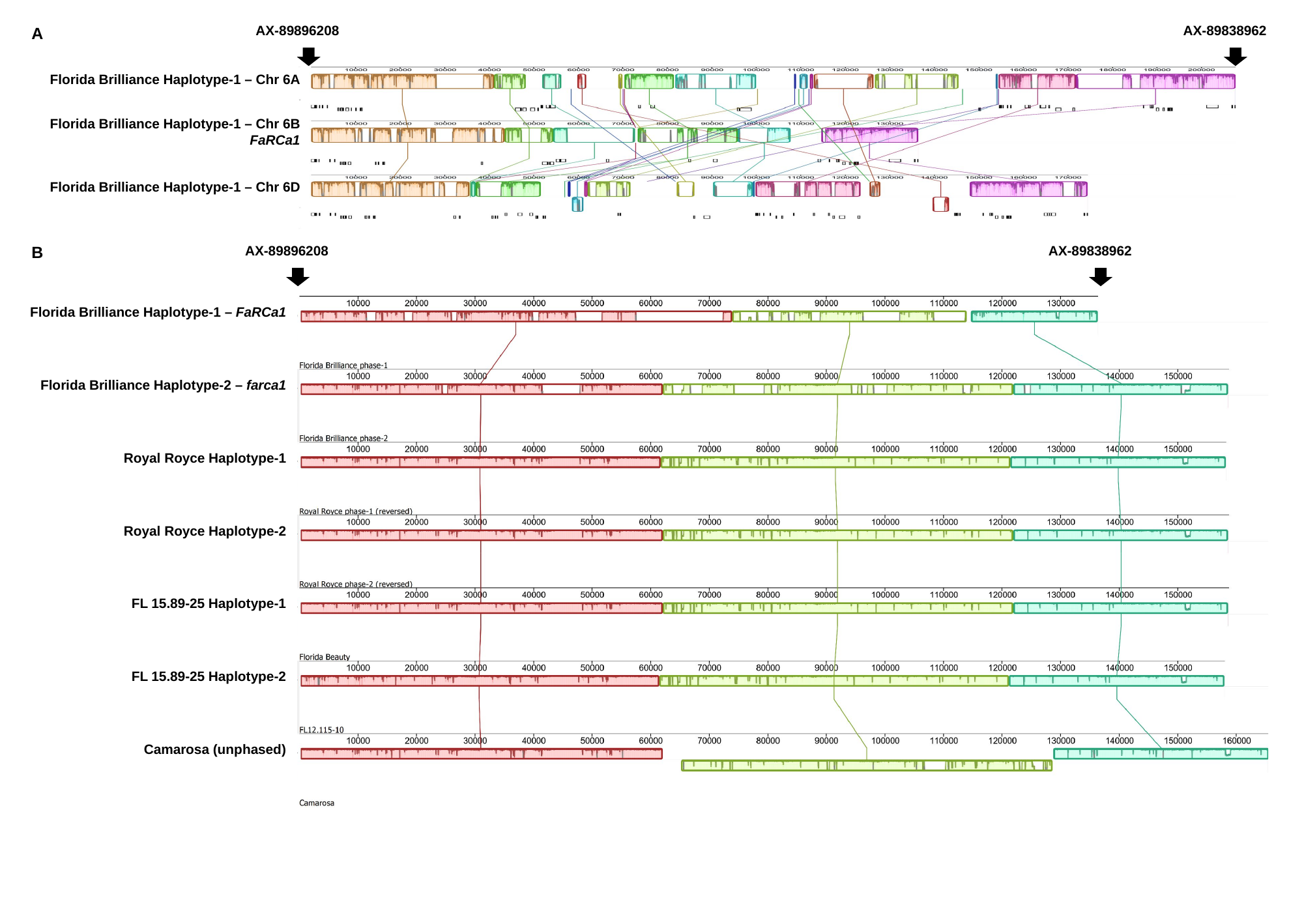

AX-89838962
AX-89896208
A
Florida Brilliance Haplotype-1 – Chr 6A
Florida Brilliance Haplotype-1 – Chr 6B
FaRCa1
Florida Brilliance Haplotype-1 – Chr 6D
B
AX-89838962
AX-89896208
Florida Brilliance Haplotype-1 – FaRCa1
Florida Brilliance Haplotype-2 – farca1
Royal Royce Haplotype-1
Royal Royce Haplotype-2
FL 15.89-25 Haplotype-1
FL 15.89-25 Haplotype-2
Camarosa (unphased)
