# Supplementary material for: A telomere-to-telomere phased genome of an octoploid strawberry reveals a receptor kinase conferring anthracnose resistance
Source: Gigascience. 2025 Mar 12;14:giaf005. doi: 10.1093/gigascience/giaf005 (PMC11899574; doi:10.1093/gigascience/giaf005)
Supplement: giaf005_Supplemental_Files [file giaf005_supplemental_files.zip › Figure S11_Supplementary Material_Revised.pptx]

## Slide 1
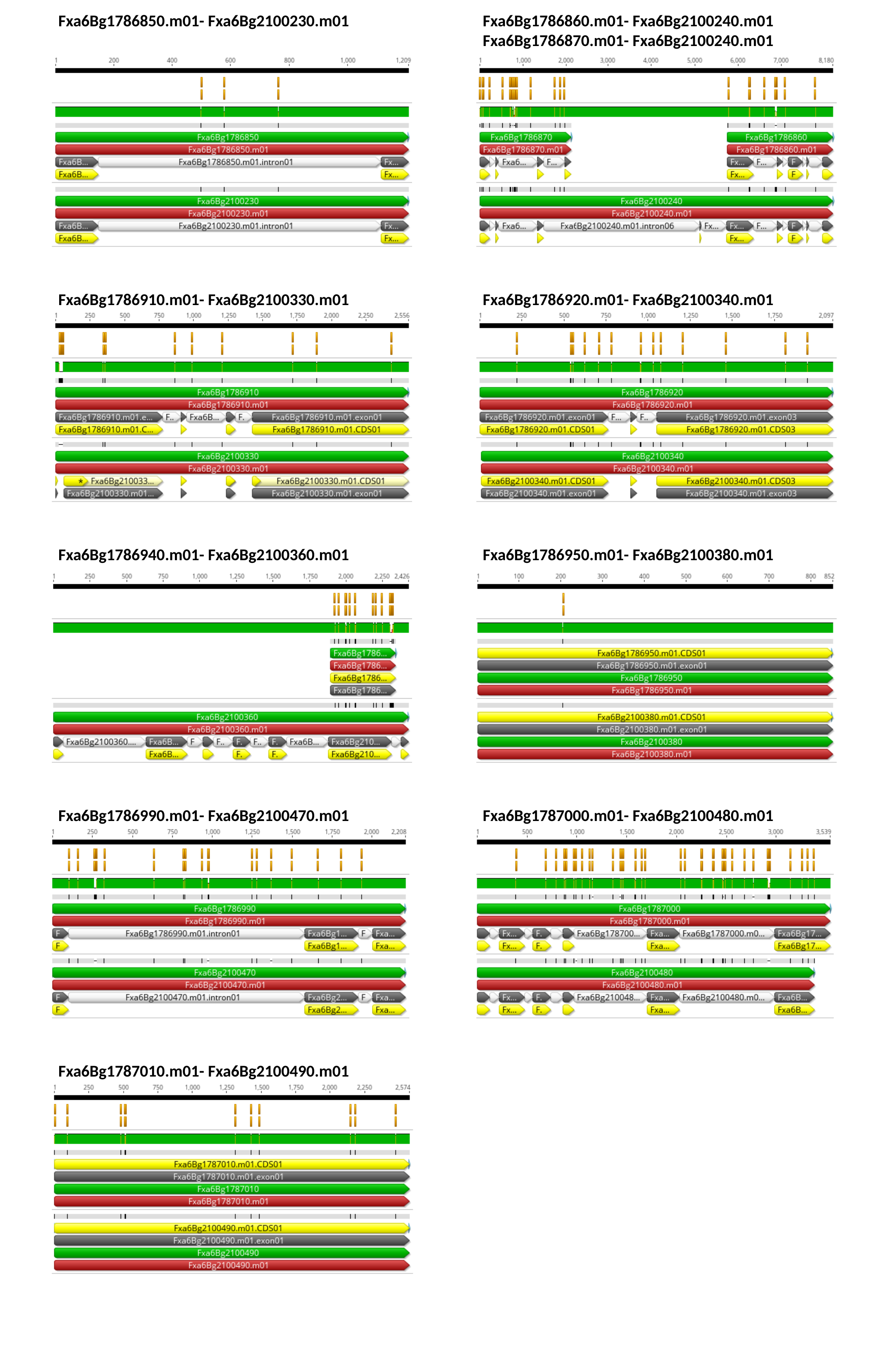

Fxa6Bg1786850.m01- Fxa6Bg2100230.m01
Fxa6Bg1786910.m01- Fxa6Bg2100330.m01
Fxa6Bg1786940.m01- Fxa6Bg2100360.m01
Fxa6Bg1786990.m01- Fxa6Bg2100470.m01
Fxa6Bg1787010.m01- Fxa6Bg2100490.m01
Fxa6Bg1786860.m01- Fxa6Bg2100240.m01
Fxa6Bg1786870.m01- Fxa6Bg2100240.m01
Fxa6Bg1786920.m01- Fxa6Bg2100340.m01
Fxa6Bg1786950.m01- Fxa6Bg2100380.m01
Fxa6Bg1787000.m01- Fxa6Bg2100480.m01
