# Supplementary material for: A telomere-to-telomere phased genome of an octoploid strawberry reveals a receptor kinase conferring anthracnose resistance
Source: Gigascience. 2025 Mar 12;14:giaf005. doi: 10.1093/gigascience/giaf005 (PMC11899574; doi:10.1093/gigascience/giaf005)
Supplement: giaf005_Supplemental_Files [file giaf005_supplemental_files.zip › Figure S12_Supplementary Material_Revised.pptx]

## Slide 1
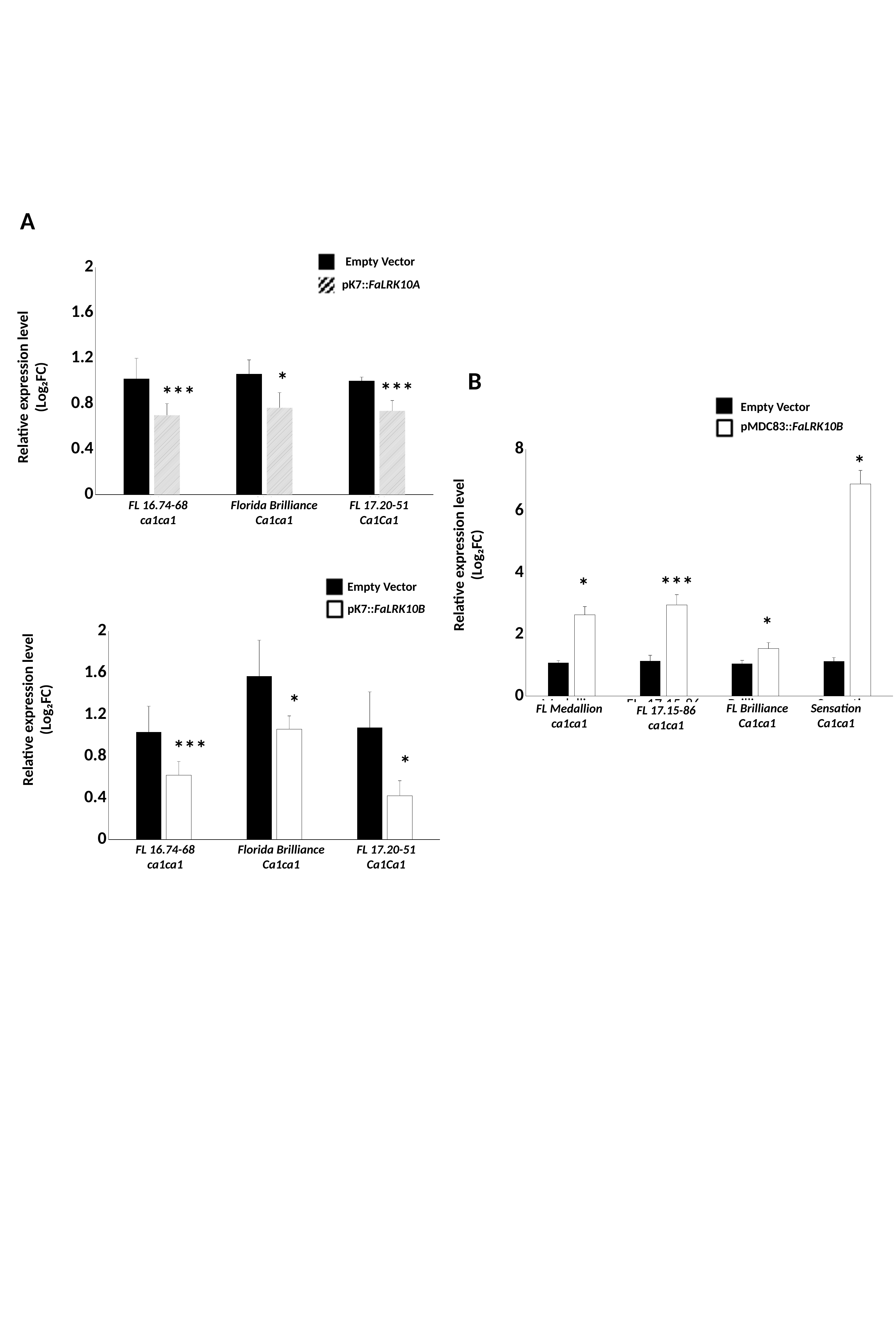

A
Empty Vector
pK7::FaLRK10A
### Chart
| Category | 163.26 | 163.26 |
|---|---|---|
| ca1ca1 | 1.0186257887805117 | 0.6969960223509011 |
| Ca1ca1 | 1.062106195213993 | 0.7628147681450455 |
| Ca1Ca1 | 1.0004745457445676 | 0.7358139849980857 |Relative expression level
(Log₂FC)
*
***
***
Florida Brilliance
Ca1ca1
FL 17.20-51
Ca1Ca1
FL 16.74-68
ca1ca1
Empty Vector
pK7::FaLRK10B
Relative expression level
(Log₂FC)
### Chart
| Category | 160 | 160 |
|---|---|---|
| ca1ca1 | 1.0318307201801955 | 0.6183555829824517 |
| Ca1ca1 | 1.5662972466503304 | 1.0576964288095536 |
| Ca1Ca1 | 1.0731519774363145 | 0.4183718515986795 |*
***
*
Florida Brilliance
Ca1ca1
FL 17.20-51
Ca1Ca1
FL 16.74-68
ca1ca1
B
Empty Vector
pMDC83::FaLRK10B
Relative expression level
(Log₂FC)
### Chart
| Category | EM | Target |
|---|---|---|
| Medallion | 1.074875189739316 | 2.6310651438635175 |
| FL_17.15-86 | 1.1317980241371448 | 2.950171077130113 |
| Brilliance | 1.0527324720794657 | 1.5408577188444648 |
| Sensation | 1.1283443242815878 | 6.873816232985198 |FL Brilliance
Ca1ca1
Sensation
Ca1ca1
FL Medallion
ca1ca1
FL 17.15-86
ca1ca1
*
***
*
*
