# Supplementary material for: A telomere-to-telomere phased genome of an octoploid strawberry reveals a receptor kinase conferring anthracnose resistance
Source: Gigascience. 2025 Mar 12;14:giaf005. doi: 10.1093/gigascience/giaf005 (PMC11899574; doi:10.1093/gigascience/giaf005)
Supplement: giaf005_Supplemental_Files [file giaf005_supplemental_files.zip › Figure S13_Supplementary Material_Revised.pptx]

## Slide 1
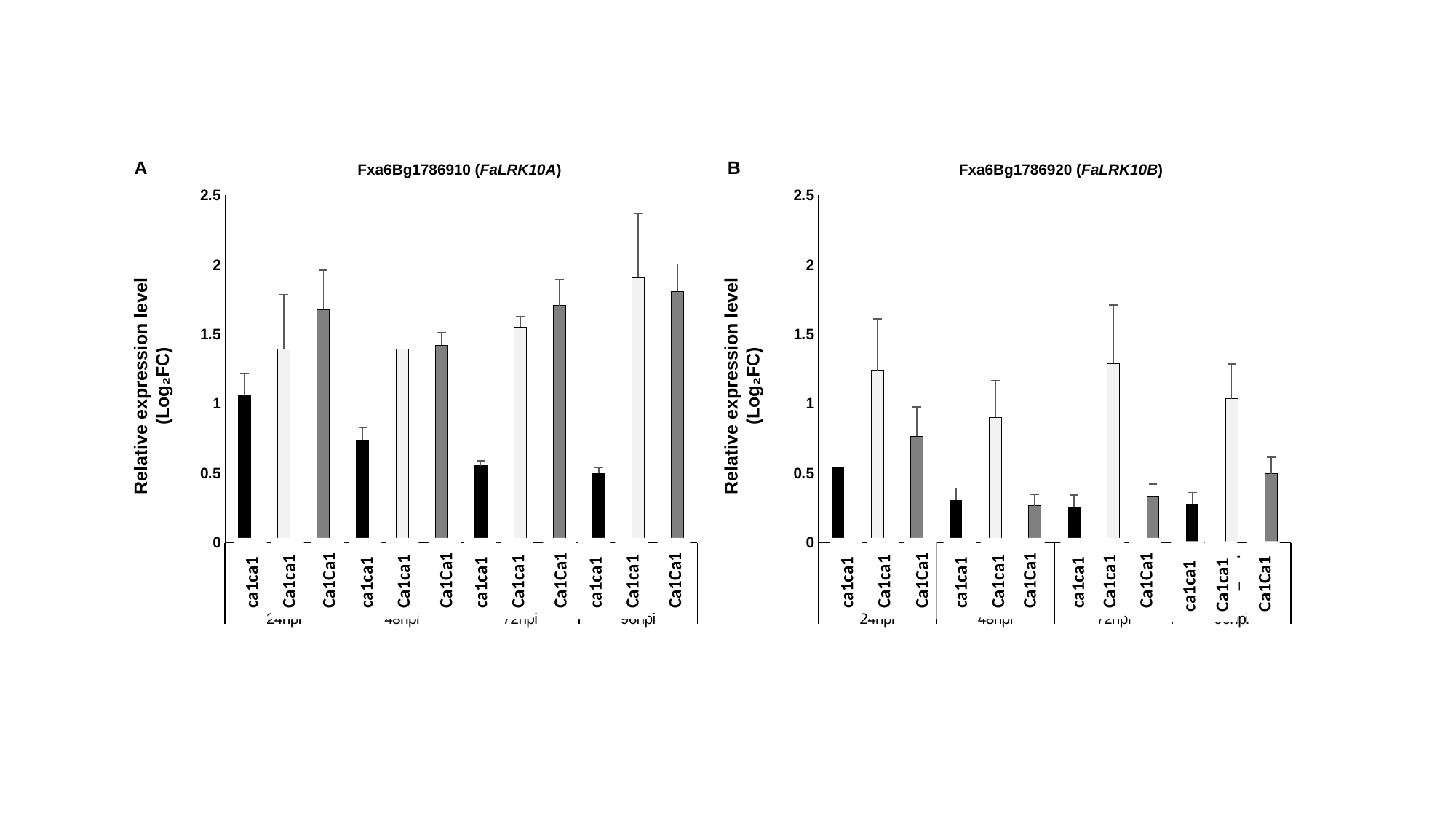

A
B
Fxa6Bg1786910 (FaLRK10A)
Relative expression level
(Log₂FC)
Relative expression level
(Log₂FC)
### Chart
| Category | |
|---|---|
| AA_24hpi | 1.0621985193841492 |
| AB_24hpi | 1.3943311876802122 |
| BB_24hpi | 1.6795872593921148 |
| AA_48hpi | 0.7394664820271516 |
| AB_48hpi | 1.3945891208378391 |
| BB_48hpi | 1.4230543834503413 |
| AA_72hpi | 0.5535063431199128 |
| AB_72hpi | 1.5520942725408642 |
| BB_72hpi | 1.70858496048026 |
| AA_96hpi | 0.5009650598488752 |
| AB_96hpi | 1.9100551365108522 |
| BB_96hpi | 1.8091080989647905 |
### Chart
| Category | |
|---|---|
| AA_24hpi | 0.543733487315252 |
| AB_24hpi | 1.2423353667931896 |
| BB_24hpi | 0.7663422500760982 |
| AA_48hpi | 0.30499991078000793 |
| AB_48hpi | 0.9019042036091633 |
| BB_48hpi | 0.266575923328957 |
| AA_72hpi | 0.2561958716236156 |
| AB_72hpi | 1.2917513664096985 |
| BB_72hpi | 0.3320976265691639 |
| AA_96hpi | 0.2787583658763345 |
| AB_96hpi | 1.036163690255848 |
| BB_96hpi | 0.497124777718089 |Fxa6Bg1786920 (FaLRK10B)
Ca1Ca1
ca1ca1
Ca1Ca1
Ca1Ca1
Ca1Ca1
Ca1Ca1
Ca1Ca1
ca1ca1
Ca1ca1
ca1ca1
Ca1ca1
ca1ca1
Ca1ca1
ca1ca1
Ca1ca1
Ca1ca1
ca1ca1
Ca1ca1
Ca1Ca1
ca1ca1
Ca1ca1
Ca1Ca1
ca1ca1
Ca1ca1
