# Supplementary material for: A telomere-to-telomere phased genome of an octoploid strawberry reveals a receptor kinase conferring anthracnose resistance
Source: Gigascience. 2025 Mar 12;14:giaf005. doi: 10.1093/gigascience/giaf005 (PMC11899574; doi:10.1093/gigascience/giaf005)
Supplement: giaf005_Supplemental_Files [file giaf005_supplemental_files.zip › Figure S2_Supplementary Material_Revised.pptx]

## Slide 1
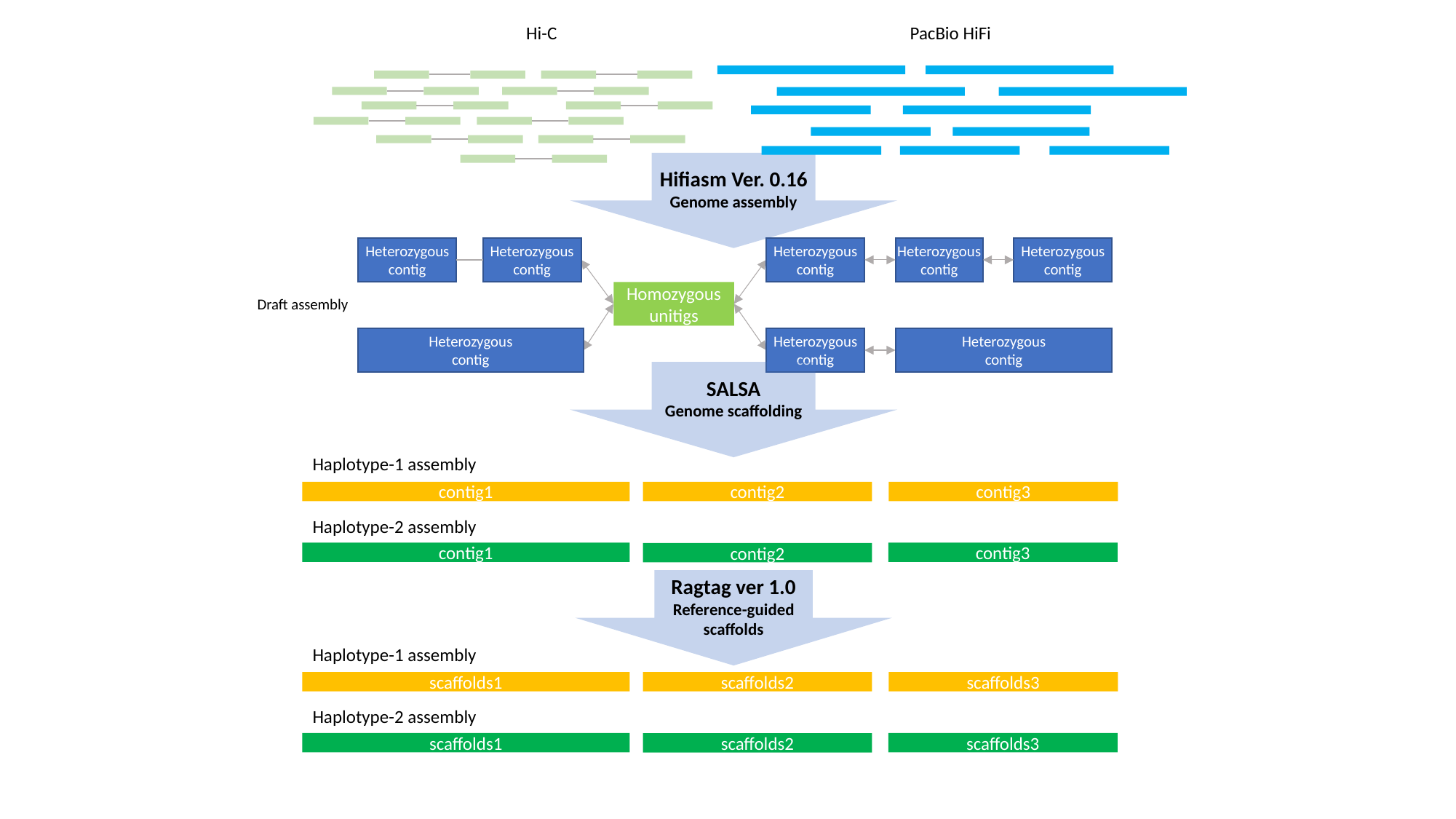

Hi-C
PacBio HiFi
Hifiasm Ver. 0.16
Genome assembly
Heterozygous
contig
Heterozygous
contig
Heterozygous
contig
Heterozygous
contig
Heterozygous
contig
Homozygous unitigs
Heterozygous
contig
Heterozygous
contig
Heterozygous
contig
Draft assembly
SALSA
Genome scaffolding
Haplotype-1 assembly
contig2
contig3
contig1
Haplotype-2 assembly
contig3
contig1
contig2
Ragtag ver 1.0
Reference-guided scaffolds
Haplotype-1 assembly
scaffolds2
scaffolds3
scaffolds1
Haplotype-2 assembly
scaffolds3
scaffolds1
scaffolds2
