# Supplementary material for: A telomere-to-telomere phased genome of an octoploid strawberry reveals a receptor kinase conferring anthracnose resistance
Source: Gigascience. 2025 Mar 12;14:giaf005. doi: 10.1093/gigascience/giaf005 (PMC11899574; doi:10.1093/gigascience/giaf005)
Supplement: giaf005_Supplemental_Files [file giaf005_supplemental_files.zip › Figure S4_Supplementary Material_Revised.pptx]

## Slide 1
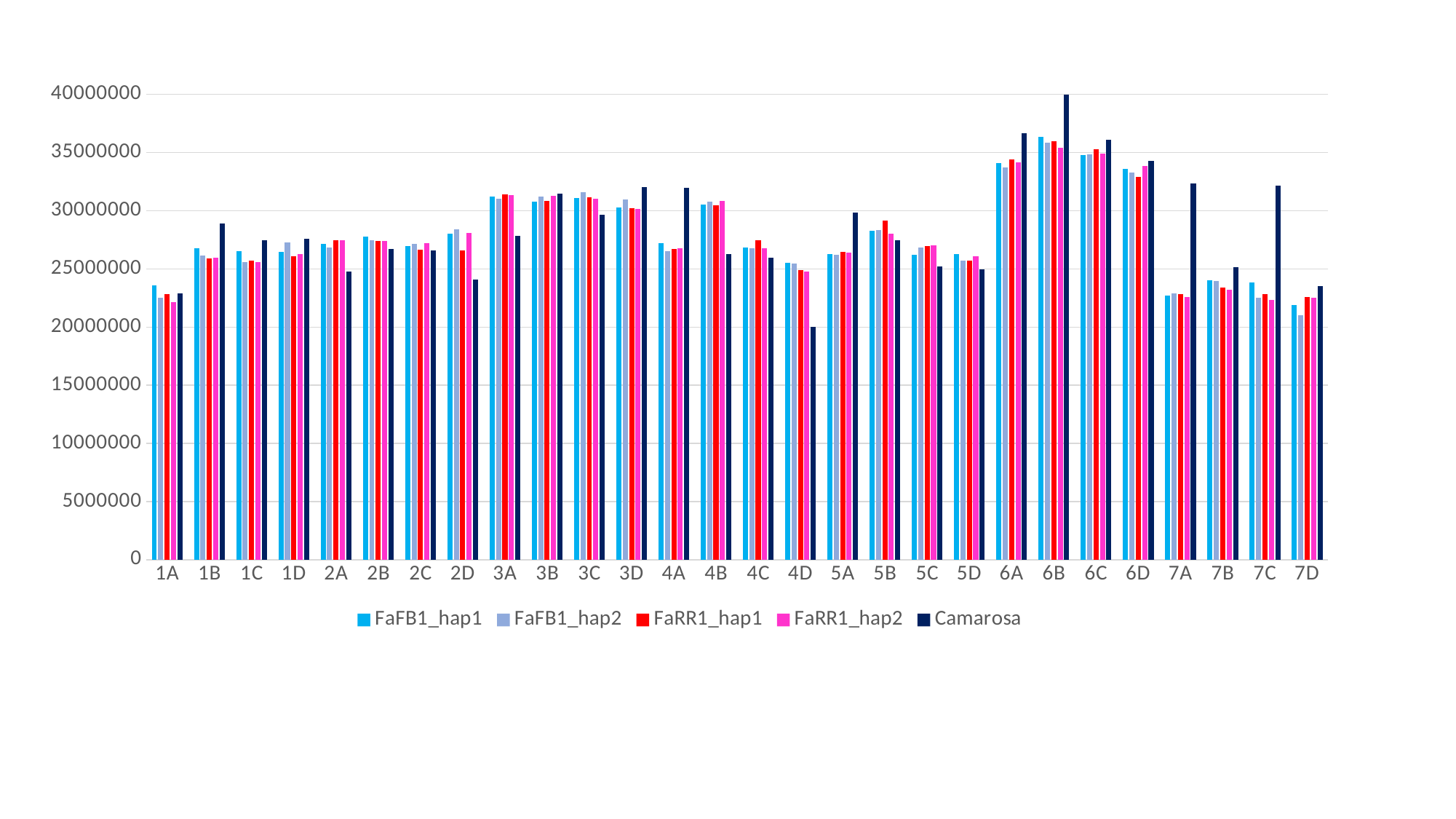

### Chart
| Category | FaFB1_hap1 | FaFB1_hap2 | FaRR1_hap1 | FaRR1_hap2 | Camarosa |
|---|---|---|---|---|---|
| 1A | 23567252.0 | 22531703.0 | 22855991.0 | 22149862.0 | 22887349.0 |
| 1B | 26785871.0 | 26139639.0 | 25894854.0 | 25931632.0 | 28910674.0 |
| 1C | 26527737.0 | 25588423.0 | 25722849.0 | 25566964.0 | 27436561.0 |
| 1D | 26433688.0 | 27297576.0 | 26068445.0 | 26286749.0 | 27594200.0 |
| 2A | 27144803.0 | 26862351.0 | 27491849.0 | 27487145.0 | 24782128.0 |
| 2B | 27757423.0 | 27483278.0 | 27418049.0 | 27417387.0 | 26692599.0 |
| 2C | 26941598.0 | 27140519.0 | 26662861.0 | 27223255.0 | 26582685.0 |
| 2D | 28016282.0 | 28417009.0 | 26601834.0 | 28082264.0 | 24073015.0 |
| 3A | 31194221.0 | 31023069.0 | 31413151.0 | 31325865.0 | 27809139.0 |
| 3B | 30755514.0 | 31218548.0 | 30825160.0 | 31256976.0 | 31459976.0 |
| 3C | 31090695.0 | 31575424.0 | 31136822.0 | 31011180.0 | 29626823.0 |
| 3D | 30266732.0 | 30950234.0 | 30209456.0 | 30162378.0 | 32005440.0 |
| 4A | 27193533.0 | 26546470.0 | 26690241.0 | 26804095.0 | 31955388.0 |
| 4B | 30511584.0 | 30781921.0 | 30438350.0 | 30856192.0 | 26295489.0 |
| 4C | 26858130.0 | 26806133.0 | 27481372.0 | 26803992.0 | 25974422.0 |
| 4D | 25535583.0 | 25468316.0 | 24913570.0 | 24790809.0 | 20034018.0 |
| 5A | 26252604.0 | 26180077.0 | 26468837.0 | 26406975.0 | 29826953.0 |
| 5B | 28272564.0 | 28334315.0 | 29153715.0 | 28042548.0 | 27452983.0 |
| 5C | 26214383.0 | 26828411.0 | 26974999.0 | 27044276.0 | 25211045.0 |
| 5D | 26255631.0 | 25736330.0 | 25732751.0 | 26110821.0 | 24981319.0 |
| 6A | 34110432.0 | 33700092.0 | 34436226.0 | 34154289.0 | 36657112.0 |
| 6B | 36373116.0 | 35862202.0 | 35982586.0 | 35436634.0 | 43627644.0 |
| 6C | 34799956.0 | 34853506.0 | 35269636.0 | 34899819.0 | 36124132.0 |
| 6D | 33615062.0 | 33281232.0 | 32927424.0 | 33827677.0 | 34274104.0 |
| 7A | 22721161.0 | 22875263.0 | 22827111.0 | 22554989.0 | 32354134.0 |
| 7B | 24003555.0 | 23978746.0 | 23370891.0 | 23234183.0 | 25137763.0 |
| 7C | 23810172.0 | 22525622.0 | 22854303.0 | 22340511.0 | 32186896.0 |
| 7D | 21919180.0 | 21016102.0 | 22595168.0 | 22543638.0 | 23534715.0 |
