# Supplementary material for: A telomere-to-telomere phased genome of an octoploid strawberry reveals a receptor kinase conferring anthracnose resistance
Source: Gigascience. 2025 Mar 12;14:giaf005. doi: 10.1093/gigascience/giaf005 (PMC11899574; doi:10.1093/gigascience/giaf005)
Supplement: giaf005_Supplemental_Files [file giaf005_supplemental_files.zip › Figure S8_Supplementary Material_Revised.pptx]

## Slide 1
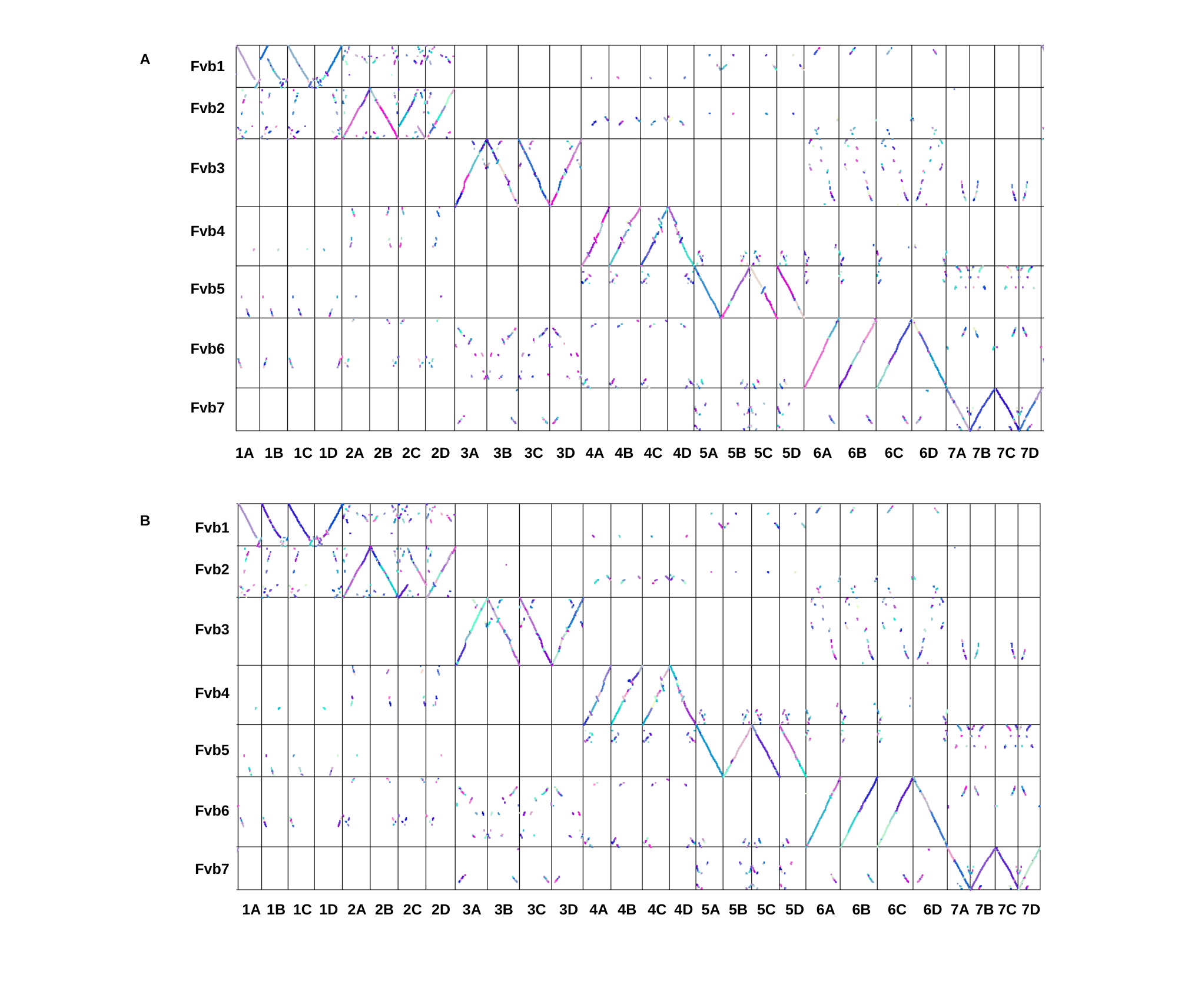

Fvb1
Fvb2
Fvb3
Fvb4
Fvb5
Fvb6
Fvb7
1A
1B
1C
1D
2A
2B
2C
2D
3A
3B
3C
3D
4A
4B
4C
4D
5A
5B
5C
5D
6A
6B
6C
6D
7A
7B
7C
7D
1A
1B
1C
1D
2A
2B
2C
2D
3A
3B
3C
3D
4A
4B
4C
4D
5A
5B
5C
5D
6A
6B
6C
6D
7A
7B
7C
7D
Fvb1
Fvb2
Fvb3
Fvb4
Fvb5
Fvb6
Fvb7
A
B
