# Supplementary material for: A telomere-to-telomere phased genome of an octoploid strawberry reveals a receptor kinase conferring anthracnose resistance
Source: Gigascience. 2025 Mar 12;14:giaf005. doi: 10.1093/gigascience/giaf005 (PMC11899574; doi:10.1093/gigascience/giaf005)
Supplement: giaf005_Supplemental_Files [file giaf005_supplemental_files.zip › Supplementary table_vf_Revised.docx]

**Supplementary tables**

**Table S1**. Libraries sequenced and used in assembly with accession numbers.

**Table S2**. Statistics of genome assembly and Hi-C analysis for octoploid strawberry ‘Florida Brilliance’.

**Table S3.** LTR Assembly Index (LAI) scores for ‘Florida Brilliance’ phase-1 and phase-2 subgenomes.

**Table S4.** Classification and distribution of repetitive DNA elements identified in the genome assembly for ‘Florida Brilliance’ by EDTA pipeline.

**Table S5.** Simple sequence repeats of octoploid strawberry genome assembly for ‘Florida Brilliance’ using SSR Finder.

**Table S6**. Benchmarking universal single-copy orthologs (BUSCO) analysis of 321,419 transcriptome assemblies for ‘Florida Brilliance’.

**Table S7**. Genes predicted in the H-1 and H-2 genome assemblies of 'Florida Brilliance'.

**Table S8**. Genes predicted in the FaFB1 of ‘Florida Brilliance’.

**Table S9**. Genes predicted in the FaRR1 of ‘Royal Royce’.

**Table S10**. Paired T test results of the number of genes between sub-genomes (A, B, C, and D). Significant differences (*P*<0.05) in gene numbers between sub-genomes are indicated by asterisks.

**Table S11.** Genes located in the 136 kb *FaRCa1* region in Chr 6B from the ‘Florida Brilliance’. Gene description corresponds to the first alignment result from BLAST in the NCBI webpage. Genes with different positions in both annotations are shown with an *

**Table S12.** List of sequences used for RNAi vector construction and transformation verification.

**Table S13**. Statistics of HiFi reads (QV ≥ 20) generated from iso-seq sequences from 6 tissues of ‘Florida Brilliance’.

**Table S14.** List of primers sequences used for qRT-PCR.

**Table S1**. Libraries sequenced and used in assembly with accession numbers.

| **Library type** | **Tissue** | **No. of sequencing runs** | **Number of reads (#)** | **Average of read length (bp)** | **Number of bases (Gb)** |
| --- | --- | --- | --- | --- | --- |
| Pacbio HiFi | Leaf | 5 | 9,101,332 | 15,834.8 | 144.10 |
| Hi-C | Leaf | 2 | 572,765,774 | 151 | 86.50 |

**Table S2**. Statistics of genome assembly and Hi-C analysis for octoploid strawberry ‘Florida Brilliance’.

| **Category** | **Statistics** | **Proportion (%)** |
| --- | --- | --- |
| Number of R1 reads mapped | 265,085,428 | 92.6 |
| Number of R2 reads mapped | 262,663,135 | 91.7 |
| Number of uniquely mapped pairs | 128,100,643 | 44.7 |
| Number of unmapped read pairs | 8,661,036 | 3.0 |
| Number of low quality of pairs | 121,926,069 | 42.6 |
| Number of pairs with singleton | 27,695,139 | 9.7 |
| Total number of pairs processed | 286,382,887 | 100.0 |

**Table S3.** LTR Assembly Index (LAI) scores for ‘Florida Brilliance’ phase-1 and phase-2 subgenomes.

| **Genome assembly** | **Sub-genome** | **Haplotype phase** | **LAI** |
| --- | --- | --- | --- |
| Florida Brilliance | A | Haplotype-1 | 19.72 |
| Florida Brilliance | A | Haplotype-2 | 19.27 |
| Florida Brilliance | B | Haplotype-1 | 17.17 |
| Florida Brilliance | B | Haplotype-2 | 18.44 |
| Florida Brilliance | C | Haplotype-1 | 18.83 |
| Florida Brilliance | C | Haplotype-2 | 19.17 |
| Florida Brilliance | D | Haplotype-1 | 19.75 |
| Florida Brilliance | D | Haplotype-2 | 20.13 |
| Total |  |  | 19.72 |

**Table S4.** Classification and distribution of repetitive DNA elements identified in the genome assembly for ‘Florida Brilliance’ by EDTA pipeline.

|  | **Class** | **Sub-Class** | **Proportion of masked sequence (%)** | | | |
| --- | --- | --- | --- | --- | --- | --- |
|  |  |  | **Florida Brilliance** | ***F*. *Chiloensis*** | **Benihoppe** | **Yanli** |
| LTR | Class I |  |  |  |  |  |
|  |  | Copia | 4.69 | 6.28 | 6.67 | 6.68 |
|  |  | Gypsy | 11.77 | 13.31 | 13.07 | 12.96 |
|  |  | unknown | 9.55 | 5.87 | 5.59 | 7.91 |
| TIR | Class II |  |  |  |  |  |
|  |  | CACTA | 3.78 | 6.28 | 4.73 | 3.28 |
|  |  | Mutator | 4.31 | 4.00 | 3.53 | 3.82 |
|  |  | PIF_Harbinger | 1.32 | 1.29 | 1.25 | 1.27 |
|  |  | Tc1_Mariner | 0.11 | 0.13 | 0.12 | 0.11 |
|  |  | hAT | 2.12 | 2.49 | 2.24 | 2.28 |
| NonTIR | Class II |  |  |  |  |  |
|  |  | helitron | 4.00 | 4.66 | 6.10 | 4.22 |
| **Total** | - | - | 41.66 | 44.33 | 43.30 | 42.54 |

**Table S5.** Simple sequence repeats of octoploid strawberry genome assembly for ‘Florida Brilliance’ using SSR Finder.

| **SSR type** | **Frequency (#)** | **Proportion (%)** | **Size (bp)** | **Genome coverage^z^ (%)** |
| --- | --- | --- | --- | --- |
| Dinucleotide | 80,951 | 36.29 | 820,359 | 0.05 |
| Trinucleotide | 52,060 | 23.34 | 593,417 | 0.04 |
| Tetranucleotide | 65,389 | 29.31 | 737,447 | 0.05 |
| Pentanucleotide | 15,126 | 6.78 | 217,899 | 0.01 |
| Hexanucleotide | 9,533 | 4.27 | 164,112 | 0.01 |
| Total | 223,060 | 100.00 | 2,533,234 | 0.16 |

**Table S6**. Benchmarking universal single-copy orthologs (BUSCO) analysis of 321,419 transcriptome assemblies for ‘Florida Brilliance’.

|  | embryophyta_odb10 | eudicots_odb10 |
| --- | --- | --- |
| Complete BUSCOs (C) | 1,573 (97.5%) | 2,241 (96.3%) |
| Complete and single-copy BUSCOs (S) | 46 (2.9%) | 82 (3.5%) |
| Complete and duplicated BUSCOs (D) | 1,527 (94.6%) | 2,159 (92.8%) |
| Fragmented BUSCOs (F) | 18 (1.1%) | 22 (0.9%) |
| Missing BUSCOs (M) | 23 (1.4%) | 63 (2.8%) |
| Total BUSCO groups searched | 1,614 (100.0%) | 2,326 (100.0%) |

**Table S7**. Genes predicted in the H-1 and H-2 genome assemblies of 'Florida Brilliance'.

| **Chromosome** | **Sub-genome in phased-1 assembly** | | | | | **Sub-genome in phased-2 assembly** | | | | |
| --- | --- | --- | --- | --- | --- | --- | --- | --- | --- | --- |
|  | **A** | **B** | **C** | **D** | **Total** | **A** | **B** | **C** | **D** | **Total** |
| Chr01 | 3,312 | 3,055 | 3,085 | 2,611 | 12,063 | 3,183 | 2,974 | 2,935 | 3,037 | 12,129 |
| Chr02 | 3,741 | 3,768 | 3,149 | 3,294 | 13,952 | 4,033 | 3,768 | 3,593 | 3,726 | 15,120 |
| Chr03 | 3,830 | 3,850 | 3,879 | 3,463 | 15,022 | 3,908 | 3,931 | 3,943 | 3,231 | 15,013 |
| Chr04 | 3,268 | 2,956 | 2,789 | 2,990 | 12,003 | 3,491 | 3,402 | 3,211 | 2,891 | 12,995 |
| Chr05 | 3,747 | 3,676 | 3,281 | 3,314 | 14,018 | 3,754 | 3,315 | 3,448 | 3,321 | 13,838 |
| Chr06 | 4,691 | 4,297 | 4,089 | 4,398 | 17,475 | 5,002 | 4,752 | 4,550 | 4,416 | 18,720 |
| Chr07 | 3,338 | 2,858 | 3,356 | 2,626 | 12,178 | 3,385 | 3,199 | 2,936 | 2,914 | 12,434 |
| Total | 25,927 | 24,460 | 23,628 | 22,696 | 96,711 | 26,756 | 25,341 | 24,616 | 23,536 | 100,249 |
| Percentage (%) | 27.0 | 25. 5 | 24.6 | 23.6 | 100 | 27.8 | 26.4 | 25.6 | 24.5 | 100 |

**Table S8**. Genes predicted in the FaFB1 of ‘Florida Brilliance’.

| **Chromosome** | **Sub-genome** | | | | |
| --- | --- | --- | --- | --- | --- |
|  | **A** | **B** | **C** | **D** | **Total** |
| Chr01 | 3,312 | 2,974 | 2,935 | 2,611 | 11,832 |
| Chr02 | 3,741 | 3,768 | 3,149 | 3,294 | 13,952 |
| Chr03 | 3,908 | 3,931 | 3,879 | 3,231 | 14,949 |
| Chr04 | 3,268 | 2,956 | 2,789 | 2,990 | 12,003 |
| Chr05 | 3,754 | 3,315 | 3,448 | 3,321 | 13,838 |
| Chr06 | 4,691 | 4,297 | 4,089 | 4,398 | 17,475 |
| Chr07 | 3,338 | 2,858 | 3,356 | 2,626 | 12,178 |
| Total | 26,012 | 24,099 | 23,645 | 22,471 | 96,227 |
| Percentage (%) | 27.0 | 25.0 | 24.6 | 23.4 | 100 |

**Table S9**. Genes predicted in the FaRR1 of ‘Royal Royce’.

| **Chromosome** | **Sub-genome** | | | | |
| --- | --- | --- | --- | --- | --- |
|  | **A** | **B** | **C** | **D** | **Total** |
| Chr01 | 3,107 | 2,947 | 3,062 | 2,957 | 12,073 |
| Chr02 | 4,027 | 3,788 | 3,532 | 3,612 | 14,959 |
| Chr03 | 4,353 | 3,907 | 3,845 | 3,713 | 15,818 |
| Chr04 | 3,582 | 3,359 | 3,142 | 2,938 | 13,021 |
| Chr05 | 3,800 | 3,629 | 3,236 | 3,337 | 14,002 |
| Chr06 | 5,173 | 4,663 | 4,610 | 4,441 | 18,887 |
| Chr07 | 3,356 | 3,168 | 3,078 | 2,913 | 12,515 |
| Total | 27,398 | 25,461 | 24,505 | 23,911 | 101,275 |
| Percentage (%) | 27.1 | 25.1 | 24.2 | 23.6 | 100 |

**Table S10**. Paired T test results of the number of genes between sub-genomes (A, B, C, and D). Significant differences (*P*<0.05) in gene numbers between sub-genomes are indicated by asterisks.

|  |  | A | B | C | D |
| --- | --- | --- | --- | --- | --- |
| Paired t-test | A | 1 | 0.007^*^ | 0.006^*^ | 0.0002^*^ |
|  | B | - | 1 | 0.31 | 0.04^*^ |
|  | C | - | - | 1 | 0.16 |
|  | D | - | - | - | 1 |

**Table S11.** Genes located in the 136 kb *FaRCa1* region in Chr 6B from the ‘Florida Brilliance’. Gene description corresponds to the first alignment result from BLAST in the NCBI webpage. Genes with different positions in both annotations are shown with an *

| No | Gene Name | Minimum | Maximum | Length | Direction | Gene description | Sequence ID |
| --- | --- | --- | --- | --- | --- | --- | --- |
| 1 | Fxa6Bg1786850 | 16189412 | 16190620 | 1209 | F | uncharacterized protein LOC112189577 [*Rosa chinensis*] | XP_024184692 |
| 2 | Fxa6Bg1786860 | 16191808 | 16194205 | 2398 | R | PREDICTED: magnesium transporter MRS2-1 [*Fragaria vesca* subsp. *vesca*] | XP_004303233 |
| 3 | Fxa6Bg1786870 | 16199664 | 16201750 | 2087 | R | PREDICTED: chaperone protein dnaJ 8, chloroplastic isoform X2 [*Fragaria vesca* subsp. *vesca*] | XP_004303232 |
| 4 | Fxa6Bg1786890 | 16223504 | 16223815 | 312 | R | Not found |  |
| 5 | Fxa6Bg1786910 | 16237200 | 16239755 | 2556 | R | rust resistance kinase Lr10-like isoform X1 [*Prunus dulcis*] | XP_034221356 |
| 6 | Fxa6Bg1786920 | 16240331 | 16242427 | 2097 | F | rust resistance kinase Lr10-like [*Prunus persica*] | XP_020421274 |
| 7 | Fxa6Bg1786930 | 16251583 | 16252122 | 540 | F | PREDICTED: uncharacterized protein LOC101315290 [*Fragaria vesca* subsp. *vesca*] | XP_004308434 |
| 8 | Fxa6Bg1786940 | 16270090 | 16270524 | 435 | F | PREDICTED: shikimate O-hydroxycinnamoyltransferase [*Fragaria vesca* subsp. *vesca*] | XP_011467012 |
| 9 | Fxa6Bg1786950 | 16275586 | 16276437 | 852 | F | PREDICTED: shikimate O-hydroxycinnamoyltransferase [*Fragaria vesca* subsp. *vesca*] | XP_011467012 |
| 10 | Fxa6Bg1786980 | 16299129 | 16299701 | 573 | F | putative nucleotidyltransferase, Ribonuclease H [*Rosa chinensis*] | PRQ38517 |
| 11 | Fxa6Bg1786990 | 16301656 | 16303863 | 2208 | F | PREDICTED: uncharacterized protein LOC101296401 isoform X1 [*Fragaria vesca* subsp. *vesca*] | XP_004303225 |
| 12 | Fxa6Bg1787000 | 16304628 | 16308157 | 3530 | R | PREDICTED: uncharacterized protein LOC101296120 [*Fragaria vesca* subsp. *vesca*] | XP_004303224 |
| 13 | Fxa6Bg1787010 | 16315102 | 16317675 | 2574 | F | PREDICTED: pentatricopeptide repeat-containing protein At3g02330 [*Fragaria vesca* subsp. *vesca*] | XP_004303223 |

**Table S12.** List of sequences used for RNAi vector construction and transformation verification.

| No | Name | DNA sequence (5’-3’) | Purpose |
| --- | --- | --- | --- |
| 1 | *FaCa1*-Fxa6Bg1786920-RNAi | ggggACAAGTTTGTACAAAAAAGCAGGCTATGCAGAAAATGCTTCTGAATTTGTTATGCTGTTGGTTTCTCATAGGCGCAGTGCTAGATGTAGATATTATTGTGCATGGAGAATCAGTTGGCCCTGAAAATTGCACAGAGGTTCGATGTAAACGGCATGGCCCTGTGATCCGATTCCCATTTCGACTGAAAGGAATGCAACCACTCCATTGCGGCTACCCGGGCTTTGATCTGTCATGCACCACGGACAAGCAGACTGTGCTCCAGATCAACTCATCATCAGCTGACAAGTTCTTGGTTAAAAGGATTGACTACACTGCTCAGGAAATTGAAATATATCTTTATAATAGTCACTATGATTATTTCAATGATTTCCAGTATATCTGTATTCCTAGACAGATTTTCGAGCTCAGTTCCTCTCCATTCGAATAATTTCCTGAGCAGTGTAGTCAATCCTTTTAACCAAGAACTTGTCAGCTGATGATGAGTTGATCTGGAGCACAGTCTGCTTGTCCGTGGTGCATGACAGATCAAAGCCCGGGTAGCCGCAATGGAGTGGTTGCATTCCTTTCAGTCGAAATGGGAATCGGATCACAGGGCCATGCCGTTTACATCGAACCTCTGTGCAATTTTCAGGGCCAACTGATTCTCCATGCACAATAATATCTACATCTAGCACTGCGCCTATGAGAAACCAACAGCATAACAAATTCAGAAGCATTTTCTGCATACCCAGCTTTCTTGTACAAAGTGGTcccc | RNAi |
| 2 | *FaCa1*-Fxa6Bg1786910-RNAi | ggggACAAGTTTGTACAAAAAAGCAGGCTATGGCTTGTATAGAGCCACATAACATAAACAATCTCCCAAACATTTCTATTTACCCTTATCATCTCTGTTTCATTACGCCTTCTTCCTGCTCACCATGTCTAGAAGAAGTCTCCTTTTTGCTTCTTACAGTTATTTCACAATCGTCGGATACCGGAAATCCTTTAGATATCTGTACCTCTTCTTGTGGAGGTATTTACATAAGCTATCCATTTCGACTGAAAGGAGACCCTCAAAACTGTGGAAACGAAAAGTTTGAGCTGTCTTGCGAGGCAAATAGTACTACTGCAGTACTATACTTGGAATCAGAAGAGTATTATGTGAAGTCGATCCATTACAATAACTTCACAATCCGAATCGTGGATTCCGGTGTTCAGAAGAAGCGGAACTACTGCTCCACCGCAAGTATAGTACTGCAGTAGTACTATTTGCCTCGCAAGACAGCTCAAACTTTTCGTTTCCACAGTTTTGAGGGTCTCCTTTCAGTCGAAATGGATAGCTTATGTAAATACCTCCACAAGAAGAGGTACAGATATCTAAAGGATTTCCGGTATCCGACGATTGTGAAATAACTGTAAGAAGCAAAAAGGAGACTTCTTCTAGACATGGTGAGCAGGAAGAAGGCGTAATGAAACAGAGATGATAAGGGTAAATAGAAATGTTTGGGAGATTGTTTATGTTATGTGGCTCTATACAAGCCATACCCAGCTTTCTTGTACAAAGTGGTcccc | RNAi |
| 3 | Fxa6Bg1786920_F1 | CTTCTGAATTTGTTATGCTGTT | construct validation |
| 4 | Fxa6Bg1786920_R1 | CGTTTACATCGAACCTCTG | construct validation |
| 5 | Fxa6Bg1786910_F2 | GCTTGTATAGAGCCACATAAC | construct validation |
| 6 | Fxa6Bg1786910_R2 | CATGGTGAGCAGGAAGA | construct validation |

**Table S13**. Statistics of HiFi reads (QV ≥ 20) generated from iso-seq sequences from 6 tissues of ‘Florida Brilliance’.

| **Tissue** | **Yield (bp)** | **Number of reads** | **Average length** |
| --- | --- | --- | --- |
| Leaf | 125,573,371 | 50,973 | 2,463.5 |
| Crown | 138,458,257 | 56,707 | 2,441.6 |
| Flower | 120,850,566 | 51,203 | 2,360.2 |
| Green fruit | 132,446,690 | 54,049 | 2,450.5 |
| Red fruit | 132,761,812 | 49,638 | 2,674.6 |
| Root | 116,103,170 | 45,577 | 2,547.4 |
| Total | 766,193,866 | 308,147 |  |
| Average |  |  | 2,486.5 |

**Table S14.** List of primers sequences used for qRT-PCR.

| No | Name | Primer Sequences (5'-3') |
| --- | --- | --- |
| 1 | FaGapDH2-F | CCCAAGTAAGGATGCCCCCATGTTCG |
|  | FaGapDH2-R | TTGGCAAGGGGAGCAAGACAGTTGGTAG |
| 2 | Fxa6Bg1786920-F1 | TTCTCAGACGCCCAAAGAAA |
|  | Fxa6Bg1786920-R1 | AGTGGACAGAAGGATGGAGA |
| 3 | Fxa6Bg1786910-F3 | CCCGTTAGCTTGGGACTTAC |
|  | Fxa6Bg1786910-R3 | AATTGGGTCAAGGAGGCTATG |
