# Supplementary figures and images for: A telomere-to-telomere phased genome of an octoploid strawberry reveals a receptor kinase conferring anthracnose resistance
Source: Gigascience. 2025 Mar 12;14:giaf005. doi: 10.1093/gigascience/giaf005 (PMC11899574; doi:10.1093/gigascience/giaf005)

## Slide 1
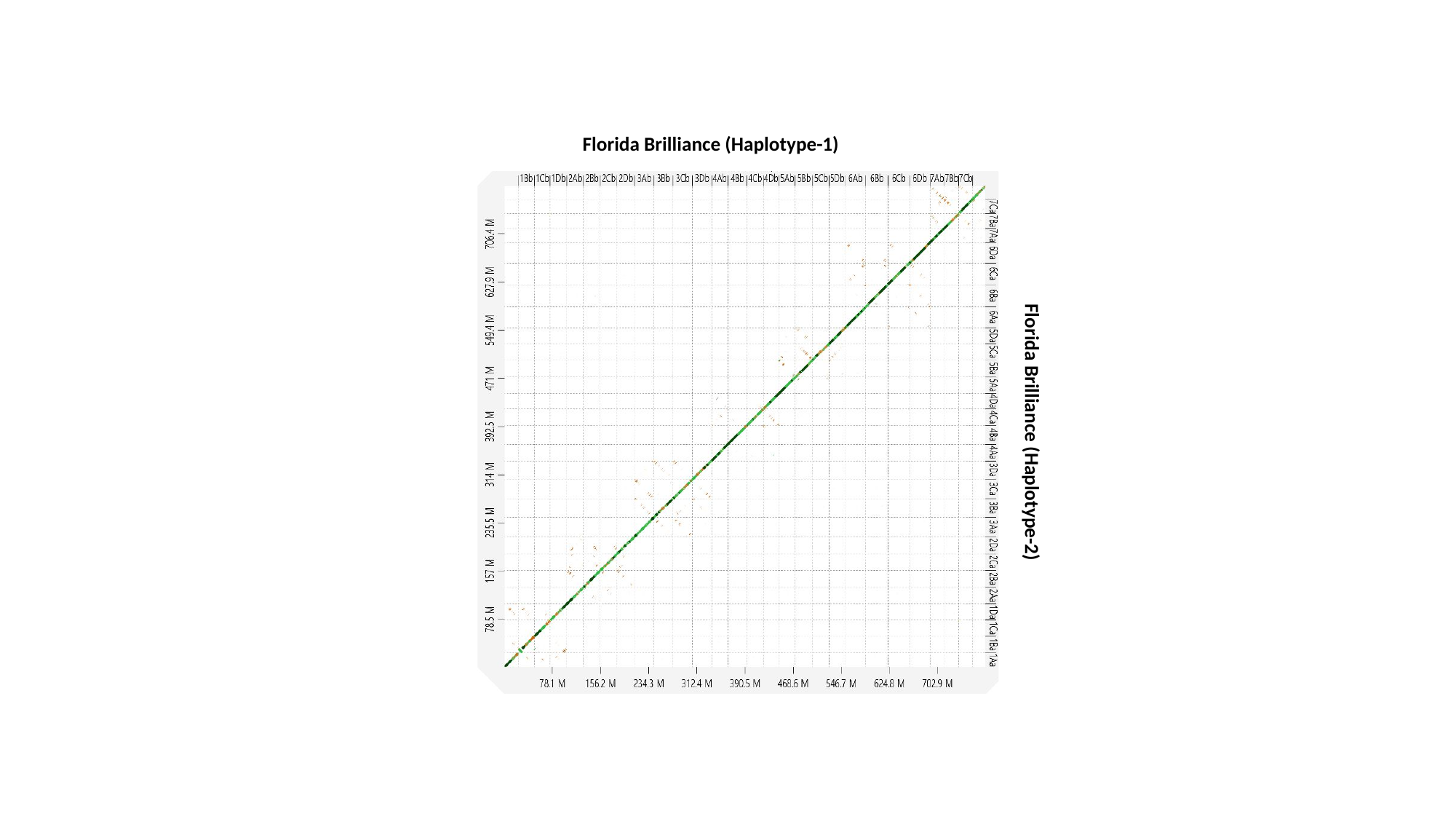

Florida Brilliance (Haplotype-1)
Florida Brilliance (Haplotype-2)

Supplement: giaf005_Supplemental_Files [file giaf005_supplemental_files.zip › Figure S1_Supplementary Material_Revised.pptx]

## Slide 1
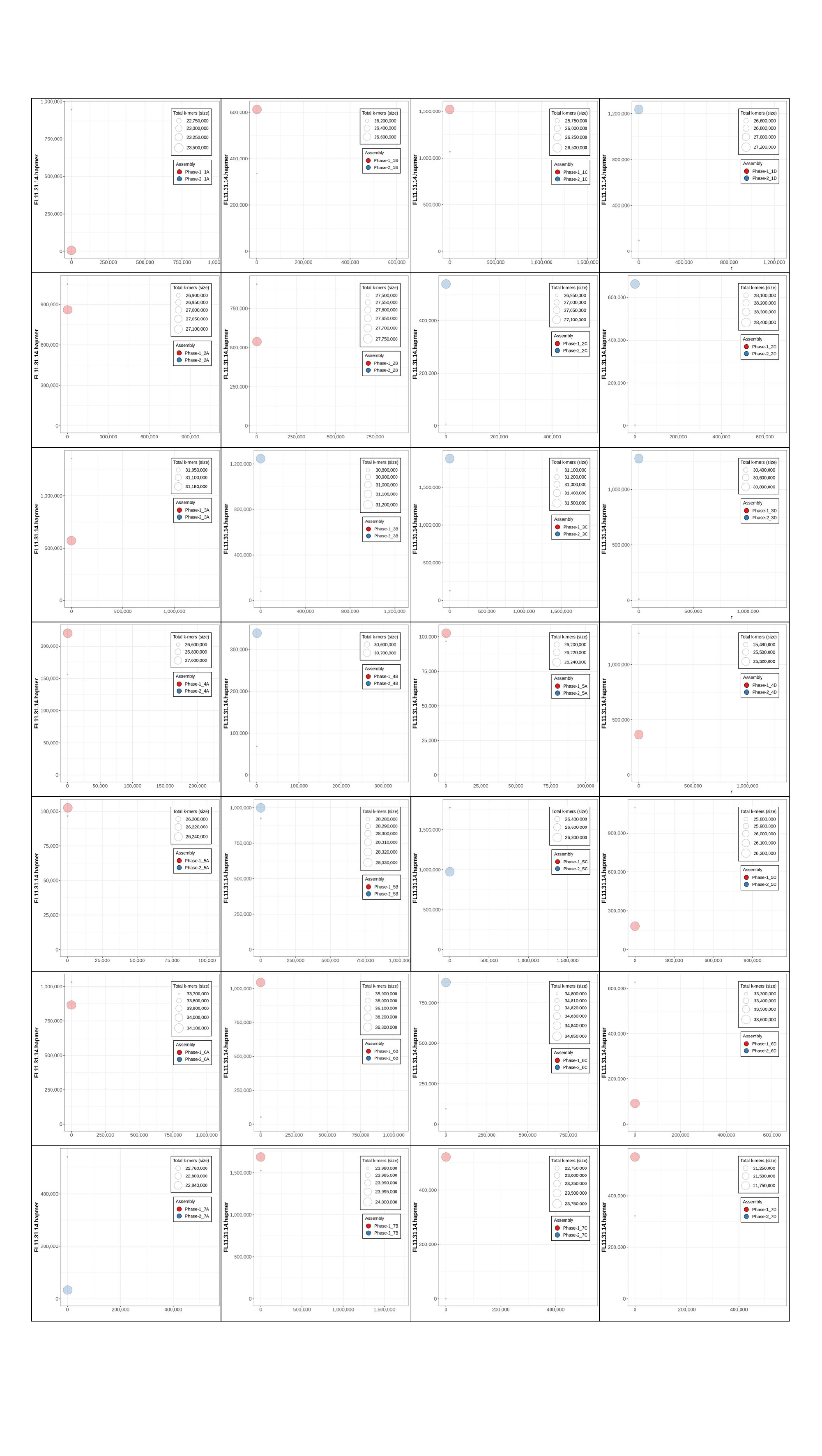

Supplement: giaf005_Supplemental_Files [file giaf005_supplemental_files.zip › Figure S3_Supplementary Material_Revised.pptx]

## Slide 1
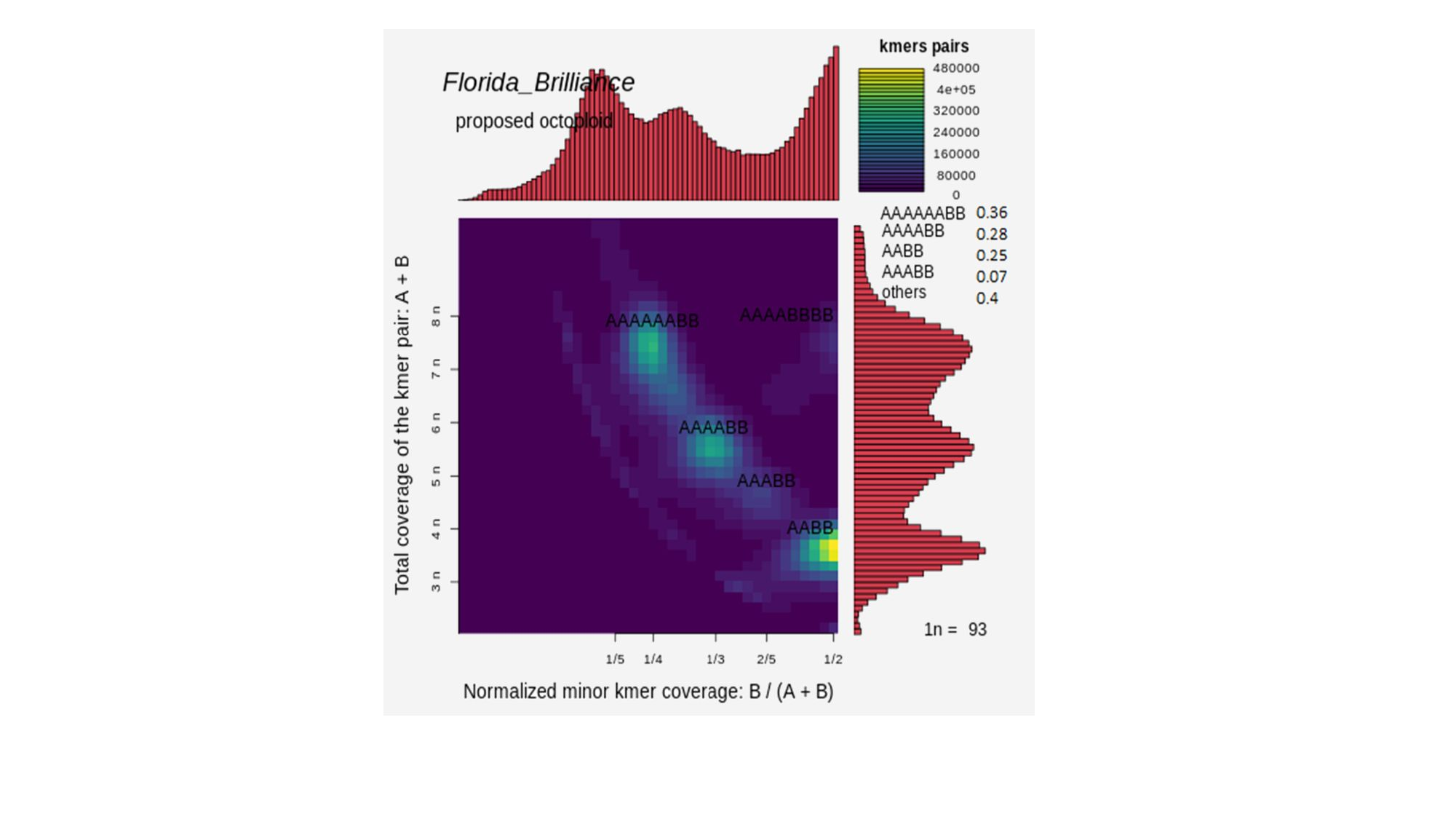

Supplement: giaf005_Supplemental_Files [file giaf005_supplemental_files.zip › Figure S5_Supplementary Material_Revised.pptx]

## Slide 1
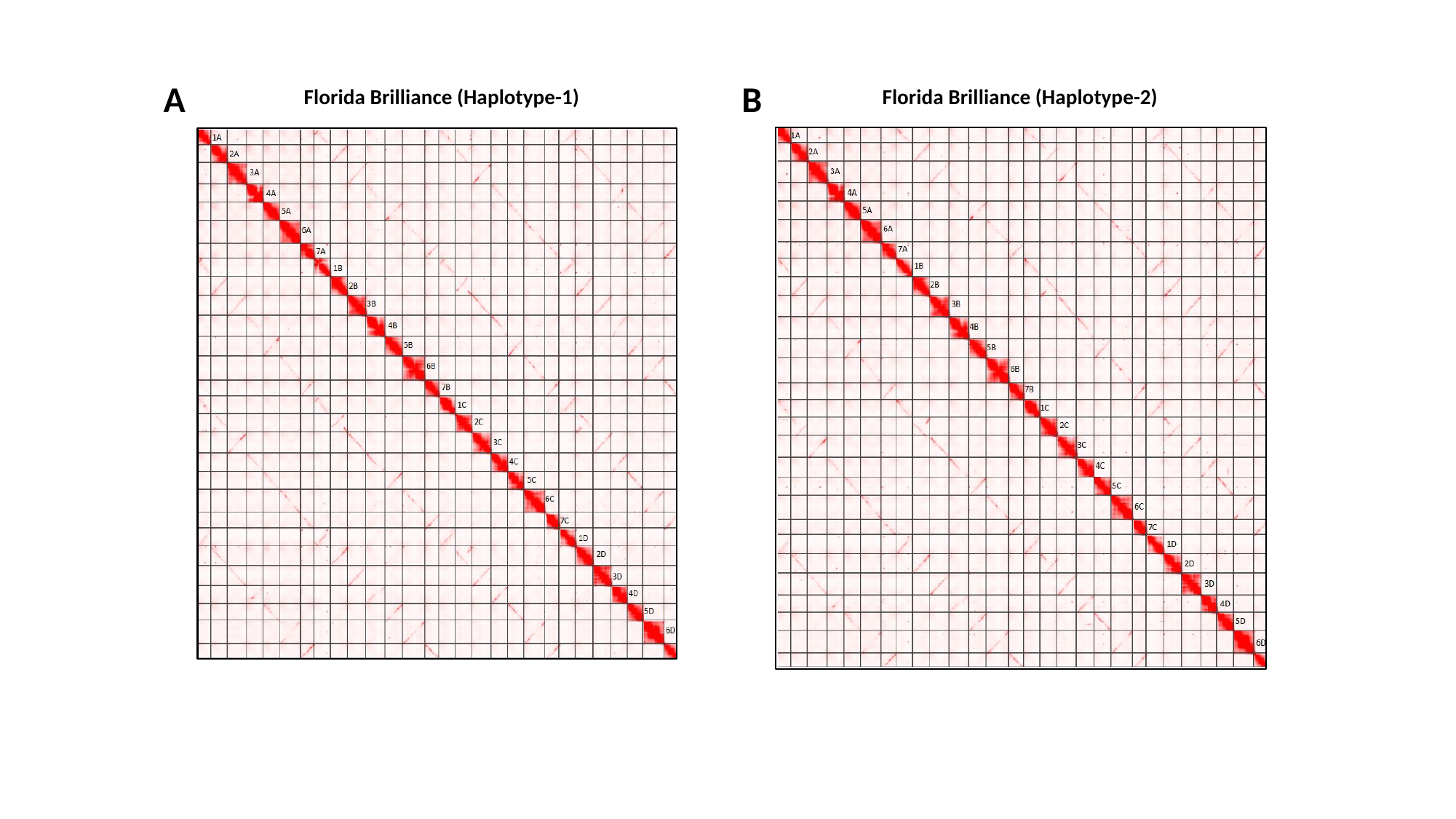

A
Florida Brilliance (Haplotype-1)
B
Florida Brilliance (Haplotype-2)

Supplement: giaf005_Supplemental_Files [file giaf005_supplemental_files.zip › Figure S6_Supplementary Material_Revised.pptx]

## Slide 1
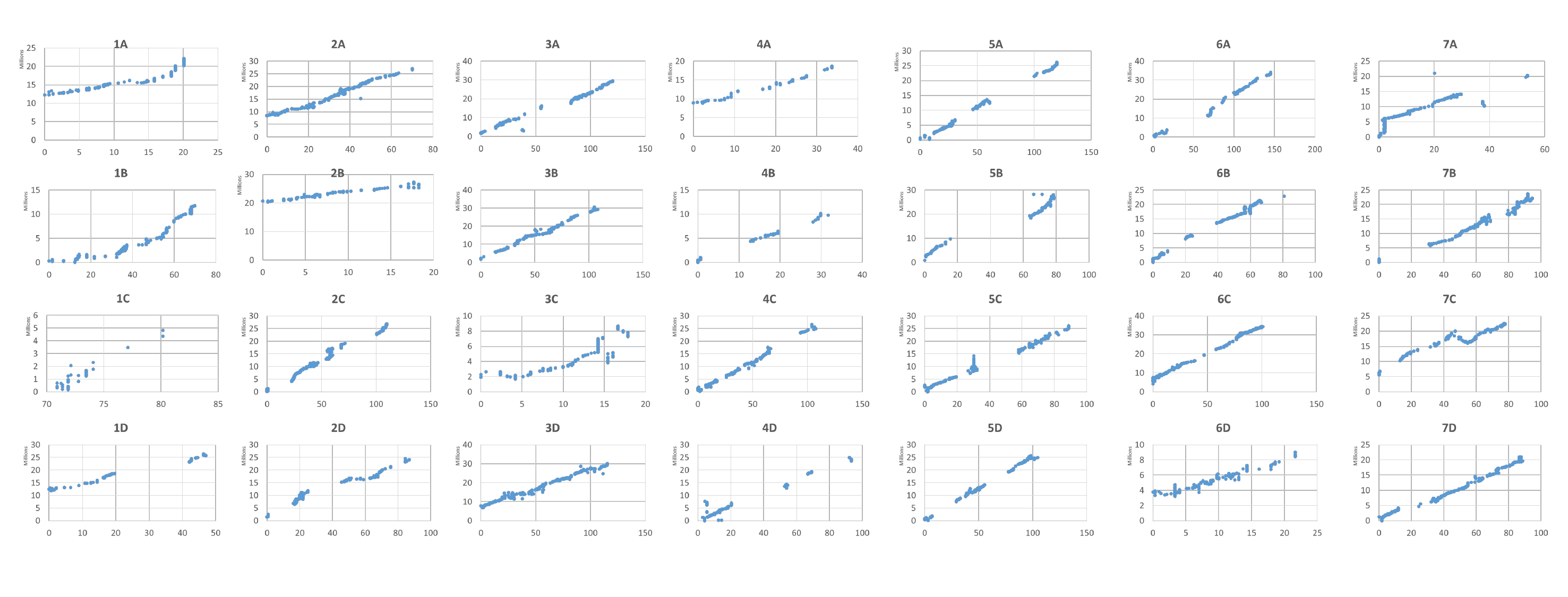

Supplement: giaf005_Supplemental_Files [file giaf005_supplemental_files.zip › Figure S7_Supplementary Material_Revised.pptx]

## Slide 1
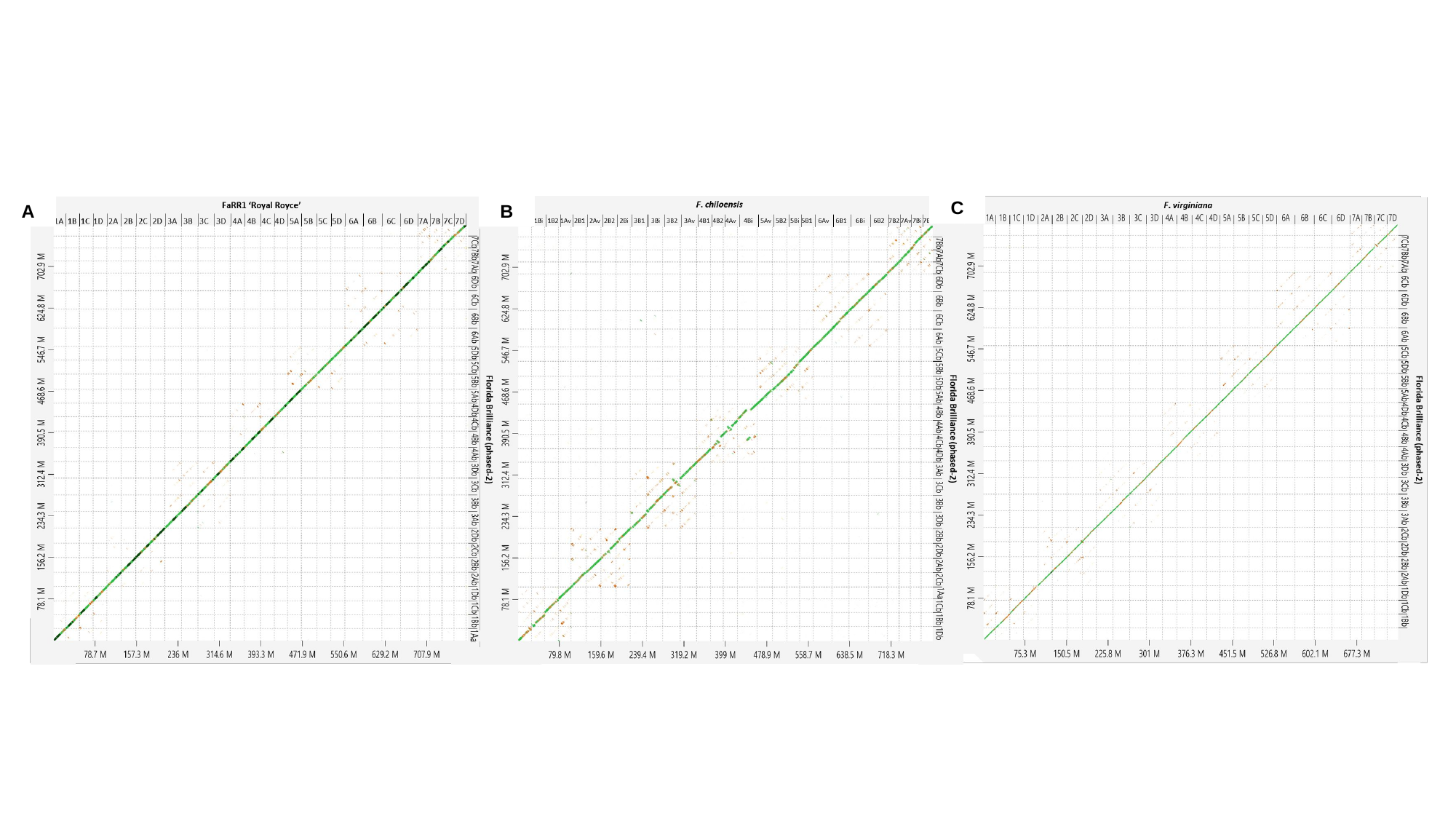

C
A
B

Supplement: giaf005_Supplemental_Files [file giaf005_supplemental_files.zip › Figure S9_Supplementary Material_Revised.pptx]
