# Supplementary material for: A telomere-to-telomere phased genome of an octoploid strawberry reveals a receptor kinase conferring anthracnose resistance
Source: Gigascience. 2025 Mar 12;14:giaf005. doi: 10.1093/gigascience/giaf005 (PMC11899574; doi:10.1093/gigascience/giaf005)
Supplement: giaf005_GIGA-D-24-00173_Original_Submission [file giaf005_giga-d-24-00173_original_submission.pdf]

GigaScience

A Telomere-To-Telomere Phased Genome of an Octoploid Strawberry Reveals a Receptor Kinase Conferring Anthracnose Resistance

--Manuscript Draft--

**Manuscript Number:**	GIGA-D-24-00173
**Full Title:**	A Telomere-To-Telomere Phased Genome of an Octoploid Strawberry Reveals a Receptor Kinase Conferring Anthracnose Resistance
**Article Type:**	Research
**Funding Information:**	National Institute of Food and Agriculture (2022-51181-38328) — Dr. Seonghee Lee
**Abstract:**	Backgrounds<br><br>Cultivated strawberry (Fragaria×ananassa Duch.), an allo-octoploid species arising from at least three diploid progenitors, poses a challenge for genomic analysis due to its high levels of heterozygosity and the complex nature of its ploidy genome.<br><br>Results<br><br>This study developed the complete haplotype-phased genome sequence from a short-day strawberry, 'Florida Brilliance' without parental data, assembling 56 chromosomes from telomere-to-telomere. This assembly was achieved with high-fidelity long reads and high-throughput chromatic capture sequencing (Hi-C). The centromere core regions and 96,104 genes were annotated using long-read isoform RNA sequencing. Using the high-quality of haplotype-phased reference genome, FaFB1, we identified the causal mutation within the gene encoding Leaf Rust 10 Disease-Resistance Locus Receptor-like Protein Kinase (LRK10) that confers resistance to anthracnose fruit rot (AFR), which is caused by Colletotrichum acutatum and significant economic losses in strawberry production. Comparison of resistant and susceptible haplotype assemblies and full-length transcript data revealed a 29-bp insertion at the first exon of the susceptible allele leading to a premature stop codon and the loss of gene function of LRK10. The functional role of LRK10 in resistance to AFR was validated using the simplified Agrobacterium-based transformation method for transient gene expression analysis in fruits of strawberry. Our results of transient knock-down and overexpression of LRK10 in fruit indicate the key role for LRK10 in AFR resistance in strawberry.<br><br>Conclusions<br><br>The FaFB1 assembly along with other resources will be valuable for the discovery of additional candidate genes associated with disease resistance and fruit quality, which will not only advance our understanding of genes and their functions but also facilitate advancements in genome editing in strawberry.
**Corresponding Author:**	Seonghee Lee<br>University of Florida<br>Wimauma, Florida UNITED STATES
**Corresponding Author Secondary Information:**	
**Corresponding Author's Institution:**	University of Florida
**Corresponding Author's Secondary Institution:**	
**First Author:**	HYEONDAE HAN
**First Author Secondary Information:**	
**Order of Authors:**	HYEONDAE HAN
	Natalia Salinas

Powered by Editorial Manager® and ProduXion Manager® from Aries Systems Corporation

	Christopher R Barbey
	Yoon Jeong Jang
	Zhen Fan
	Sujeet Verma
	Vance M. Whitaker
	Seonghee Lee
**Order of Authors Secondary Information:**	
**Additional Information:**	
**Question**	**Response**
Are you submitting this manuscript to a special series or article collection?	No
**Experimental design and statistics**<br><br>Full details of the experimental design and statistical methods used should be given in the Methods section, as detailed in our Minimum Standards Reporting Checklist. Information essential to interpreting the data presented should be made available in the figure legends.<br><br>Have you included all the information requested in your manuscript?	Yes
**Resources**<br><br>A description of all resources used, including antibodies, cell lines, animals and software tools, with enough information to allow them to be uniquely identified, should be included in the Methods section. Authors are strongly encouraged to cite Research Resource Identifiers (RRIDs) for antibodies, model organisms and tools, where possible.<br><br>Have you included the information requested as detailed in our Minimum Standards Reporting Checklist?	Yes
**Availability of data and materials**<br><br>All datasets and code on which the	Yes

conclusions of the paper rely must be either included in your submission or deposited in publicly available repositories (where available and ethically appropriate), referencing such data using a unique identifier in the references and in the "Availability of Data and Materials" section of your manuscript.<br><br>Have you have met the above requirement as detailed in our Minimum Standards Reporting Checklist?	

Powered by Editorial Manager® and ProduXion Manager® from Aries Systems Corporation

Manuscript Click here to access/download;Manuscript;Manuscript_vf.docx

A Telomere-To-Telomere Phased Genome of an Octoploid Strawberry Reveals a Receptor Kinase Conferring Anthracnose Resistance

Hyeondae Han[1#], Natalia Salinas[1#], Christopher R Barbey[1], Yoon Jeong Jang[1,2], Zhen Fan[1], Sujeet Verma[1], Vance M. Whitaker[1] and Seonghee Lee[1*]

[1]Department of Horticultural Sciences, University of Florida, IFAS Gulf Coast Research and Education Center, 14625 CR 672, Wimauma, FL, 33598, USA

[2]Department of Plant Science and Technology, Chung-Ang University, Anseong, 17546, Republic of Korea

[#] Equal contributions to this manuscript

[*] Correspondence: Seonghee Lee, IFAS Gulf Coast Research and Education Center, 14625 CR 672, Wimauma, FL, 33598, USA. e-mail: seonghee105@ufl.edu

Abstract

Backgrounds: Cultivated strawberry (*Fragaria ×ananassa* Duch.), an allo-octoploid species arising from at least three diploid progenitors, poses a challenge for genomic analysis due to its high levels of heterozygosity and the complex nature of its ploidy genome.

Results: This study developed the complete haplotype-phased genome sequence from a short-day strawberry, 'Florida Brilliance' without parental data, assembling 56 chromosomes from telomere-to-telomere. This assembly was achieved with high-fidelity long reads and high-throughput chromatic capture sequencing (Hi-C). The centromere core regions and 96,104 genes were annotated using long-read isoform RNA sequencing. Using the high-quality of haplotype-phased reference genome, FaFB1, we identified the causal mutation within the gene encoding *Leaf Rust 10 Disease-Resistance Locus Receptor-like Protein Kinase* (*LRK10*) that confers resistance to anthracnose fruit rot (AFR), which is caused by *Colletotrichum acutatum* and significant economic losses in strawberry production. Comparison of resistant and susceptible haplotype assemblies and full-length transcript data revealed a 29-bp insertion at the first exon of

the susceptible allele leading to a premature stop codon and the loss of gene function of *LRK10*. The functional role of *LRK10* in resistance to AFR was validated using the simplified *Agrobacterium*-based transformation method for transient gene expression analysis in fruits of strawberry. Our results of transient knock-down and overexpression of *LRK10* in fruit indicate the key role for *LRK10* in AFR resistance in strawberry.

Conclusions: The FaFB1 assembly along with other resources will be valuable for the discovery of additional candidate genes associated with disease resistance and fruit quality, which will not only advance our understanding of genes and their functions but also facilitate advancements in genome editing in strawberry.

Keywords

Anthracnose, fruit rot, root necrosis, *Colletotrichum acutatum*, *FaRCa1*,

Background

Cultivated strawberry (*Fragaria* × *ananassa* Duch.) is an allo-octoploid species (2n = 8x = 56) that originated from spontaneous interspecific hybrids of two wild octoploid species *F. chiloensis* Duch. and *F. virginiana* Duch [1]. Moreover, strawberry is the most widely distributed fruit crop in the world and serves as a model species for the *Rosaceae* family which includes 27 fruit crops. Until recently, sequencing technologies were not sufficiently advanced to produce highly accurate assemblies of complex genomes. A complete and accurate sequence for both alleles at any given genomic region is a powerful resource for functional genomics research. The first chromosome-scale genome assembly for octoploid strawberry 'Camarosa' [2] contributed to an examination of the evolutionary history of the octoploid strawberry, its domestication and studies of disease resistance and fruit quality [3-5]. It is proposed that the octoploid progenitors of cultivated strawberry emerged through consecutive stages of polyploidization of at least three diploid progenitor species over a million years ago, with the major diploid progenitors identified as *F. vesca* and *F. iinumae* [2, 6]. A subgenome nomenclature has been proposed to assign the 28 homoeologous chromosomes to subgenome A, B, C, and D based on whether four diploid strawberry *F. vesca, F. innumae, F. nipponica*, and *F. viridis* transcripts accounted for the largest remaining fraction of gene sequences [4]. Because of

its complex genome, functional genomics for octoploid strawberry have lagged compared to diploids. Yet the commercial importance of cultivated strawberry highlights a need for high quality genomes to drive selective breeding and gene editing applications.

The reference genome assembly is a fundamental platform to investigate the biological features of organisms. To produce a genome assembly, parental haplotypes are often collapsed to consensus sequences, referred to as a haplotype-merged assembly. The haplotype-merged assembly of a highly heterozygous and polyploidy genome means loss of genetic variation from one of the two haplotypes derived from both parents, resulting in a limited view of genetic diversity. In this context, the haplotype-phased genome assembly is of great importance to thoroughly understand the genetic variation that characterizes the genome. Recently, using high-fidelity (HiFi) long-read sequencing, the haplotype-phased genome of the day-neutral strawberry 'Royal Royce' was reported by applying the trio-binning pipeline with parental short reads [7]. In this study, the fully haplotype-phased genome from a short-day strawberry 'Florida Brilliance', was completed from telomere-to-telomere (T2T) without parental data by combining PacBio HiFi reads, Hi-C chromatin interaction data and full-length transcriptome sequencing (Isoform sequencing) [8, 9]. Long-read sequencing technology combined with computational algorithms has not only contributed to completing the T2T human genome, but also contributed to completing the T2T genome of major plant species such as corn [10], banana [11], and diploid strawberry [12]. The studies on the T2T genome for octoploid strawberry have not been described.

High-quality assemblies of highly heterozygous polyploid genomes are powerful for investing structural variations and identifying candidate genes associated with plant traits. The genome of 'Florida Brilliance', FaFB1, has particular value as this cultivar is widely grown and used in breeding and thus possesses economically important gene-trait mechanisms. For instance, a major disease resistance locus, *FaRCa1*, was recently identified in strawberry that confers strong resistance to anthracnose fruit rot (AFR) explaining at least 50% of the phenotypic variation across trials and moderate resistance to anthracnose root necrosis (ARN) caused by the *Colletotrichum acutatum* species complex [13, 14]. The SNP marker of *FaRCa1* conferring resistance to AFR also conferred a significant reduction in ARN. Previous European research demonstrated that a dominant allele at the single locus *Rca2* controls high-level

resistance to *C. acutatum* isolate 688b from pathogenicity group (PG) 2 in strawberry [15]. *Rca2* is present in European cultivars Mamie, Gariguette and Belrubi and in U.S. cultivars Sequoia, Dover, and US15. *Rca2* is also present in two genotypes of *F. vesca* and one of *F. moschata* which have shown resistance phenotype. Resistance to *C. acutatum* has also been observed in *F. chiloensis* and *F. virginiana*. It was also reported that minor genes control intermediate levels of resistance in cultivar Addie. Disease response was scored on the whole plant according to the scale from 0 (no visual symptoms) to 5 (death plant). Five other QTLs were found to give moderate resistance to the *C. acutatum* isolate 494a, which was assigned to PG-1, in a cross between 'Capitola' and CF111664. Disease response was recorded on vegetative tissue of the whole plant using the scale from 0 (no visual symptoms) to 5 (death plant). Since the *Rca2* resistance allele was reportedly present in UF germplasm, it was originally assumed that *Rca2* was the main resistance source in the UF breeding population. However, molecular markers linked to *Rca2*, which give resistance to *C. acutatum* isolate 688b and 1267b from PG-263, do not explain resistance/susceptibility to AFR in UF cultivars. UF cultivars possessing *FaRCa1* are a source for AFR resistance in other breeding programs worldwide.

One of the major challenges in strawberry genetics has been the availability of high-throughput, subgenome-specific markers. Since the cultivated strawberry has four subgenomes, each locus can represent up to eight different alleles in a single individual, making it difficult to accurately analyze segregation. Recently, the strawberry subgenome diversity was estimated based on whole-genome shotgun (WGS) sequencing of 93 genealogically and phylogenetically diverse *F.* × *ananassa*, *F. chiloensis*, and *F. virginiana* individuals [16]. Subgenome-specific polymorphisms that were able to distinguish homologous from homoeologous DNA sequences on every chromosome were used to develop 850K and 50K SNP arrays [16]. These arrays now provide an abundance of subgenome-specific SNPs for use in genetic studies in strawberries. The robust and easily scored DNA test is now available to breeders for selecting for resistance to both the fruit and root forms of strawberry anthracnose. While this locus has been important in breeding, the genomic structure and candidate genes for AFR resistance have not yet been characterized [14].

Plants use a diverse group of receptors located in the cell membrane that can perceive signals from exterior such as changes in temperature, light, nutrition and presence of pathogens,

and induce adequate responses to ensure plant survival. These receptors can be classified in two main groups; first, the nucleotide-binding site leucine-rich repeat (NBS-LRR) receptors and histidine kinase receptors, and second, the receptor protein kinases (RPK). RPK in plants are grouped into two main major subclasses based on their substrate specificity. The first subclass comprises serine/threonine kinases (STK) that phosphorylate serine and threonine residues, and the second subclass consists of receptor histidine kinases (RHK) that phosphorylate histidine residues [17]. Most of RPKs in plants share a common structure that includes predicted extracellular signal sequence, an LRR, a single pass transmembrane helix, and a cytoplasmic kinase domain with the serine/threonine consensus sequence. RLKs have been described as resistant genes that initiate disease response to specific pathogens, but also RLKs can play an important role in innate immunity [18]. A gene encoding Leaf Rust 10 Disease-Resistance Locus Receptor-like Protein Kinase (LRK10) identified as a candidate gene for AFR resistance in this study is a member of the RLK family, and mostly closely related to wheat LRK10 associated with disease resistance. In Arabidopsis, LRK10 is involved in ABA-mediated signaling and drought resistance [19].

'Florida Brilliance' is the main short-day cultivar commercially grown in Florida and is heterozygous (*Ca1ca1*) at the *FaRCa1* locus and resistant to ARN and ARN. In this study, we describe a telomere-to-telomere haplotype-phased genome of 'Florida Brilliance' (FaFB1). A complete and accurate sequence for both alleles at any given genomic region is a powerful resource for functional genomics research. The highly accurate sequences of both alleles led to the discovery of causal mutation and functional validation of a major resistance gene LRK10 in AFR disease resistance. Furthermore, our findings highlight its value to the strawberry research community via the functional characterization of *FaRCa1*.

Results and Discussion

Telomere-To-Telomere Haplotype-Phased Assembly of the Octoploid Strawberry Genome

The high-fidelity (HiFi) long reads (Pacific Biosciences of California) and Hi-C chromatin interaction data (Dovetail) were used to assemble the short-day variety of 'Florida Brilliance'

octoploid strawberry genome (Table S1, Figure S1). Using five single-molecular real-time cells in PacBio Sequel II platform, 144.1 Gb of sequences were generated in 9.1M reads. The average read length was 15,834.8 bp. The Hi-C data was generated on Novaseq6000 and contained 86.5 Gb of sequences in 286 M paired-end reads with an average length of 151 bp. The combined power of these data produced a draft assembly with an N50 of 23.7 Mb for haplotype-1(H-1) and 26.7 Mb for haplotype-2 (H-2), indicating that on average one contig corresponded to a single chromosome. This compares to N50 for 'Camarosa' of 3.9 Mb and 'Royal Royce' of 11 Mb [20]. Before scaffolding, the Benchmarking Universal Single-Copy Orthologs (BUSCO) scores were 99.2% in the H-1 assembly and 99.1% in H-2 assembly. When comparing the full assembly with the HiFi reads of FaFB1 using Merqury, the results revealed a high base accuracy (QV>69.8), indicating that 99.99% of HiFi reads were found in the combined assembly. The final assembly contained 99.1% complete gene models with a majority (96.6%) of the duplicated complete gene models in both H-1 and H-2 genome assemblies (Table 1). Chromosome names in the 'Florida Brilliance' genome assemblies followed the nomenclature of proposed by Hardigan, Feldmann [20] and used in FaRR1, reflecting the proposed diploid origins of each subgenome (A, B, C, and D) [20].

Table 1. Statistics of the 'Florida Brilliance' genome assembly and annotation.

**Assembly Metrics**	**Value**	
	**Haplotype-1**	**Haplotype-2**
**Draft assembly**		
Number of contigs	3,716	1,226
Number of contigs (≥ 50 kb)	750	618
Length of largest contig (Mb)	34.6	34.8
Assembled genome size (Mb)	866	839.13
GC content (%)	40.67	39.84
Length of contig N50 (Mb)	23.72	26.65
Length of contig N75 (Mb)	12.38	17.30
L50	18	15
L75	31	24

BUSCO (%)	99.2	99.1
Single	2.6	2.5
Duplicated	96.6	96.6
Fragmented	0	0.1
Missing	0.8	0.8
Base accuracy (QV by Merqury)	69.9	68.5
**Final assembly**		
Assembled genome size (Mb)	784.9	781.0
Number of anchored contigs	137	86
BUSCO (%)	99.1	99.1
Single	2.5	2.3
Duplicated	96.6	96.8
Fragmented	0	0.1
Missing	0.9	0.8

Since the first report of a chromosome-scale reference genome [2], several octoploid strawberry (*F.* × *ananassa*) reference genomes using HiFi reads have been generated [2, 7, 21-23]. The high accuracy of HiFi reads notably improved the assembly quality for octoploid strawberry. The N50 of 'Florida Brilliance' initial H-1 and H-2 assemblies were 23.7 Mb and 26.7 Mb and comparable to other genomes: Wongyo3115 (9.84 Mb), 'Royal Royce' (11 Mb) [7], '15.89-25' [24], 'Yanli'(27.5 Mb) [23], *F. chiloensis* (10.8 Mb) [25], and 'Reikou' (3.9 Mb) [22]. The final assembly consisted of 784.9 Mb for H-1 and 781.0 Mb for H-2 which are similar to 'Royal Royce' (784 Mb) [7] and other genomes [21, 23, 24]. When comparing the corresponding chromosome lengths between 'Florida Brilliance' and 'Royal Royce', we found high similarity between 'Royal Royce' and 'Florida Brilliance' (Figure S2). Overall, these results suggest that high quality octoploid strawberry genomes can be successfully generated with and without parental data [8].

Hi-C reads were re-analyzed to complete scaffolding the H-1 and H-2 assemblies. Among 286 M of Hi-C read pairs, approximately 92% of Hi-C reads were mapped to the assembly (Table S2). The smudgeplot displays the genome structure of cultivated strawberry based on *k*-mer analysis, supporting allo-octoploid genome structure (Figure S3). Unique Hi-C

read pairs were visualized via a Hi-C contact map of 28 chromosomes in each of the H-1 and H-2 assemblies (Figure S4). The Hi-C read pairs were evenly distributed, indicating a low probability of misassembly. There was high collinearity between the H-1 and H-2 assemblies of 'Florida Brilliance' (Figure 1A). Alignment of the H-2 assembly against the diploid *F. vesca* v4.0 showed a high degree of collinearity except for major translocations on 1A and 2C (Figure 1B). As expected, 'Florida Brilliance' sub-genome A showed clearly higher sequence similarity with *F. vesca* than other three sub-genomes. From each pair of pseudo-chromosomes (56 total) in the combined assembly, we selected the most continuous pseudomolecule to produce a final FaFB1 assembly. Genetic positions and physical coordinates of markers were collinear with a high degree of agreement between FaFB1 and the linkage map (Figure S5). Collinear gene pairs between 'Florida Brilliance' and *F. vesca* were identified and were consistent with collinearity calculated by D-Genies (Figure S6).

Telomeres, basic structure of eukaryotic chromosomes, are typically tandemly arranged mini-satellites following the formula (TxAyGz)n at the both ends of chromosomes [26], the putative telomeric sequences (5'-TTTAGGG-3') of octoploid strawberry were first described at both ends of seven out of the 28 pseudo-chromosomes of *F. chiloensis* [25]. Here, we identified 103 out of a possible 112 telomeres across the two haplotypes. Putative telomeric sequences were found at or near the 5' and/or 3' ends in all 56 pseudo-chromosomes (Figure 2). However, chromosome 7Ba and 7Bb contained putative telomeric sequences located 6 Mb or 7 Mb from one end. In addition, seven chromosomes (1Aa, 1Ab, 1Bb, 2Ba, 2Bb, 6Da, and 6Db) had a short interstitial telomere-like sequences (for example, ~10 repeats rather than ~100) which could be due to misassemblies of these repetitive regions. Gene distribution along the chromosomes followed the typical distribution of monocentric plant genomes, and the positions of centromeres were similar between the two haplotypes (Figure 2). To evaluate both haplotype assemblies based on core eudicots genes, genome quality was assessed using the LTR assembly Index (LAI) [27]. LAI for each chromosome ranged from 17.17 to 20.13. LAI for combined genome was 19.72 (Table S3), comparable to 'Royal Royce' and *F. vesca* Hawaii 4 [7]. In the combined assembly there were 652 Mb of repetitive sequence accounting for 41.66% of the genome, which was higher than other published references including 'Royal Royce' (38.4%), 'Camarosa' (36%) and 'Wongyo 3115' (38.75%)[21]. Most of these repeat sequences were composed by LTR class transposable

elements (Table S4). Among them, 223,060 simple sequence repeats (SSRs) representing 0.16% of whole genome sequence were found (Table S5).

Annotation of Gene Content at the Subgenome Level of Octoploid Strawberry

To facilitate annotation, we generated a full-length transcriptome, including isoforms, of 'Florida Brilliance' using long-read Iso-Seq for leaf, root, flower, green fruit, and red fruit samples. Additionally, Iso-Seq data for 15 'Royal Royce' tissues or treatments was included for gene prediction [7]. RNA-Seq data representing *F.* × *ananassa* tissues including various tissues including green achene, red achene, runner, turning achene were also downloaded from NCBI sequence read archive. We obtained a set of 320,979 transcripts by aligning Iso-Seq and RNA-Seq datasets to the combined H-1 and H-2 assemblies. BUSCO analysis of the transcript assemblies revealed 2,209 complete core embryophyta genes (97.5%, 2.9% single-copy, 94.6% duplicated) with 1.1% fragmented and 1.4% missing core eudicots genes (Table S6). Integrating *ab initio* predictions and evidence-based (RNA-Seq and Iso-Seq) prediction, 254,623 total gene models were predicted, roughly 127,000 models per haploid genome. After excluding TE-related gene models by two criteria, 202,456 genes remained in the final annotation with 100,850 models in the H-1 assembly, 101,606 models in the H-2 assembly, and 101,723 in the FaFB1 assembly (Tables S7 and S8), similar to 'Royal Royce' (101,721) [7] and 'Camarosa' (108,087) [2] and quite different from 'Wongyo3115' (151,892) [21] and 'Reikou' (167,721) [22]. When classifying all predicted genes by sub-genome, sub-genome A representing diploid progenitor, *F. vesca*, accounted for 27.5% of genes, which is similar to 'Royal Royce' (27%) [7]. We observed biased gene distribution among the sub-genomes of octoploid strawberry (A vs B, df = 6, P = 0.007; A vs C df = 6, P = 0.006; A vs D; df = 6, P = 0.0002), while the number of genes in sub-genomes B, C, and D were not significantly different. This suggests a lower rate of gene loss in sub-genome A (Figure 4), which was also observed in the 'Royal Royce' genome (Table S9 and S10).

Haplotype Alignment and Candidate Gene Detection Associated with the Resistance Locus *FaRCa1*

Previous studies have extensively documented the wide-ranging resistance variability to AFR within the University of Florida (UF) breeding germplasm, with the majority of cultivars exhibiting moderate to high resistance levels [29-31]. Within UF germplasm, resistance to AFR is primarily governed by a single locus, *FaRCa1* [13, 14]. This locus has been shown to account for a minimum of 50% of the phenotypic variance in AFR incidence across two distinct quantitative trait locus (QTL) discovery populations and has been corroborated in advanced selections and cultivars[13]. Utilizing four single-nucleotide polymorphisms (SNPs) from the Axiom IStraw35 array[32], the *FaRCa1* region was delineated, with probe AX-89838986 demonstrating the highest explanatory power across both discovery and validation populations[13]. Subsequently, the 9-bp InDel-based marker located in *FaRCa1* was developed for the marker-assisted selection of the trait [14]. So far, no additional loci were detected, indicating that *FaRCa1* singularly confers the observed resistance in UF germplasm, as opposed to *Rca2,* which imparts resistance to certain strains of *C. acutatum* in Europe [15].

Resistant and susceptible haplotype assemblies for the previously described major resistance locus, *FaRCa1* (13, 14), were delimited between the 50K FanaSNP Array probes 'AX-89896208' and 'AX-89838962' in Chr 6B of 'Florida Brilliance' genome (FaFB1) (Figure 4A). The *FaRCa1* region was defined to 136 kb in the H-1 assembly (Resistant-*FaRCa1*; Chr. 6Ba) containing 13 genes and 158 kb in the H-2 assembly (Susceptible-*farca1*: Chr. 6Bb) containing 22 genes (Figure S8A and Table S11). The resistant *FaRCa1* haplotype showed six locally collinear blocks (LCBs) when compared to the homoeologous *farca1* regions of chromosomes 6A and 6D (Figure S8A). In contrast, the homoeologous *farca1* haplotype on chromosomes 6A and 6D was 300 kb longer than the resistant haplotype and comprised more than 10 locally collinear blocks LCBs (Figure S8A). The susceptible haplotype of chromosome 6Bb showed three LCBs when aligned with the homologous genomic regions of 'Royal Royce' (Figure S8B), suggesting that this variety carries two susceptible alleles of *farca1*. This was expected as none of the previously assembled strawberry genomes was of a genotype known to carry this resistance locus.

The thirteen annotated genes in the region of *FaRCa1* had diverse predicted biological functions (Table S11). Only two genes, Fxa6Bg1786910 (named *LRK10A*) and Fxa6Bg1786920 (named *LRK10B*), were related to plant defense responses – functionally predicted as '*Leaf Rust 10 Disease-Resistance Locus Receptor-Like Protein Kinase*' in Arabidopsis. These two *LRK10*

genes, *LRK10A* and *LRK10B*, are located closely to each other. These two annotated genes are located 20 kb and 44.6 kb from the 9-bp InDel DNA marker that has been used for a high-throughput marker assisted seedling selection [33]. The full-length transcript data from 'Florida Brilliance' was used to confirm the sequence and structural variations of genes located in the *FaRCa1* region. Notable sequence variations were found at Fxa6Bg1786910 (*LRK10A*). Sequence alignments between resistant (Fxa6Bg1786910 – *LRK10A*) and susceptible (Fxa6Bg2100330 – *LRK10a*) alleles showed a 26-bp deletion in the first coding region and eight SNPs within the remaining coding sequence of the susceptible allele (Figure 5B). This deletion introduces a premature stop codon that disrupts the downstream protein-coding sequence (Figure 5C). The alignment of *LRK10B* (Fxa6Bg1786920 at Chr. 6Ba) between resistant and susceptible haplotypes revealed 99.3% (2082/2097) of sequence similarity (Figure 5C). The Iso-seq data of 'Florida Brilliance' showed that the full-length transcript of LRK10A is expressed in fruit tissue only, but LRK10B expression was not detected in any tissues of Iso-seq data. This finding suggests that *LRK10A* appears to have an important role in resistance to *Colletotrichum acutatum* in strawberry. The wheat leaf rust kinase (*wlrk*) *RLK* gene family that encodes receptors involved in pathogen recognition are also constitutively expressed in the aerial parts, whereas no expression was detected in the roots [34].

LRK10A conforms to the typical features of RLK: a variable ectodomain for ligand binding, a single-pass transmembrane domain, and a cytoplasmic kinase domain (Figure 5) [35]. Because the ability of plants' receptor-like kinase to detect extracellular stimuli and transmit signals across the plasma membrane (PM) is vital for their defense against pathogens, *LRK10A* localized on the PM are expected to be responsible for detecting conserved microbe-associated molecular patterns (MAMPs) or damage-associated molecular patterns (DAMPs), initiating a cascade of immune responses [36]. The main biological and molecular functions of this protein are phosphorylation, protein kinase activity, and ATP and polysaccharide binding according to the InterPro 87.0 blast results. Leaf rust kinase (LRK) 10-like proteins belong to the receptor-like kinases (RLK) family which is a subclass of the serine/threonine kinases (STK), a major subclass of the receptor protein kinases (RPK)[17] [18]. RPK are receptors located in the cell membrane that can perceive changes from the plant exterior including temperature, light, nutrition and the invasion of pathogens.

The *Lr10* gene controls resistance to leaf rust caused by fungus Puccinia recondite in wheat [37]. *Lr10* was first identified in hexaploid wheat, and it is located on chromosome 1AS. *Lr10* has a length of 3,935 bp and contains an intron of 1,171 bp [37]. *Lr10* encodes the protein LRK10 which is composed by coiled coil (CC), nucleotide-binding site (NBS), leucine-rich repeat (LRR) (CNL) protein of 919 amino acids and 14 imperfect LRR at the C terminus. The primary structure of LRK10 proteins includes a signal peptide, a cysteine-rich extracellular domain, a transmembrane domain, and a predicted intracellular serine/threonine kinase domain. In a broad sense, this structure is similar to the structure described for the 670-amino acid protein encoded by LRK10A in strawberry. When wheat plants were overexpressed with Lr10, resistance to leaf rust was improved [38].

A Receptor-Like Kinase, *LRK10*, Contributing to the Resistance to Anthracnose Fruit Rot

LRK10A encodes a 670-amino acid protein, while the truncated susceptible allele consists of only 53-amino acids, resulting in the loss of the functional kinase domain (Figure 5C). LRK10A is composed of six predicted regions: signal-peptide N-region (1-5 aa), signal peptide H-region (6-17 aa), signal peptide C-region (18-25 aa), non-cytoplasmic domain (26-290 aa), transmembrane region (291-314 aa), and cytoplasmic domain (315-670 aa). The protein kinase domain can be found between acid 352 and 640. Along the cytoplasmic domain two sites can be distinguished: the protein kinase ATP binding site (358 - 380 aa) and the serine/threonine kinase active site (472 - 484 aa) according to the InterPro 87.0 database results. The main biological function of LRK10 is phosphorylation, and the main molecular functions are protein kinase activity and ATP and polysaccharide binding (InterPro 87.0). For LRK10B, there are only three differing amino acids in this gene between resistant and susceptible alleles, which do not alter the functional conserved domain (Figure 5E). The expression of *LRK10A* and *LRK10B* in strawberry fruit showed significantly different expression patterns among the homozygous susceptible (*ca1ca1*), heterozygous (*Ca1ca1*), and homozygous resistant (*Ca1Ca1*) genotypes. Relative expression of LRK10A after *C. acutatum* inoculation decreased through time in homozygous susceptible (*ca1ca1*) genotype, 16.74-68. Relative expression of LRK10A in homozygous resistant *Ca1Ca1* and heterozygous resistant *Ca1ca1* genotype increased after *C. acutatum* inoculation and remained at high levels at 24, 48, 72 and 96 hpi (Figure S9). However, relative

expression of gene LRK10B after *C. acutatum* inoculation was reduced over time in susceptible genotype *ca1ca1* and homozygous resistant genotype (*Ca1Ca1*). For the heterozygous resistant (*Ca1ca1*) 'Florida Brilliance', the relative expression of gene LRK10B seemed relatively similar at 24, 48, 72 and 96 hpi. This finding indicates the involvement of LRK10A in the resistance against the pathogen.

To further validate the functional role of these two genes against the *C. acutatum* species complex, we applied robust and efficient *Agrobacterium*-based transformation methods for transient gene expression analysis in fruits of strawberry. RNAi constructs targeting both *LRK10A* and *LRK10B* were designed (Table S12). *A. tumefaciens* EH105 containing RNAi constructs targeting each gene, LRK10A: RNAi and LRK10B: RNAi, was injected in green fruits of homozygous resistant (*Ca1Ca1*) 17.20-51, heterozygous resistant (*Ca1ca1*) 'Florida Brilliance', and homozygous susceptible (*ca1ca1*) 16.74-68. *A. tumefaciens* containing empty vectors was also used as control in each genotype. The mRNA level was reduced by a maximum of approximately 35% in *LRK10A* and *LRK10B*-knockdown individuals compared to the empty vector control (Figure S10A). The internal symptom area of the *LRK10A*-knockdown individuals increased significantly in resistant genotypes (homozygous - *Ca1Ca1* and heterozygous - *Ca1ca1*) at 16 days after inoculation (DAI) (Figure 6A and B). In contrast, *LRK10B*-knockdown individuals showed no significant difference in internal symptom area (Figure S10B). In the transient overexpression assay, the internal symptom area of all genotypes in *LRK10A*-overexpressed fruit was significantly reduced compared to the empty vector control fruit (Figure 6C and D). The expression level of *LRK10A* in the overexpression fruit was three to six times higher than that in the empty vector control, respectively (Figure S11). These findings indicate that *LRK10A* is a key genetic factor regulating the *FaRCa1*-mediated resistance to *C. acutatum*.

Conclusion

In conclusion, we have described a high-quality T2T, haplotype-phased genome of octoploid strawberry assembled without parental information. The accuracy and completeness of this reference genome allows for the precise identification of heterozygous alleles associated with specific traits. Leveraging this haplotype-phased genome alongside comprehensive full-length transcriptome data led to the identification of the candidate gene, *LRK10A*, by comparing the

genomic and transcriptomic sequences of resistant and susceptible alleles in the heterozygous ‘Florida Brilliance’. The *Agrobacterium*-mediated transient knockdown and overexpression experiments in strawberry fruit provided consistent functional evidence for a role in *FaRCa1*-mediated resistance against the *C. acutatum* complex in strawberry. This resource, along with other complete strawberry genomes, will continue to be a powerful tool for discovering gene to trait mechanisms in cultivated strawberry.

Methods

Plant materials and long read sequencing

Etiolated leaf tissues of ‘Florida Brilliance’ was used for genomic DNA extraction and library preparation at DNALink (Seoul, South Korea). The single-molecule real-time sequencing (SMRT) bell library was constructed using a PacBio DNA Template Prep Kit 1.0 (Pacific Biosciences, Menlo Park, CA, USA). Quality and quantity of each library was checked using a 2100 Bioanalyzer (Agilent Technologies). The SMRT Bell-Polymerase complex was constructed using a PacBio Binding Kit 2.0 (Pacific Biosciences) based on the manufacturer's instructions. The complex was loaded onto five SMRT cells (Pacific Biosciences, Sequel SMRT Cell 1M v2) and sequenced using a Sequel Sequencing Kit 2.1 (Pacific Biosciences, Sequel SMRT Cell 1M v2). For each SMRT cell, 1 × 600 min movies were captured using the Sequel sequencing platform (Pacific Biosciences) at DNALink (Seoul, South Korea). The quality of Hifi data was measured with LongQC [39].

For transcriptome sequencing, six ‘Florida Brilliance’ tissues were used for construction of PacBio Iso-Seq libraries (Table S1). About 10 grams of six tissues were collected from five plants: flower, green fruit, red fruit, crown, yleaf, and root. All tissues were flash frozen at -80℃ after washing with water. Total RNA was extracted using Spectrum™ Plant Total RNA Kit (Sigma-Aldrich, St. Louis, MO) according to manufacturer’s instructions. The extract was treated with DNAse I (Invitrogen) and re-suspended in a total volume of 50 µl of RNase-free water. The RNA samples were submitted to DNA Link for preparation of the Iso-Seq library and sequencing.

Among the Iso-seq sequences, when restricted to HiFi (QV≥20), 45,577 to 56,707 reads with an average length of about 2,500 bp were obtained from six tissues. In total, 12.1 Gb of 'Royal Royce' Iso-Seq sequence data [7] and 511 Gb of short read Read-seq were used as input for transcriptome assembly and gene annotation (Table S13).

De Novo genome assembly and validation

The genome characteristics including genome size and repetitive elements were estimated using PacBio HiFi data by K-mer spectrum distribution analysis for $k = 21$ in KMC3[40] and GENOMESCOPE v 2.0 [41]. The HiFi and Hi-C reads were used to produce a haplotype-phased assembly without sequencing of parents using Hifiasm [8]. Hifiasm was run with the following command according to developer's recommendation for heterozygous crops: hifiasm -o <outputPrefix> -t <nThreads> -D10 <Hifi-reads.fasta> --h1 <Hi-C_reads1> --h2 <Hi-C_reads2>. SALSA2, which is Hi-C-based scaffolding programs, was used for scaffolding contigs. Hi-C Reads were mapped via the Arima-HiC mapping pipeline (https://github.com/ArimaGenomics/mapping_pipeline). After mapping Hi-C reads to each phased genome assembly using BWA v0.7.17 [42] the sequence alignment map (SAM) format was converted to bed format using SAMtools [43] and BEDTools [44] prior to SALSA2 scaffolding (https://github.com/marbl/SALSA; [45], which was run with parameters -e GATC -m yes. Hi-C reads were mapped to chromosomes using HiC-Pro [46] in order to assess the quality of the assembly. The interaction matrix of whole chromosomes was visualized with heatmaps. Remaining contigs were scaffolded and oriented based on the '15.89-25' (https://www.rosaceae.org) reference genome using Ragtag [47]. A high-density genetic map was developed using a total of 169 F1 individuals from a cross between 'Florida Brilliance' and 16.33-8. Axiom™ 50K FanaSNP Genotyping Array were used to genotype all 169 F1 individuals. Markers were filtered to have <5% missing data and fit segregation ratios of 1:1 and 1:2:1 ($\alpha = 0.05$). Marker genotype calls were recoded to fit Joinmap 4.1 linkage mapping requirement. For example, markers with paternal segregation (AA × AB or BB × AB) coded as "nn × np"; markers with maternal segregation (AB × AA or AB × BB) coded as "lm × ll": and markers segregating in both parents (AB × AB) coded as "hk × hk." Mapping was conducted in an iterative process using the maximum likelihood algorithm in JoinMap 4.1 with default

settings. After each round of mapping, a graphical genotyping approach was applied to identify singletons to fix the marker order and regions with low marker density or gaps caused by segregation distortion. The genetic linkage map of 'Florida Brilliance' consisting of 10,269 SNP markers was used to validate the scaffolds from the FaFB1 whole genome assembly. SNP probes sequences used in the construction of linkage maps were mapped to the FaFB1 assembly sequence using blastn procedure [48]. Alignments were filtered to retain markers if they matched to unique sequence position in the FaFB1 phased genome assembly and with a maximum of 2 mismatches in the second best hit. The alignments were queried to detect problematic scaffolds mapped with SNP probes from different LGs. The number of scaffolds with SNP probes mapped from different LGs was used as a metric in the quality assessment of FaFB1 assembly.

Validation of assembly quality

Genome assembly statistics were calculated using QUAST version 5.0.266. Merqury version 1.3 were used to measure assembly consensus quality value (QV), evaluates assembly based on efficient K-mer set operations [49]. The completeness of the haploid assemblies and protein-coding gene annotations were assessed with the BUSCO database [50]. The scaffolds were inspected on the Hi-C contact map. Hi-C reads were trimmed with Homer [51] and mapped to both haploid assemblies using HiC-Pro version 3.0 [46] and visualized in Juicebox version 1.11 [52]. LAI [27] for each sub-genome was calculated using LTR-retriever [53] along with whole-genome TE-annotations and intact LTR retrotransposons identified by EDTA [54].

Proteins of diploid strawberry *F. vesca* were collected for all-against-all alignments to predicted proteins for octoploid strawberry 'Florida Brilliance'. These alignments were passed to MCScanX to identify synteny blocks [55]. DNA level synteny between *F. vesca*, *F.* × *ananassa*, *F. chiloensis* and two phased genome assemblies of 'Florida Brilliance' were all plotted using D-GENIES [56] with default parameters after aligning with minimap2.

Genome annotation

Transposable elements (TEs) were annotated using EDTA v1.9.6 with default parameters [57]. The TE annotation library was generated by EDTA in a separate run. TE regions of both haploid assemblies were masked by ReapeatMasker v4.1.1 provided with the repeat library. Simple

sequence repeats (SSRs) or microsatellites were mined using SSR Finder [58] on Genome Sequence Annotation Server v6.0 (GenSAS;https://www.gensas.org). Telomeric repeats were annotated using BIOSERF [59].

To increase the accuracy of gene annotation, we generated a transcriptome assembly containing transcripts from 'Florida Brilliance' and *F*. × *ananassa* expression data publicly available. Sixty-seven octoploid strawberry RNA-Seq libraries were downloaded from NCBI sequence read archive (SRA) (https://www.ncbi.nlm.nih.gov/sra; Supplementary file 1). Octoploid strawberry 'Royal Royce' Iso-Seq reads were trimmed using Trimmomatic version 0.39 and mapped to two phased-assemblies using HISAT v2.2.1 [60] with default parameters. The 'Florida Brilliance' Iso-Seq reads were aligned to the assemblies using minimap v2.2.1 [61]. Reads alignment were converted to Binary alignmented map (BAM) format with samtools. Reference-guided transcriptome assembly was performed using StringTie v2.1.4 [62] with the Iso-Seq and RNA-Seq alignments as input. StringTie2 was run with default parameters for RNA-Seq alignment, and with the addition of long read (-L) mode for Iso-Seq alignments. Mikado v2 [63] was used to generate a non-redundant set of transcript assemblies with best-scoring transcript evidence at each locus. Match scores were measured for all transcriptome assemblies against the UniProt protein database using BLASTX. TransDecoder v5.5.0 (https://github.com/TransDecoder/TransDecoder) were used to predict the best six-frame translations of the transcriptome assemblies from StringTie2, then splice junctions for all merged RNA alignments were predicted with Portcullis v1.2.2 [64]. The Mikado scoring for any transcript assemblies derived from Iso-Seq alignments was modified over RNA-Seq alignments. TransDecoder was used to filter non-redundant, polished transcripts generated by Mikado to obtain best ORF scores. The 'Florida Brilliance' genome assembly was annotated using GenSAS v6.0 [58]. The Iso-Seq and RNA-Seq alignments were used to predict gene models using braker2. Functions of predicted gene models were annotated based on alignment using BlastP v2.2.28 to the UniProtKB database [65].

Construction of RNAi and overexpression vectors

Hairpin structures were designed for Fxa6Bg1786920 and Fxa6Bg1786910. Hairpin inserts were composed of a 300-bp stem, 100 bp loop, and *aatt*B sites for Gateway® cloning (Table S10).

The 300-bp stem in each of the inserts was complementary to the first coding DNA sequence (CDS) region of genes Fxa6Bg1786920 and Fxa6Bg1786910. The 758-bp fragments were synthesized using GeneArt^{TM} gene synthesis (Thermo Fisher Scientific, Waltham, MA, USA). The inserts were individually ligated upstream of the *E. coli* sites to the vector pMK-QR containing the kanamycin selection gene. Vector pMK-QR containing the hairpin fragments were used in the Gateway® protocol for Fxa6Bg1786920 and Fxa6Bg1786910, independently. Hairpins were cloned into the Gateway® pDONR™/Zeo vector (Thermo Fisher Scientific, Waltham, MA, USA) using standard procedures. After checking insert identity, the fragments were inserted into the RNAi Gateway® vector pK7GWIWG2(I). Vectors containing the constructs and empty vector were separately inserted into *Agrobacterium tumefaciens* strain EHA105 by using an adapted freeze-thaw method [66]. The transformed cells were tested using PCR for the presence or absence of RNAi constructs (Table S3). For overexpression studies, the coding region of gene (CDS) Fxa6Bg1786910 was obtained based on the 'Florida Brilliance' genome annotation and the corresponding CDS sequence was synthesized by Novogene Co., Ltd. (Beijing, China). The Fxa6Bg1786910 synthesized gene fragment was cloned into the Gateway® pDONR™/Zeo entry vector (Thermo Fisher Scientific, Waltham, MA, USA) following the manufacturer protocol. The pDONR™/Zeo::Fxa6Bg1786910 entry vector was recombined into overexpression vector as pMDC83 containing 2 × CaMV35s promoter through LR reaction to generate overexpression clone as pMDC83::Fxa6Bg1786910.

Fruit transient assay

Agrobacterium-mediated fruit transformation was performed as described by Pi, Gao [67] and Zhao, Mao [68] with modifications. Three varieties, 17.20-51 (*Ca1Ca1*), 'Florida Brilliance' (*Ca1ca1*), and 16.74-68 (*ca1ca1*), are used for the *Agrobacterium*-mediated fruit transient assay. The *A. tumefaciens* EHA105 strain containing RNAi and overexpression constructs were revived by transferring cells from the 1:1, water: 50% glycerol solution with sterilized toothpicks in 5 ml of Luria Broth (LB) medium with rifampicin (15 mg/L) and spectinomycin (100 mg/L) two days before fruit agroinfiltration. Revived *A. tumefaciens* was transferred to 5 ml of LB medium and was allowed to grow for 24 h at 28 °C and 230 rpm. Concentrations of bacterial cells were measured with OD_{600} using a Biomate 3S UV-visible spectrophotometer (Thermo Scientific).

When cultures reached OD_{600} of ~ 1, 1.25 ml was transferred to 250 ml of LB in a flask. *A. tumefaciens* grew for about 18 h at 28 °C, 230 rpm until reaching an OD_{600} value of ~ 1. Bacterial cells were collected by centrifuging the culture in 50-ml tubes at 4,000 rpm for 20 min. *Agrobacterium* cells were resuspended in 600 ml of activation buffer (4.43 gr/L Murashige and Skoog medium, 10 mM $MgCl_2$, 200 µM acetosyringone, 10 mM 2-(N-morpholino) ethane sulfonic acid (MES), pH = 5.8). Concentration was fixed at OD_{600} = 0.5 for replication 1 and 0.8 for reps 2, 3, 4, and 5. The activated solution was kept in an orbital shaker for three hours at room temperature at 100 rpm.

Ninety green fruits of each genotype were harvested in the early morning. Healthy uniform at a similar development stage of white fruit was preferred [68]. Pedicels were carefully removed to avoid contamination during the experiment. Fruit was disinfested by immersion in a 0.7% solution of sodium hypochlorite (bleach) for six minutes, rinsed with sterilized water, and placed in the hood for 20 minutes to dry [69]. Agroinfiltration was performed by injecting the activated solution for each of the constructs into the green fruit using 5 ml - syringes until saturation [68]. Sterilized water was poured into the plastic containers to maintain humidity. Agroinfiltrated fruit was kept under 16 hours of daylight and 8 h of dark conditions at room temperature.

Agroinfiltrated fruit was inoculated with the *C. acutatum* species complex five days after agroinfiltration. Inoculum was produced by growing the three isolates of *C. acutatum* (*C. nymphaeae*) 02-163, 02-179, and 03-32 separately on potato-dextrose agar (PDA) at room temperature with constant light for seven days until sporulation. Plates were flooded with sterile-distilled water when growth turned orange and reached near the border of the plate. A spore suspension for each isolate was passed through cheesecloth to remove dislodged mycelia. A final suspension was adjusted to 1×10^6 conidia/ml by combining equal concentrations of each isolate. Fruits were inoculated with a single 20-µl drop of *C. acutatum* inoculum in the hood [70]. External and internal symptoms were analyzed using the software ImageJ 1.53e. Area of fruit, area of external symptoms, area of halves of cut fruit, and area of internal symptom were obtained. Agroinfiltration, inoculation, and phenotype scoring were performed five times. Means were compared and separated using a two-way analysis of variance (ANOVA) and the least significant difference (LSD) test in R software (R Core Team 2000).

RNA extraction and qRT-PCR analysis

RNA was extracted by using the Spectrum™ Plant Total RNA Kit (Sigma-Aldrich, MO, USA) as recommended by the manufacturer. RNA concentration and quality were assessed using Nanodrop 8000 Spectrophotometer (Thermo Scientific) and Qubit™ RNA broad range (BR) assay kits. cDNA for the knockdown and gene expression samples was synthesized in a 20 µl reaction composed of 4 µl of LunaScript® RT SuperMix Kit (New England, MA, USA) and 16 µl containing 500 ng of total RNA. The qRT-PCR experiment was performed in triplicates of 5 µl reactions containing 2.5 µl of 2 × EvaGreen® based Forget-Me-Not™ qPCR (Biotium, CA, USA) master mix, 0.4 µl of 0.5 µM forward primer solution, 0.4 µl of 0.5 µM reverse primer solution, 1 µl of diluted cDNA and 0.7 µl of DI H_2O. The cDNA dilution at 5 ng/µl was used for the PCR reactions containing primers for the housekeeping gene *FaGapDH2* and the cDNA dilution at 20 ng/µl for PCR reactions with primers for genes Fxa6Bg1786920 and Fxa6Bg1786910 (Table S14). The reactions were carried out in a 384-well PCR plate in a LightCycler®480 II Instrument (Roche, Switzerland) for both PCR and HRM. Conditions of PCR were: preincubation at 95 °C for 5 min, initial denaturation at 95 °C for 20 s, 40 cycles of denaturation at 95 °C for 20 s, annealing at 62 °C for 10 s, and extension at 72 °C for 10 s. HRM conditions were 95 °C for 5 s, 65 °C for 1 min, and 97 °C continuous. Finally, the PCR product was cooled to 40 °C for 30 s to allow heteroduplex formation. The raw cycle threshold values (Cp) from qRT-PCR runs were used to calculate relative expression using the ΔCt method.

Gene expression profiling after *C. acutatum* inoculation

Forty-five white or white fruit with slight pink hue from homozygous resistant selection (*Ca1Ca1*) 17.20-51, heterozygous resistant (*Ca1ca1*) 'Florida Brilliance' and homozygous susceptible selection (*ca1ca1*) 16.74-68 were harvested in the early morning. Healthy uniform fruit at similar development stages was preferred. Fruit disinfestation, classification, arrangement, and *C. acutatum* inoculation were done as previously described in the knockdown and overexpression experiments. Three biological replications composed of tissue from three fruits each were taken every day at the same time at five-time points: 0, 24, 48, 72, and 96 h post-inoculation (hpi) for each genotype. Samples collected at 0 hpi were taken from non-inoculated fruit. Samples collected at 24, 48, 72, and 96 hpi were obtained by measuring 2-cm

squares around the lesion caused by the *C. acutatum* inoculation. Fruit tissue was cut into small pieces and immediately frozen using liquid nitrogen. Samples were kept at -80 °C.

Data Availability

High-throughput sequencing data analyzed in the present study are available under NCBI BioProject PRJNA888562. The Hi-C data are available through the NCBI SRA database (SRR21850459) and PacBio long-read sequencing data have been deposited in the NCBI SRA (Accession: SRR21850458). The Iso-Seq data are available through NCBI SRA; crown (SRR21895706), flower (SRR21895705), green fruit (SRR21895704), red fruit (SRR21895703), leaf (SRR21895702), and root (SRR21895701). The chromosome-level genome assembly, annotation files, and other supporting data are available via genome database for rosaceae (GDR).

Abbreviations

LAI: LTR assembly Index; BUSCO: Benchmarking Universal Single-Copy Orthologs; HiFi: high fidelity; LLD: legume lectin domain; T2T: telomere-to-telomere; AFR: anthracnose fruit rot; ARN: anthracnose root necrosis; TE: transposable element; ANOVA: analysis of variance; LSD: least significant difference; SSRs: Simple sequence repeats; BR: broad range; SRA: sequence read archive; H-1: haplotype-1; H-2: haplotype-2;

Competing interests

The authors declare no competing interests.

Acknowledgement

Authors acknowledge Florida Strawberry Growers Association and strawberry breeding groups at UF/IFAS Gulf Coast Research and Education Center for their support of this study. This research is supported by grants from the United States Department of Agriculture National Institute of Food and Agriculture (NIFA) Specialty Crops Research Initiative (SCRI) “Delivering Breeding and Management Solutions to Prevent Losses to Emerging and Expanding Disease Threats in Strawberry” under award number (#2022-51181-38328).

Authors' contributions

S.L. conceived and designed the study; V.S. constructed genetic linkage map; N.S. and Y.J. conducted transient assay and analyzed the phenotypic data; H.H. and N.S. wrote the draft manuscript; Z.F., C.B., S.V and V.F. discussed the results and improved and revised the manuscript. All authors reviewed the manuscript.

Figure Legends

Figure 1. Dotplot of 'Florida Brilliance' H-1 and H-2 assemblies showing their collinearity (A) and collinearity between H-2 and diploid *F. vesca* ver 4.0 (B).

Figure 2. A telomere-to-telomere haplotype-phased genome assembly for octoploid strawberry 'Florida Brilliance'. Triangles (orange) represent regions with telomere repeats (5'-TTTAGGG-3'). Multiple triangles represent interstitial telomere-like sequence. Circles (purple) indicate centromeres, with low gene density and high density of repetitive sequences including LTR RTs and mini satellites. Suffixes 'a' and 'b' were affixed to the chromosomes to denote each haplotype genome such as haplotype-1 or haplotype-2.

Figure 3. Collinearity analysis between the 'Florida Brilliance' (FaFB1) genome and other octoploid strawberry genomes. (A) Comparative genome analysis of 'Florida Brilliance' (FaFB1) and 'Royal Royce' (FaRR1), (B) FaFB1 vs. *F. chiloensis*, and (C) FaFB1 vs. *F. virginiana* [28].

Figure 4. The number of genes predicted in the subgenome A, B, C, and D of the H-1 genome assembly for 'Florida Brilliance'. The number of genes is indicated above each chromosome. Subgenome A – *F. vesca*, Subgenome B – *F. iinumae*, Subgenome C and D – proposed to *F. viridis* and *F. nipponica*.

Figure 5. Identification of candidate genes, *LRK10A* (Fxa6Bg1786910) and *LRK10B* (Fxa6Bg1786920), in the *FaRCa1* region of 'Florida Brilliance' genome (FaFB1). **(A)** *FaRCa1* in Chr 6B of FaFB1 and positions of IStraw 35 Affymterix Axiom® and 50K FanaSNP array SNPs. The 50K FanaSNP Array probe 'AX-89896208' and 'AX-89838962' were located at

16.18 Mb and 16.32 Mb, respectively. **(B-C)** Gene structure, amino acid sequences, and 3D protein structure of resistant allele (Fxa6Bg1786910) and susceptible allele (Fxa6Bg2100330). Gray rectangles and black lines represent exons and introns, respectively. The green box indicates insertions. Red lines represent the SNP position between resistant allele in H-1 and susceptible allele in 'Florida Brilliance' H-2. **(D-E)** Gene structure, amino acid sequences, and 3D protein structure of resistant allele (Fxa6Bg1786920) and susceptible allele (Fxa6Bg2100340). Gray rectangles and black lines represent exons and introns, respectively. The green box indicates deletion. Red lines represent the SNP position between resistant allele in H-1 and susceptible allele in 'Florida Brilliance' H-2.

Figure 6. Internal symptoms of anthracnose fruit rot in fruit transient knockdown and transient overexpression. (**A**) Phenotype of transient knockdown of *LRK10A* and *LRK10B* in strawberry fruit from three genotypes: FL 16.74-68 (*ca1ca1*), 'Florida Brilliance' (*Ca1ca1*), and FL 17.20-51 (*Ca1Ca1*). *Agrobacterium*-infiltrated fruits with three treatments: Empty Vector, pK7::*LRK10A* RNAi-mediated knockdown and pK7::*LRK10B* RNAi-mediated knockdown. **(B)** Internal Sympomatic Area (%) in fruits from genotypes and treatments described in (A). (**C**) Phenotype of transient overexpression of *LRK10A* in strawberry fruit from three genotypes: FL 16.74-68 (*ca1ca1*), 'Florida Brilliance' (*Ca1ca1*), and FL 17.20-51 (*Ca1Ca1*). *Agrobacterium*-infiltrated fruits with three treatments: Empty Vector, pMDC83::*LRK10A*. (**D**) Internal Sympomatic Area (%) in fruits from genotypes and treatments described in (C). Error bars represent SE based on three biological replicates. The white line represents a scale of 1 cm, Lowercase letters indicate significantly different means using least significant difference test (α = 0.05). Bars indicate standard errors.

Supplementary Figures.

Figure S1. Haplotype-phased assembly of an octoploid strawberry genome using Hifi and Hi-C data.

Figure S2. Chromosome length of 'Florida Brilliance', 'Royal Royce' and 'Camarosa' assemblies. 'Florida Brilliance' and 'Royal Royce' consist of two phased assemblies (H-1 and H-2).

Figure S3. Smudgeplot for 'Florida Brilliance' (https://github.com/KamilSJaron/smudgeplot/). The brightness of each smudge was determined by the number of k-mer pairs. The coloration indicates the approximate number of *k*-mer pairs per bin.

Figure S4. Hi-C contact map of H-1 **(A)** and H-2 **(B)** genome assemblies of 'Florida Brilliance'. Each red pixel represents a Hi-C pair. The dominant visual feature in every Hi-C heat map is the strong diagonal representing the Hi-C pairs of loci uniquely anchored in each haplotype-phased assembly.

Figure S5. Genetic positions of 10,269 markers from the linkage map for 'Florida Brilliance' against the corresponding physical positions in the 'Florida Brilliance' genome assembly.

Figure S6. Dotplot of collinear gene pairs between 'Florida Brilliance' and *F. vesca*. Homologous genes of H-1 **(A)** and H-2 **(B)** assemblies were compared against diploid *F. vesca*. Relative expression level analysis of selected genes by qRT-PCR in different time stages of inoculated strawberry against *C. acutatum.*

Figure S7. Dotplot of 'Florida Brilliance' H-2 assembly to *F.* × *ananassa* cv. Royal Royce **(A)**, *F. chiloensis* **(B)**, and *F. virginiana* **(C)**. Dot plots are produced using the DGENIE software and alignments with minimap2.

Figure S8. Comparative genomics analysis using Mauve alignment in Geneious Prime® 2022.0.1 with the *FaRCa1* in Chr 6B from 'Florida Brilliance' (A) and regions homologous to *FaRCa1* in sub-genomes Chr 6B from published genomes 'Royal Royce', FL 15.89-25 and 'Camarosa' (B). Resistant alignments showed three locally collinear blocks (LCB) when aligned with homologous *FaRCa1* regions in Chr 6B from 'Royal Royce', FL15.89-25 and 'Camarosa'. *FaRCa1* in Chr 6B from 'Florida Brilliance' H-1 assembly showed different length with others.

Figure S9. Relative gene expression for *LRK10A* (A) and *LRK10B* (B) after *C. acutatum* inoculation for genotypes *ca1ca1*, *Ca1ca1 and Ca1Ca1***.** Each bar depicts the log 2 -transformed mean ± S.E of three biological and three technical replicates.

Figure S10. Validation of candidate genes *LRK10A* and *LRK10B* with qRT-PCR in Transient Assays **(A)** Expression levels of *LRK10A* and *LRK10B* in RNAi knockdown transient assays conducted in *ca1ca1*, *Ca1ca1*, and *Ca1Ca1* cultivars. **(B)** Expression levels of *LRK10A* in

overexpression vector constructs in *ca1ca1* and *Ca1ca1* cultivars. Error bars represent the standard deviation based on three biological replicates. Statistical analysis using a student t-test indicated significant differences denoted as $***P < 0.001$, $**P < 0.01$, $*P < 0.05$ when compared to the empty vector control in transient assay samples.

REFERENCES

1. Duchesne AN. Histoire naturelle des fraisiers, contenant les vues d'economie reunies a la botanique; & suivie de remarques particulieres sur plusieurs points qui ont rapport a l'histoire naturelle generale. Par m. Duchesne fils. chez Didot le jeune, rue de Hurepoix; 1766.
2. Edger PP, Poorten TJ, VanBuren R, Hardigan MA, Colle M, McKain MR, et al. Origin and evolution of the octoploid strawberry genome. Nature genetics. 2019;51 3:541-7.
3. Barbey CR, Hogshead MH, Harrison B, Schwartz AE, Verma S, Oh Y, et al. Genetic Analysis of Methyl Anthranilate, Mesifurane, Linalool, and Other Flavor Compounds in Cultivated Strawberry (Fragaria× ananassa). Frontiers in Plant Science. 2021:718.
4. Hardigan MA, Lorant A, Pincot DD, Feldmann MJ, Famula RA, Acharya CB, et al. Unraveling the complex hybrid ancestry and domestication history of cultivated strawberry. Molecular biology and evolution. 2021;38 6:2285-305.
5. Barbey CR, Lee S, Verma S, Bird KA, Yocca AE, Edger PP, et al. Disease resistance genetics and genomics in octoploid strawberry. G3: Genes, Genomes, Genetics. 2019;9 10:3315-32.
6. Tennessen JA, Govindarajulu R, Ashman T-L and Liston A. Evolutionary origins and dynamics of octoploid strawberry subgenomes revealed by dense targeted capture linkage maps. Genome biology and evolution. 2014;6 12:3295-313.
7. Hardigan MA, Feldmann MJ, Pincot DD, Famula RA, Vachev MV, Madera MA, et al. Blueprint for phasing and assembling the genomes of heterozygous polyploids: application to the octoploid genome of strawberry. BioRxiv. 2021.
8. Cheng H, Jarvis ED, Fedrigo O, Koepfli K-P, Urban L, Gemmell NJ, et al. Robust haplotype-resolved assembly of diploid individuals without parental data. arXiv preprint arXiv:210904785. 2021.

9. Porubsky D, Ebert P, Audano PA, Vollger MR, Harvey WT, Marijon P, et al. Fully phased human genome assembly without parental data using single-cell strand sequencing and long reads. Nature biotechnology. 2021;39 3:302-8.

10. Chen J, Wang Z, Tan K, Huang W, Shi J, Li T, et al. A complete telomere-to-telomere assembly of the maize genome. Nature Genetics. 2023:1-11.

11. Belser C, Baurens F, Noel B, Martin G, Cruaud C, Istace B, et al. Telomere-to-telomere gapless chromosomes of banana using nanopore sequencing. Commun. Biol. 4: 1047. 2021.

12. Zhou Y, Xiong J, Shu Z, Dong C, Gu T, Sun P, et al. The telomere-to-telomere genome of Fragaria vesca reveals the genomic evolution of Fragaria and the origin of cultivated octoploid strawberry. Horticulture Research. 2023;10 4:uhad027.

13. Salinas N, Verma S, Peres N and Whitaker VM. *FaRCa1*: a major subgenome-specific locus conferring resistance to *Colletotrichum acutatum* in strawberry. Theor Appl Genet. 2019;132 4:1109-20. doi:10.1007/s00122-018-3263-7.

14. Salinas N, Fan Z, Peres N, Lee S and Whitaker VM. *FaRCa1* confers moderate resistance to the root necrosis form of strawberry anthracnose caused by *Colletotrichum acutatum*. HortScience horts. 2020;55 5:693-8. doi:10.21273/hortsci14807-20.

15. Denoyes-Rothan B, Lerceteau-Köhler E, Guérin G, Bosseur S, Bariac J, Martin E, et al. QTL analysis for resistances to Colletotrichum acutatum and Phytophthora cactorum in octoploid strawberry (Fragaria× ananassa). 2004.

16. Hardigan MA, Feldmann MJ, Lorant A, Bird KA, Famula R, Acharya C, et al. Genome synteny has been conserved among the octoploid progenitors of cultivated strawberry over millions of years of evolution. Frontiers in plant science. 2020;10:1789.

17. Afzal AJ, Wood AJ and Lightfoot DA. Plant receptor-like serine threonine kinases: roles in signaling and plant defense. Molecular Plant-Microbe Interactions. 2008;21 5:507-17.

18. Morris ER and Walker JC. Receptor-like protein kinases: the keys to response. Current opinion in plant biology. 2003;6 4:339-42.

19. Lim CW, Yang SH, Shin KH, Lee SC and Kim SH. The AtLRK10L1. 2, Arabidopsis ortholog of wheat LRK10, is involved in ABA-mediated signaling and drought resistance. Plant cell reports. 2015;34:447-55.

20. Hardigan MA, Feldmann MJ, Pincot DD, Famula RA, Vachev MV, Madera MA, et al. Blueprint for phasing and assembling the genomes of heterozygous polyploids: application to the octoploid genome of strawberry. BioRxiv. 2021:2021.11. 03.467115.

21. Lee H-E, Manivannan A, Lee SY, Han K, Yeum J-G, Jo J, et al. Chromosome level assembly of homozygous inbred line ‘Wongyo 3115’facilitates the construction of a high-density linkage map and identification of QTLs associated with fruit firmness in octoploid strawberry (Fragaria× ananassa). Frontiers in plant science. 2021;12:696229.

22. Shirasawa K, Hirakawa H, Nakayama S, Sasamoto S, Tsuruoka H, Minami C, et al. A chromosome-scale strawberry genome assembly of a Japanese variety, Reikou. bioRxiv. 2021.

23. Mao J, Wang Y, Wang B, Li J, Zhang C, Zhang W, et al. High-quality haplotype-resolved genome assembly of cultivated octoploid strawberry. Horticulture Research. 2023;10 1:uhad002.

24. Fan Z, Tieman DM, Knapp SJ, Zerbe P, Famula R, Barbey CR, et al. A multi-omics framework reveals strawberry flavor genes and their regulatory elements. New Phytologist. 2022;236 3:1089-107.

25. Cauret C, Mortimer SM, Roberti MC, Ashman T-L and Liston A. Chromosome-scale assembly with a phased sex-determining region resolves features of early Z and W chromosome differentiation in a wild octoploid strawberry. G3 Genes| Genomes| Genetics. 2022.

26. Peska V and Garcia S. Origin, diversity, and evolution of telomere sequences in plants. Frontiers in Plant Science. 2020;11:117.

27. Ou S, Chen J and Jiang N. Assessing genome assembly quality using the LTR Assembly Index (LAI). Nucleic acids research. 2018;46 21:e126-e.

28. Jin X, Du H, Zhu C, Wan H, Liu F, Ruan J, et al. Haplotype-resolved genomes of wild octoploid progenitors illuminate genomic diversifications from wild relatives to cultivated strawberry. Nature Plants. 2023;9 8:1252-66.

29. Chandler CK, Mertely JC and Peres N. Resistance of selected strawberry cultivars to anthracnose fruit rot and Botrytis fruit rot. In: *V International Strawberry Symposium 708* 2004, pp.123-6.

30. Seijo TE, Chandler CK, Mertely JC, Moyer C and Peres NA. Resistance of strawberry cultivars and advanced selections to anthracnose and Botrytis fruit rots. In: *Proceedings of the Florida State Horticultural Society* 2008, pp.246-8.

31. Whitaker VM, Chandler CK, Santos BM, Peres N, do Nascimento Nunes MC, Plotto A, et al. Winterstar™(‘FL 05-107’) strawberry. HortScience. 2012;47 2:296-8.

32. van de Weg E, Verma S, Bassil N, Harrison R, Monfort A, Hidalgo J, et al. Development and evaluation of the Axiom® IStraw35 384HT array for the allo-octoploid cultivated strawberry Fragaria× ananassa. In: *VIII International Strawberry Symposium 1156* 2016, pp.75-82.

33. Salinas N, Fan Z, Peres N, Lee S and Whitaker VM. FaRCa1 confers moderate resistance to the root necrosis form of strawberry anthracnose caused by Colletotrichum acutatum. HortScience. 2020;55 5:693-8.
34. Feuillet C, Reuzeau C, Kjellbom P and Keller B. Molecular characterization of a new type of receptor-like kinase (wlrk) gene family in wheat. Plant molecular biology. 1998;37:943-53.
35. Tang D, Wang G and Zhou J-M. Receptor kinases in plant-pathogen interactions: more than pattern recognition. The Plant Cell. 2017;29 4:618-37.
36. Saijo Y, Loo EPi and Yasuda S. Pattern recognition receptors and signaling in plant–microbe interactions. The Plant Journal. 2018;93 4:592-613.
37. Feuillet C, Schachermayr G and Keller B. Molecular cloning of a new receptor-like kinase gene encoded at the Lr10 disease resistance locus of wheat. The Plant Journal. 1997;11 1:45-52.
38. Feuillet C, Travella S, Stein N, Albar L, Nublat A and Keller B. Map-based isolation of the leaf rust disease resistance gene Lr10 from the hexaploid wheat (Triticum aestivum L.) genome. Proceedings of the National Academy of Sciences. 2003;100 25:15253-8.
39. Fukasawa Y, Ermini L, Wang H, Carty K and Cheung M-S. LongQC: a quality control tool for third generation sequencing long read data. G3: Genes, Genomes, Genetics. 2020;10 4:1193-6.
40. Kokot M, Długosz M and Deorowicz S. KMC 3: counting and manipulating k-mer statistics. Bioinformatics. 2017;33 17:2759-61.
41. Ranallo-Benavidez TR, Jaron KS and Schatz MC. GenomeScope 2.0 and Smudgeplot for reference-free profiling of polyploid genomes. Nature communications. 2020;11 1:1-10.
42. Li H and Durbin R. Fast and accurate short read alignment with Burrows–Wheeler transform. bioinformatics. 2009;25 14:1754-60.
43. Li H, Handsaker B, Wysoker A, Fennell T, Ruan J, Homer N, et al. The sequence alignment/map format and SAMtools. Bioinformatics. 2009;25 16:2078-9.
44. Quinlan AR and Hall IM. BEDTools: a flexible suite of utilities for comparing genomic features. Bioinformatics. 2010;26 6:841-2.
45. Ghurye J, Rhie A, Walenz BP, Schmitt A, Selvaraj S, Pop M, et al. Integrating Hi-C links with assembly graphs for chromosome-scale assembly. PLoS computational biology. 2019;15 8:e1007273.
46. Servant N, Varoquaux N, Lajoie BR, Viara E, Chen C-J, Vert J-P, et al. HiC-Pro: an optimized and flexible pipeline for Hi-C data processing. Genome biology. 2015;16 1:1-11.

47. Alonge M, Soyk S, Ramakrishnan S, Wang X, Goodwin S, Sedlazeck FJ, et al. RaGOO: fast and accurate reference-guided scaffolding of draft genomes. Genome biology. 2019;20 1:1-17.
48. Camacho C, Coulouris G, Avagyan V, Ma N, Papadopoulos J, Bealer K, et al. BLAST+: architecture and applications. BMC bioinformatics. 2009;10 1:1-9.
49. Rhie A, Walenz BP, Koren S and Phillippy AM. Merqury: reference-free quality, completeness, and phasing assessment for genome assemblies. Genome biology. 2020;21 1:1-27.
50. Simão FA, Waterhouse RM, Ioannidis P, Kriventseva EV and Zdobnov EM. BUSCO: assessing genome assembly and annotation completeness with single-copy orthologs. Bioinformatics. 2015;31 19:3210-2.
51. Heinz S, Benner C, Spann N, Bertolino E, Lin YC, Laslo P, et al. Simple combinations of lineage-determining transcription factors prime cis-regulatory elements required for macrophage and B cell identities. Molecular cell. 2010;38 4:576-89.
52. Durand NC, Robinson JT, Shamim MS, Machol I, Mesirov JP, Lander ES, et al. Juicebox provides a visualization system for Hi-C contact maps with unlimited zoom. Cell systems. 2016;3 1:99-101.
53. Ou S and Jiang N. LTR_retriever: a highly accurate and sensitive program for identification of long terminal repeat retrotransposons. Plant physiology. 2018;176 2:1410-22.
54. Ou S, Su W, Liao Y, Chougule K, Agda JR, Hellinga AJ, et al. Benchmarking transposable element annotation methods for creation of a streamlined, comprehensive pipeline. Genome biology. 2019;20 1:1-18.
55. Wang Y, Tang H, DeBarry JD, Tan X, Li J, Wang X, et al. MCScanX: a toolkit for detection and evolutionary analysis of gene synteny and collinearity. Nucleic acids research. 2012;40 7:e49-e.
56. Cabanettes F and Klopp C. D-GENIES: dot plot large genomes in an interactive, efficient and simple way. PeerJ. 2018;6:e4958.
57. Su W, Ou S, Hufford MB and Peterson T. A tutorial of EDTA: extensive de novo TE annotator. Plant Transposable Elements. 2021:55-67.
58. Humann JL, Lee T, Ficklin S and Main D. Structural and functional annotation of eukaryotic genomes with GenSAS. Gene prediction. Springer; 2019. p. 29-51.
59. Somanathan I and Baysdorfer C. A bioinformatics approach to identify telomere sequences. Biotechniques. 2018;65 1:20-5.
60. Kim D, Paggi JM, Park C, Bennett C and Salzberg SL. Graph-based genome alignment and genotyping with HISAT2 and HISAT-genotype. Nature biotechnology. 2019;37 8:907-15.

61. Li H. Minimap2: pairwise alignment for nucleotide sequences. Bioinformatics. 2018;34 18:3094-100.
62. Kovaka S, Zimin AV, Pertea GM, Razaghi R, Salzberg SL and Pertea M. Transcriptome assembly from long-read RNA-seq alignments with StringTie2. Genome biology. 2019;20 1:1-13.
63. Venturini L, Caim S, Kaithakottil GG, Mapleson DL and Swarbreck D. Leveraging multiple transcriptome assembly methods for improved gene structure annotation. GigaScience. 2018;7 8:giy093.
64. Mapleson D, Venturini L, Kaithakottil G and Swarbreck D. Efficient and accurate detection of splice junctions from RNA-seq with Portcullis. GigaScience. 2018;7 12:giy131.
65. Boutet E, Lieberherr D, Tognolli M, Schneider M and Bairoch A. Uniprotkb/swiss-prot. Plant bioinformatics. Springer; 2007. p. 89-112.
66. Weigel D and Glazebrook J. Transformation of *Agrobacterium* using the freeze-thaw method. Cold Spring Harbor Protocols. 2006; doi:10.1101/pdb.prot4666.
67. Pi M, Gao Q and Kang C. Transient expression assay in strawberry fruits. Bio Protoc. 2019;9 11:e3249-e. doi:10.21769/BioProtoc.3249.
68. Zhao Y, Mao W, Chen Y, Wang W, Dai Z, Dou Z, et al. Optimization and standardization of transient expression assays for gene functional analyses in strawberry fruits. Horticulture Research. 2019;6 1:53. doi:10.1038/s41438-019-0135-5.
69. Forcelini BB, Gonçalves FP and Peres NA. Effect of inoculum concentration and interrupted wetness duration on the development of anthracnose fruit rot of strawberry. Plant Disease. 2016;101 2:372-7. doi:10.1094/PDIS-08-16-1175-RE.
70. Forcelini BB, Rebello CS, Wang N-Y and Peres NA. Fitness, competitive ability, and mutation stability of isolates of *Colletotrichum acutatum* from strawberry resistant to QoI fungicides. Phytopathology®. 2018;108 4:462-8. doi:10.1094/phyto-09-17-0296-r.

Figure1

Click here to access/download;Figure;Fig.1.pptx

A

Florida Brilliance (Haplotype-1)

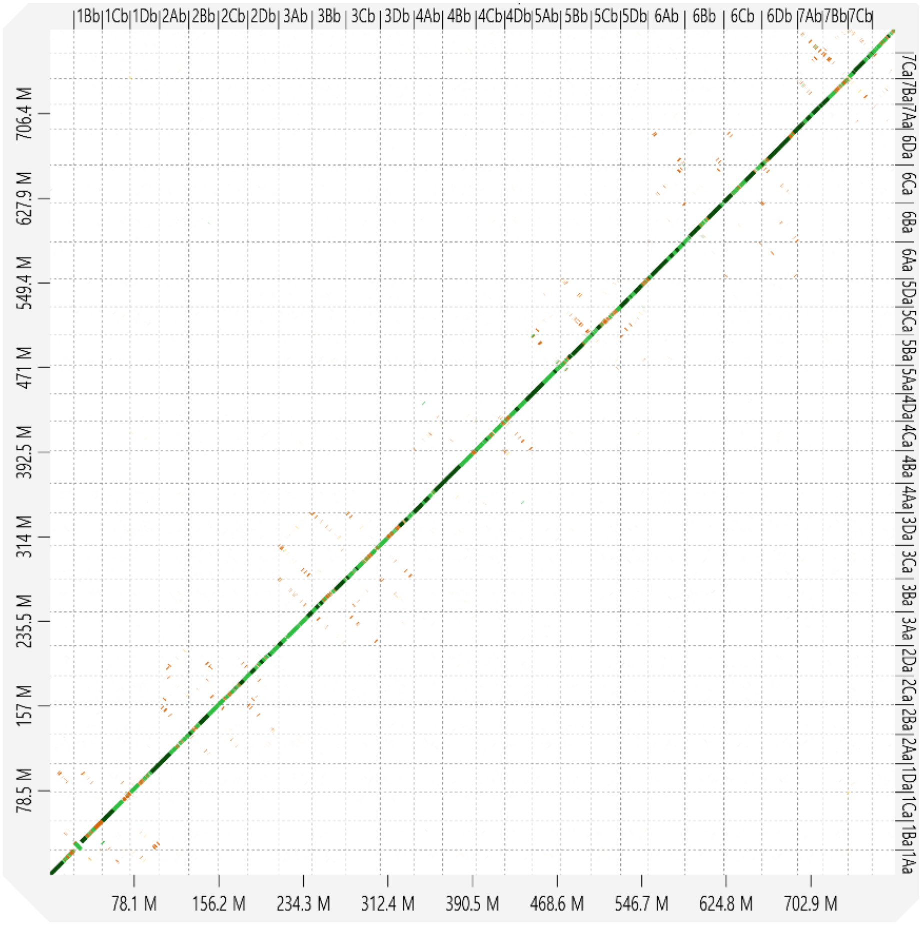

Florida Brilliance (Haplotype-2)

B

***F. vesca* ver4.0 Hawaii**

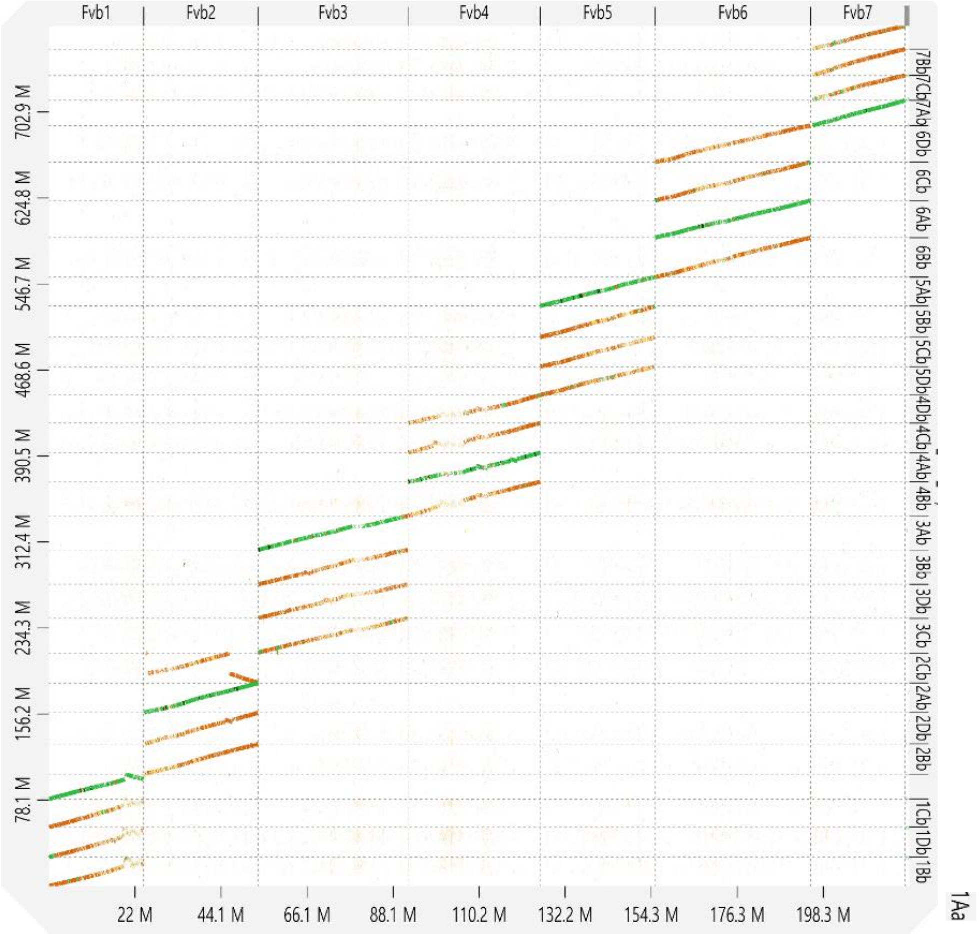

Florida Brilliance (Haplotype-2)

Figure2

Click here to access/download;Figure;Fig.2.pptx

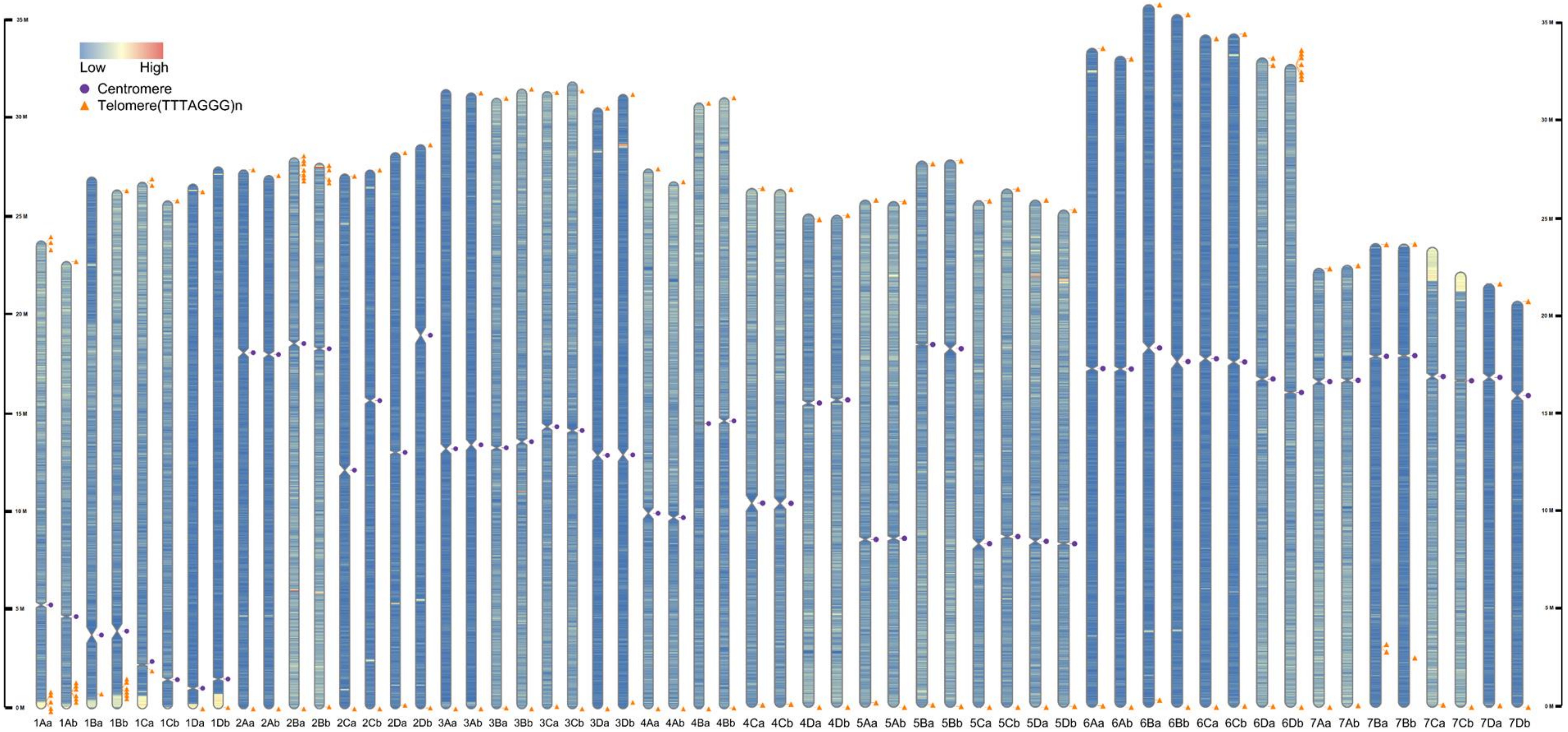

Figure3

Click here to access/download;Figure;Fig.3.pptx

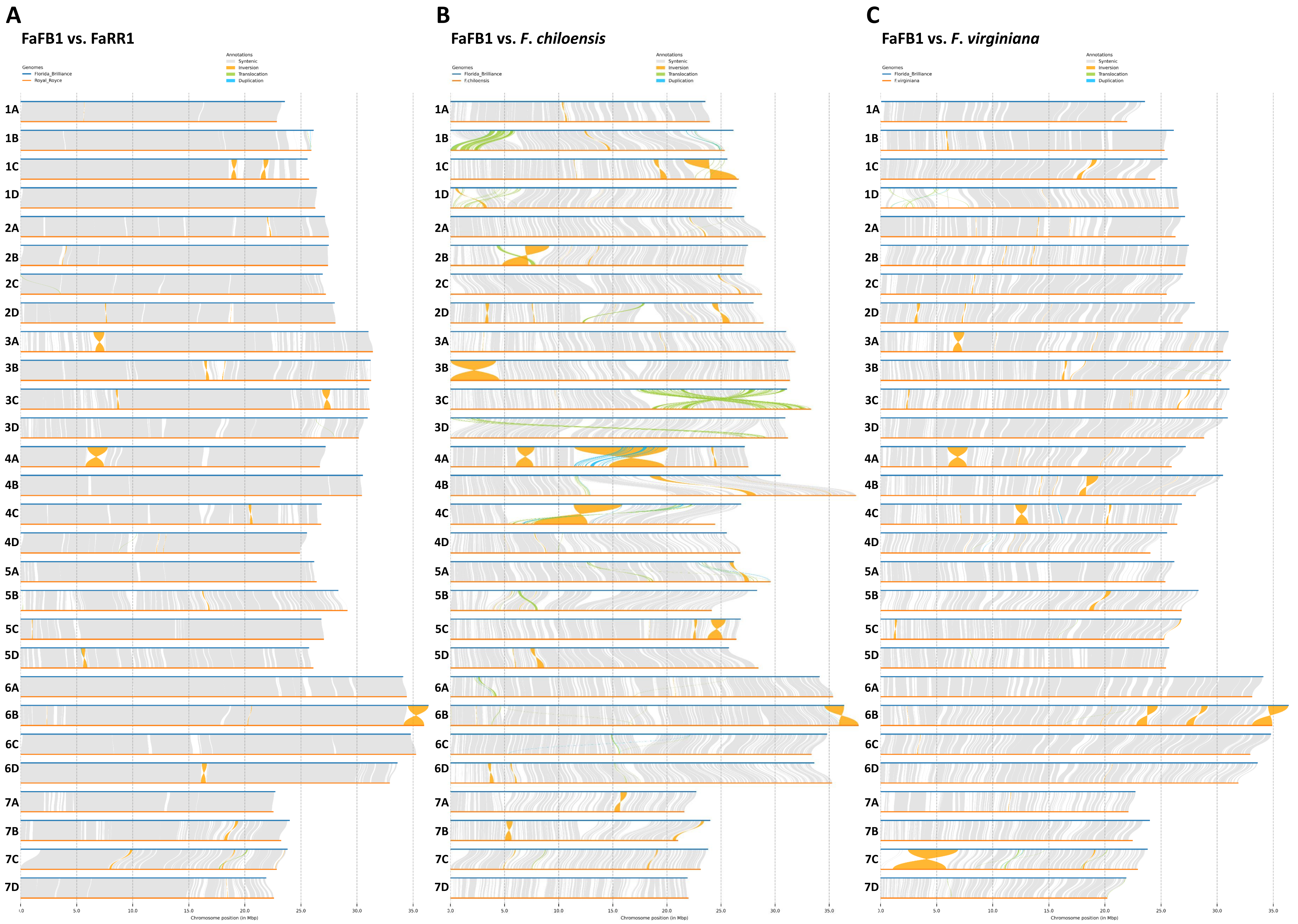

Click here to access/download;Figure;Fig.4.pptx

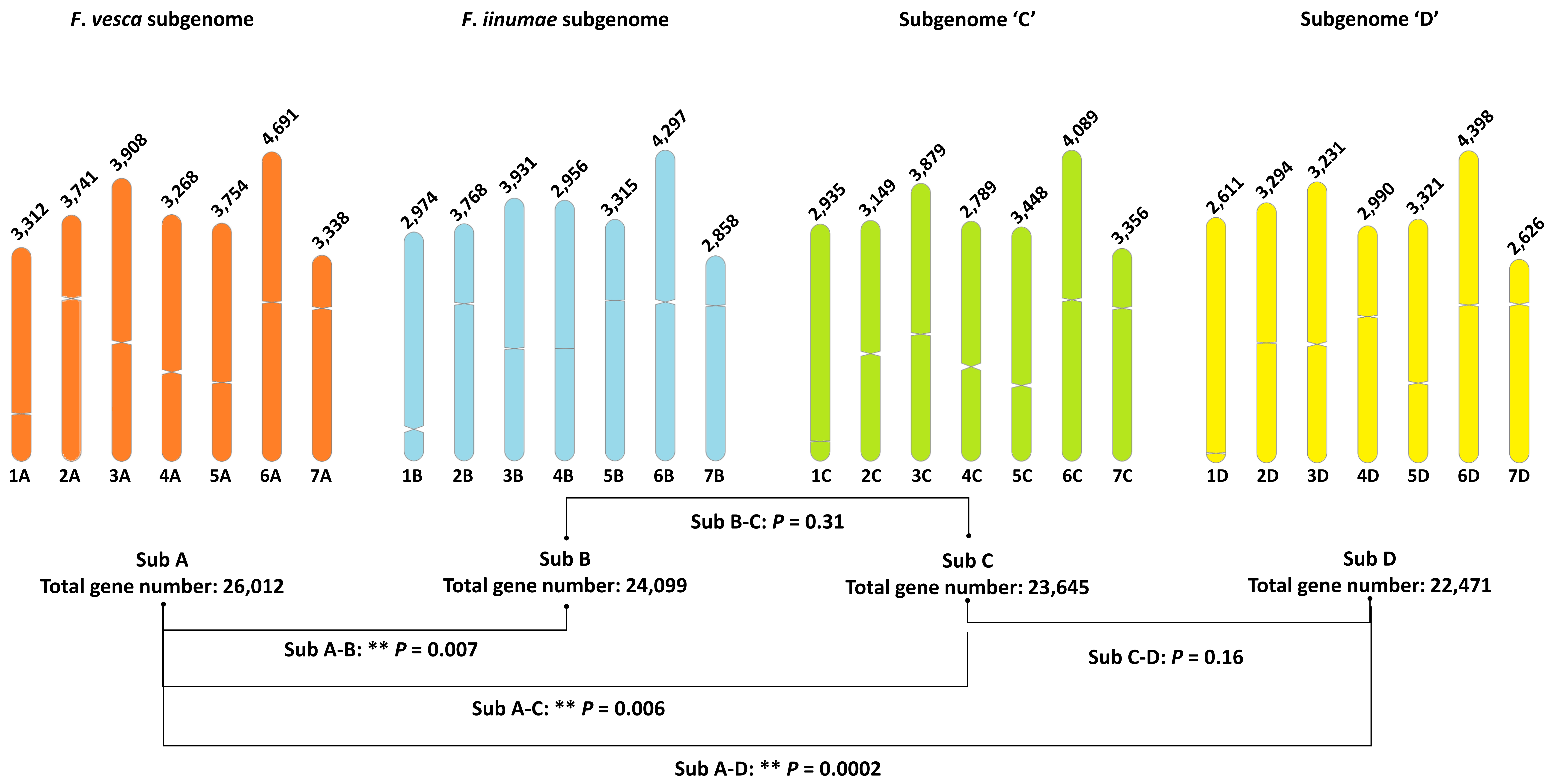

Click here to access/download;Figure;Fig.5.pptx

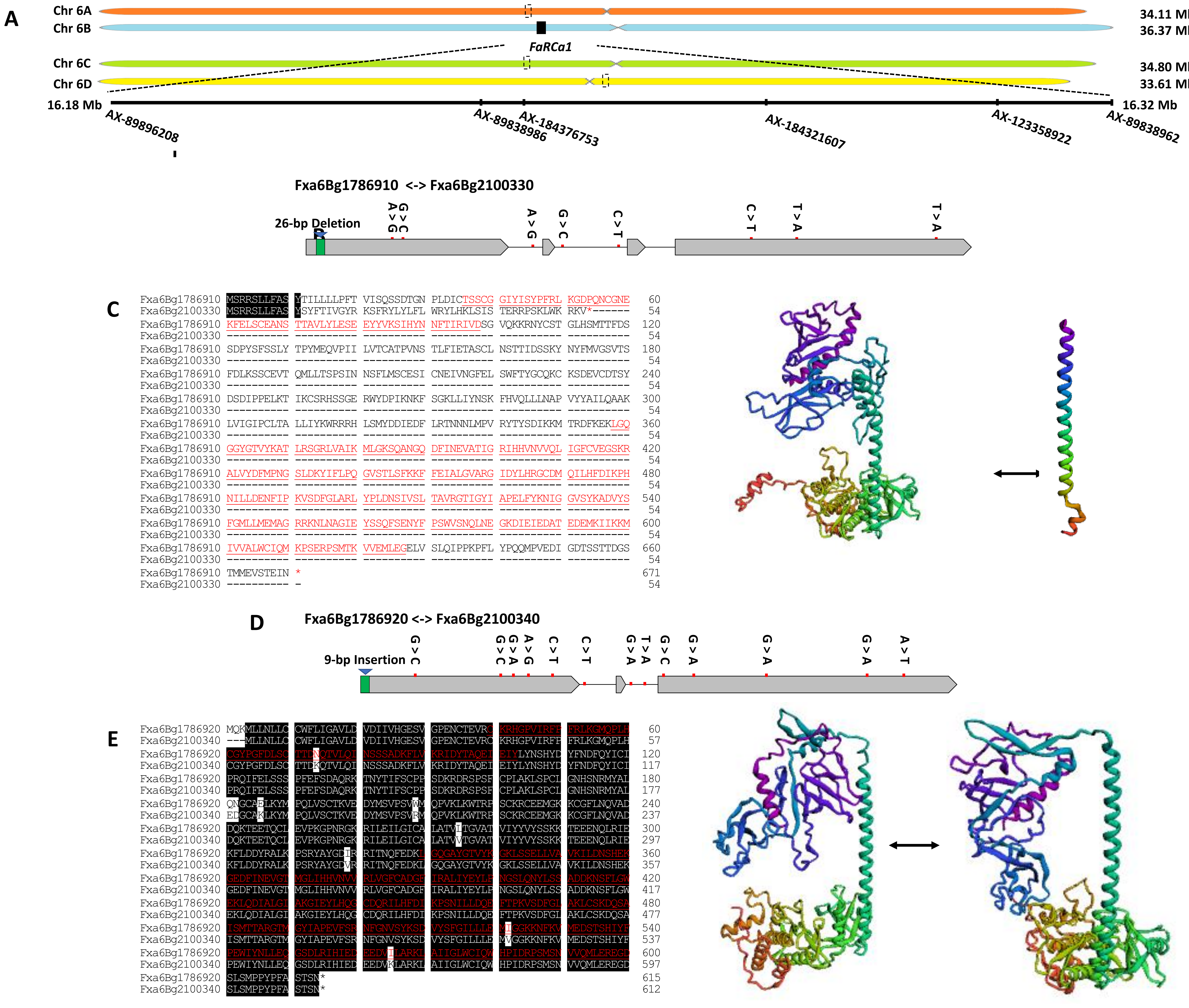

Figure6

Click here to access/download;Figure;Fig.6.pptx

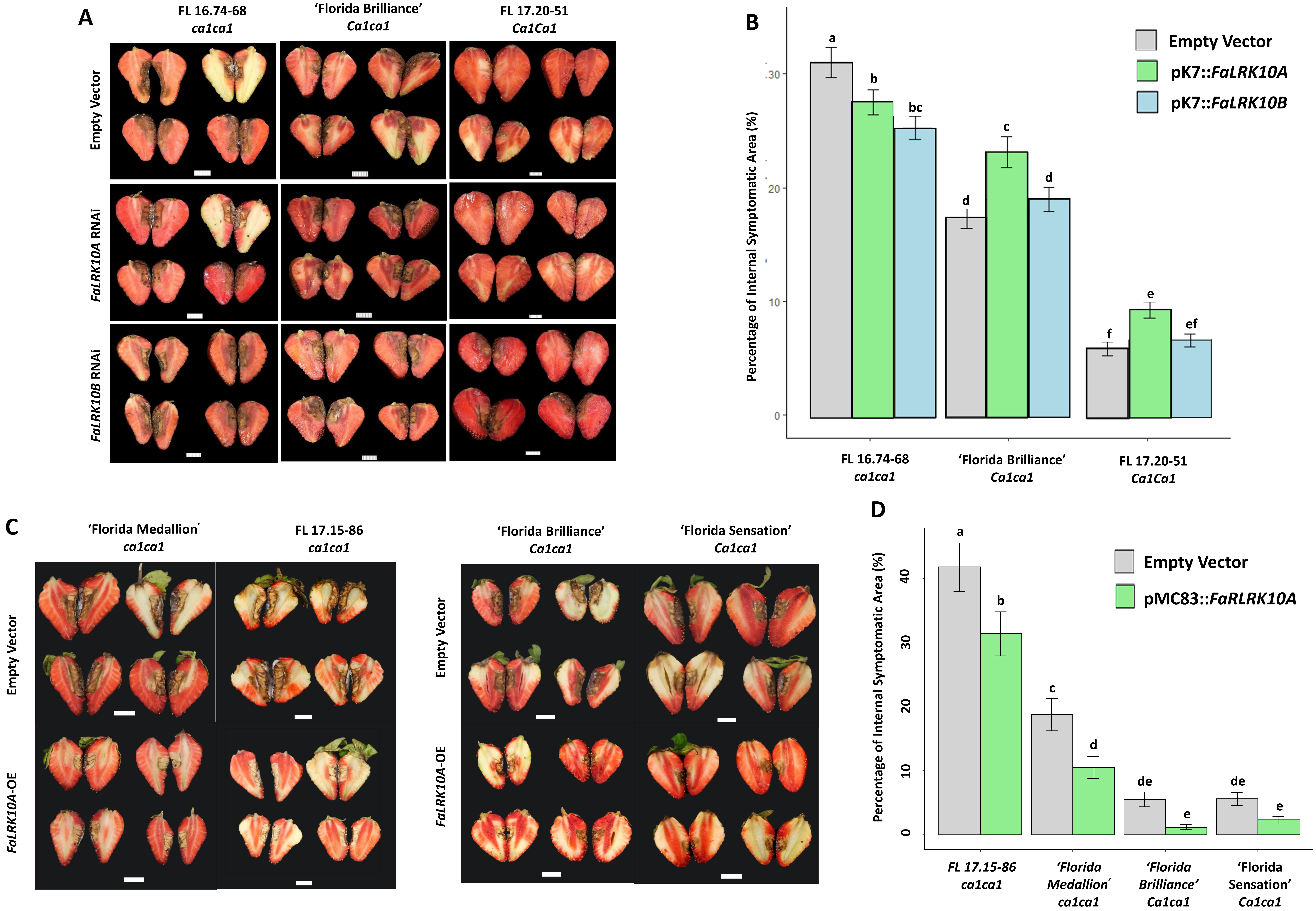

Supplementary Tables

Click here to access/download
Supplementary Material
Supplementary table_vf.docx

Click here to access/download
Supplementary Material
Figure S1_Supplementary Material.pptx

Click here to access/download
Supplementary Material
Figure S2_Supplementary Material.pptx

Figure S3_Supplementary Material

Click here to access/download

Supplementary Material

Figure S3_Supplementary Material.pptx

Click here to access/download
Supplementary Material
Figure S4_Supplementary Material.pptx

Click here to access/download
Supplementary Material
Figure S5_Supplementary Material.pptx

Click here to access/download
Supplementary Material
Figure S6_Supplementary Material.pptx

Click here to access/download
Supplementary Material
Figure S7_Supplementary Material.pptx

Click here to access/download
Supplementary Material
Figure S8_Supplementary Material.pptx

Click here to access/download
Supplementary Material
Figure S9_Supplementary Material.pptx

Click here to access/download
Supplementary Material
Figure S10_Supplementary Material.pptx

Cover letter Click here to access/download;Personal Cover;Cover letter.docx

UF UNIVERSITY of FLORIDA

Gulf Coast Research and Education Center
14625 County Road 672
Wimauma, FL 33598
(813) 634-0000
Fax (813) 634-0001
http://gcrec.ifas.ufl.edu

May 19, 2024

Dear Editor,

My co-authors and I respectfully request your consideration of the attached manuscript titled "A Telomere-To-Telomere Phased Genome of an Octoploid Strawberry Reveals a Receptor Kinase Conferring Anthracnose Resistance" for publication as an original article in GigaScience.

The cultivated strawberry possesses a highly heterogeneous and complex genome. A high-quality phased reference genome is essential for genome-enabled breeding approaches and functional genomics research in octoploid strawberries. Although the draft genome of Camarosa has significantly contributed to SNP array development, gaps, assembly errors, and missing haplotype information remain limiting factors.

In this study, we successfully generated the complete haplotype-phased genome sequence of an allo-octoploid cultivated strawberry without parental data, assembling 56 chromosomes from telomere to telomere. Leveraging highly accurate genome sequences, we identified and validated the function of the disease-resistant gene FaLRK10 against anthracnose fruit rot. Anthracnose fruit rot disease poses a growing economic threat to cultivated strawberry production worldwide. Recent studies in strawberry genomics research have facilitated the discovery of quantitative trait loci (QTL) that confer resistance to anthracnose; however, their functional genomics regions and causal variants for the resistance have not been fully characterized. The results of our study mark a significant step forward in comprehending the genes of strawberries and their roles in many important breeding characteristics in cultivated strawberry.

The authors declare no conflicting interests, and all authors have endorsed the manuscript and consented to its submission to the journal.

In order to promote research on octoploid strawberries, we have deposited our genome resources in the "Genome Database for Rosaceae" and released a pre-print on BioRxiv in 2022. Please feel free to contact us for more information.

Sincerely,

Seonghee Lee

Associate Professor
Strawberry Molecular Genetics & Genomics
Gulf Coast Research & Education Center
IFAS/University of Florida
14625 CR 672, Wimauma, FL 33598
