# Supplementary material for: A telomere-to-telomere phased genome of an octoploid strawberry reveals a receptor kinase conferring anthracnose resistance
Source: Gigascience. 2025 Mar 12;14:giaf005. doi: 10.1093/gigascience/giaf005 (PMC11899574; doi:10.1093/gigascience/giaf005)
Supplement: giaf005_Response_to_Reviewer_Comments_Original_Submission [file giaf005_response_to_reviewer_comments_original_submission.pdf]

Author's Response To Reviewer Comments

Dear Editor,

We appreciate the review and thoughtful feedback on our manuscript, “A Telomere-To-Telomere Phased Genome of an Octoploid Strawberry Reveals a Receptor Kinase Conferring Anthracnose Resistance”
We have carefully revised the manuscript in accordance with the reviewers' comments. Please find our responses below, and feel free to reach out if you have any further questions.
Best regards,
Seonghee Lee

Reviewer #1: please see attached.

Comments to the Author
This manuscript assembled a telomere-to-telomere phased genome of an Octoploid Strawberry ‘Florida Brilliance’. Moreover, the authors identified a Receptor Kinase Conferring Anthracnose Resistance. However, there are some significant problems in the current manuscript. You are welcome to submit them again after major revision.

Q1: Line 80, The studies on the T2T genome for octoploid strawberry ‘Benihoppe’ were published in Horticulture Research last year. Please cite recent literature in the part of the Background.

→ The citation was added in Line 85.

Q2: Line 138, … resistant to ARN and ARN?

→ The typo was corrected in Line 142.

Q3: Line 176, Haplotype-resolved genomes of wild octoploid strawberries have been released in 2023 (reference 28). Please cite this paper for F. chiloensis.

→ In Line 189, the citation was added.

Q4: Line 213. ‘In the combined assembly there were 652 Mb of repetitive sequence accounting for 41.66% of the genome, which was higher than other published references including ‘Royal Royce’ (38.4%), ‘Camarosa’ (36%) and ‘Wongyo 3115’ (38.75%)’. I recommend comparing the repetitive sequences of the FaFBI genome with recently assembled high-level octoploid genomes (assembled with HiFi and HiC data), such ‘Yanli’, ‘Benihoppe’ and wild octoploid strawberries.

→ In Lines 226-232, we compared the composition and proportion of repetitive sequences with FaFB1 and other genomes shown in Table S4. After downloading the genome sequences of 'Benihoppe' and 'Yanli' from Genome Database for Rosaceae (GDR), we applied EDTA to ‘Yanli’ and ‘Benihoppe’, and wild octoploid strawberry for comparison of repeat sequences under the same conditions used in this study. The differences in the proportion of repeated sequences between genomes are thought to be due to the pipeline and software used (please refer to the table below). In summary, the proportions of repetitive sequences were 43.3 (Benihoppe), 44.3 (F. chiloensis), and 42.5 (‘Yanli’). Further details are provided in Table S4.

yanli (Mao et al. 2023) Benihoppe (Song et al. 2024) F. chiloensis (Jin et al. 2023)
software RepeatScout version 1.0.5 RepeatMasker (v.4.1.2) RepeatMasker v.4.1.0
RepeatModeler version 2.0.1
PILER version 1.0
Trf version 4.10.0 [80]

LTR_FINDER version 1.0.7 [73]

Q5: Line 238, 'the number of genes in subgenomes B, C, and D were not significantly different' is not entirely accurate. We observed biased gene distribution among the sub-genomes D and A/B (D vs A, P =0.0002; D vs B, P=0.04, Table S10). Please add 'B vs D, P=0.04' in Figure 4.

→ In Lines 254-255, significant difference in the gene number between subgenome B and D were stated. The information was added in Figure 4.

Q6: Line 306-315, this paragraph is not relative to the results. You could add this part to the discussion.

→ We have combined the Results and Discussion sections in this manuscript to streamline the presentation of findings. This allows us to directly focus on the newly available high-quality reference genome for octoploid strawberry and its applications for gene identification and functional studies. By integrating these sections, we can more effectively and clearly describe the findings, making it easier for readers to follow and understand.

Q7: There are few biological validations, and the research on the LRK10 is very rough. It is suggested to add more evidence and analysis.

→ In our previous study, we identified the major resistance locus, RCa1, through multi-year field experiments with genetically diverse breeding populations. Using fine mapping and whole-genome genotyping with 50K SNP markers developed from a chromosome-scale octoploid reference genome, we narrowed the locus to about 100 kb region containing 11 genes (Salinas et al, 2019 and 2020). With the approach of pangenomic analysis, we conducted comparative genome analyses between resistant and susceptible genotypes and determined sequence variations among genes. We identified only two genes associated with disease resistance, with a sequence variation located in the promoter region of LRK10 (Fxa6Bg1786910), which results in an early stop codon, leading to premature translation termination. Thus, functional validation through Agrobacterium-mediated transient assays was performed, confirming that LRK10A is significantly associated with RCa1-mediated resistance in strawberry. This information has been included in the revised manuscript, along with Supplementary Figure 11.

Reviewer #2: Overall, this is a very clearly written and well-organized manuscript. It reports information that will be useful to the plant genomics community, and especially to those interested in polyploids and/or strawberry. In my review I have identified one substantial concern relating to the concept of phasing. From my perspective, this issue must be satisfactorily addressed as a prerequisite for publication, and this can be done relatively quickly. Otherwise, I have just a few suggestions and technical comments. These issues are addressed below.

The issue of phasing. In their analysis, the authors define two genomically phased assemblies: Haplotype-1 (H-1) and Haplotype-2 (H-2). Each assembly consists of 28 pseudochromosomes representing chromosomes 1A-1D through 7A-7D. They also make various comparisons between these two assemblies. They emphasize that this assembly phasing was accomplished without parental information, and do not characterize the two phases as parental alternatives. But if the H-1 and H-2 assemblies were not partitioned on the basis of parental origin (as they were in the 'Royal Royce' assembly) and do not constitute parental alternatives, then on what basis were they partitioned? This is a critical question for two reasons. First, if the partitioning does not reflect parental origin, then what is it based on? If it was done arbitrarily, then it is misleading to characterize the assembly as fully phased or equivalent in its phasing to a parentally phased assembly such as 'Royal Royce'. Second, if the partitioning was done arbitrarily, then it would seem to have no biological basis, in which case the several comparisons between H-1 and H-2 would have no biological meaning. Accordingly, I request that the authors provide a clear explanation of how and on what basis the H-1 and H-2 assembly phases were partitioned at the genomic level. If the partitioning was in fact arbitrary, then I request that the authors clearly acknowledge this fact, and either remove the several comparisons made between the H-1 and H-2 assemblies, or provide a sufficient justification for the

inclusion of comparisons between these biologically questionable genomic entities.

→ We appreciate the thoughtful and in-depth feedback. While it would indeed be ideal to use a tri-binning approach for genome assembly if we had both parental genomes for 'Florida Brilliance', we unfortunately only have the genome information for one parent, FL11.31-14. To address this limitation, we performed whole-genome short-read sequencing of FL11.31-14 and reassessed our genome phasing quality based on the available parental data. As shown in Supplementary Figure 2, we evaluated the phasing using haplotype-specific k-mers derived from the known parental genotype, which illustrates the collinearity between the H-1 and H-2 assemblies. In lines 168-178, we provided a detailed description of the phase blocks for Phase-1 and Phase-2, leveraging the k-mers of FL11.31-14, the female parent of 'Florida Brilliance'. Additionally, we clarified that the H-1 and H-2 assemblies are essentially arbitrary in this context. We also moved the Figure 1A to supplementary data. 1A,

Gene/protein nomenclature. Throughout the manuscript there are many inconsistencies in the nomenclatural formatting of gene and protein names, specifically in the usage of italics (+/-) and of upper versus lower case. The authors are encouraged to choose a single, widely used format, and then check all gene and protein names in the manuscript for compliance and nomenclatural consistency.

→ Throughout the manuscript, gene names have been italicized, while protein names are presented in plain text. The gene IDs were consistently matched in the manuscript, except for the first instance where the genes are referenced by their specific names. The following gene IDs were used: Fxa6B1786910 (LRK10A), Fxa6B1786910 (FaLRK10B), Fxa6Bg2100330 (LRK10a), and Fxa6Bg2100340 (LRK10b).

Genome ancestry.
Lines 60-61: the putative ancestral roles of F. viridis and F. nipponica have been questioned in subsequent studies, and so this subgenome assignment hypothesis should be stated in a more qualified way to acknowledge the existing uncertainty and disagreements.

→ In Lines 58-66, The implications of the recently proposed nomenclature were described.

Minor editorial suggestions.

Line 21: replace "ploidy" with "polyploid".

→ corrected in Line 21

Line 32: replace "susceptible" with "susceptibility".

→ corrected in Line 32

Lines 93 to 106: this section presents information without citing sources. Please provide citations.

→ In Lines 98-104, citations were provided.

Reviewer #3: I think the current MS has already met the high standard for publication. The only concern is that the authors should make the genome data, including the genome, cds, protein, gff, open to the public by submit them to public databases.

→ The genome, and annotation data bas been deposited to GDR (https://www.rosaceae.org/Analysis/14031408) and available now.
