# Supplementary material for: A telomere-to-telomere phased genome of an octoploid strawberry reveals a receptor kinase conferring anthracnose resistance
Source: Gigascience. 2025 Mar 12;14:giaf005. doi: 10.1093/gigascience/giaf005 (PMC11899574; doi:10.1093/gigascience/giaf005)
Supplement: giaf005_Response_to_Reviewer_Comments_Revision_1 [file giaf005_response_to_reviewer_comments_revision_1.pdf]

Author's Response To Reviewer Comments

Dear Editor,

We appreciate the review and thoughtful feedback on our manuscript, “A Telomere-To-Telomere Phased Genome of an Octoploid Strawberry Reveals a Receptor Kinase Conferring Anthracnose Resistance”
We have carefully revised the manuscript in accordance with the reviewers' comments. Please find our responses below, and feel free to reach out if you have any further questions.
In addition, we have also made some other small edits throughout to try to improve clarity and readability.
For any other questions, please let us know.
Best regards,
Seonghee Lee

Reviewer #2: The authors have addressed all of my minor editorial comments and most of my substantive comments. I recommend that the authors give a bit more attention to the following substantive issues, which I think can be easily done..

In the text (Introduction), the authors have addressed my concern about the subgenome identity model that proposes F. viridis and F. nipponica to be the C and D subgenome donors to the octoploid strawberry genome. However, they continue to invoke this hypothesis in the legend of Figure 4 (lines 645-646). If the authors wish to continue giving credence to that hypothesis, I recommend that they also acknowledge the many criticisms of that hypothesis and particularly the recent report strongly supportive of an alternate hypothesis (Jin et al., 2023)..

→ We fixed it in Figure 4 concerning the subgenome identity model, but we forgot to fix it in the legend. We apologize for that. The legend was corrected - F. viridis and F. nipponica to “unknown” on line 645.

On lines 172-173, the authors indicate that they had parental information only for the female parent FL 11.31-14, and not for the male parent. Upon my second review, this statement is now puzzling to me, because the identity of the male parent in the cross is reported to be FL 10-153 in the published paper (Whitaker et al., 2019, https://doi.org/10.21273/HORTSCI14327-19) reporting the release and pedigree of 'Florida Brilliance'. The respective pedigree and identity of the male parent are not acknowledged in any way in the present manuscript, which prompts me to speculate that either the FL 10-153 clone has been lost, or that perhaps the male parent was mis-identified in the variety release paper and is in fact unknown. But if the FL 10-153 clone was correctly identified as the male parent and if this clone still exists, it is surprising that the well-funded Florida program did not choose to generate sequence from it for use in trio binning in the present project. Some illumination and clarification are needed here, and the pedigree paper should be cited in some context.

Finally, if the published pedigree is correct, then I think it would be of interest to the readers of this manuscript to know the impact of shared ancestry of parents on the effort to differentiate parental assembly phases. Specifically, the published pedigree reveals that several ancestors are shared by both parents, most notably 'Winterstar' as a parent of both parents. Can the authors comment on the extent to which shared ancestry muddies the waters of phase differentiation?

→ In our preliminary experiments, we attempted a trio-binning approach using high-fidelity (HiFi) long reads from ‘Florida Brilliance’ and its parents FL 11.31-14 (female parent) and FL 10-153 (male parent) [26]. In the process we discovered that the male parent was incorrectly identified and is unknown, which did not allow a trio-binning approach. In lines 148-151, we clarified that the pedigree described in our variety release paper is not accurate. The pedigree paper was also cited in line 150.
