# Supplementary material for: A telomere-to-telomere phased genome of an octoploid strawberry reveals a receptor kinase conferring anthracnose resistance
Source: Gigascience. 2025 Mar 12;14:giaf005. doi: 10.1093/gigascience/giaf005 (PMC11899574; doi:10.1093/gigascience/giaf005)
Supplement: giaf005_Reviewer_2_Report_Original_Submission — Thomas Medford Davis, Ph.D. -- 7/1/2024 [file giaf005_reviewer_2_report_original_submission.pdf]

**Reviewer Report**

**Title: A Telomere-To-Telomere Phased Genome of an Octoploid Strawberry Reveals a Receptor Kinase Conferring Anthracnose Resistance**

**Version: Original Submission** **Date:** 7/1/2024

**Reviewer name: Thomas Medford Davis, Ph.D.**

**Reviewer Comments to Author:**

Overall, this is a very clearly written and well-organized manuscript. It reports information that will be useful to the plant genomics community, and especially to those interested in polyploids and/or strawberry. In my review I have identified one substantial concern relating to the concept of phasing. From my perspective, this issue must be satisfactorily addressed as a prerequisite for publication, and this can be done relatively quickly. Otherwise, I have just a few suggestions and technical comments. These issues are addressed below.

The issue of phasing. In their analysis, the authors define two genomically phased assemblies: Haplotype-1 (H-1) and Haplotype-2 (H-2). Each assembly consists of 28 pseudochromosomes representing chromosomes 1A-1D through 7A-7D. They also make various comparisons between these two assemblies. They emphasize that this assembly phasing was accomplished without parental information, and do not characterize the two phases as parental alternatives. But if the H-1 and H-2 assemblies were not partitioned on the basis of parental origin (as they were in the 'Royal Royce' assembly) and do not constitute parental alternatives, then on what basis were they partitioned? This is a critical question for two reasons. First, if the partitioning does not reflect parental origin, then what is it based on? If it was done arbitrarily, then it is misleading to characterize the assembly as fully phased or equivalent in its phasing to a parentally phased assembly such as 'Royal Royce'. Second, if the partitioning was done arbitrarily, then it would seem to have no biological basis, in which case the several comparisons between H-1 and H-2 would have no biological meaning. Accordingly, I request that the authors provide a clear explanation of how and on what basis the H-1 and H-2 assembly phases were partitioned at the genomic level. If the partitioning was in fact arbitrary, then I request that the authors clearly acknowledge this fact, and either remove the several comparisons made between the H-1 and H-2 assemblies, or provide a sufficient justification for the inclusion of comparisons between these biologically questionable genomic entities.

Gene/protein nomenclature. Throughout the manuscript there are many inconsistencies in the nomenclatural formatting of gene and protein names, specifically in the usage of italics (+/-) and of upper versus lower case. The authors are encouraged to choose a single, widely used format, and then check all gene and protein names in the manuscript for compliance and nomenclatural consistency.

Genome ancestry.

Lines 60-61: the putative ancestral roles of F. viridis and F. nipponica have been questioned in subsequent studies, and so this subgenome assignment hypothesis should be stated in a more qualified way to acknowledge the existing uncertainty and disagreements.

Minor editorial suggestions.

Line 21: replace "ploidy" with "polyploid".
Line 32: replace "susceptible" with "susceptibility".
Lines 93 to 106: this section presents information without citing sources. Please provide citations.

**Methods**

Are the methods appropriate to the aims of the study, are they well described, and are necessary controls included? Choose an item.

**Conclusions**

Are the conclusions adequately supported by the data shown? Choose an item.

**Reporting Standards**

Does the manuscript adhere to the journal's guidelines on [minimum standards of reporting?]() Choose an item.

Choose an item.

**Statistics**

Are you able to assess all statistics in the manuscript, including the appropriateness of statistical tests used? Choose an item.

**Quality of Written English**

Please indicate the quality of language in the manuscript: Choose an item.

**Declaration of Competing Interests**

Please complete a declaration of competing interests, considering the following questions:

- Have you in the past five years received reimbursements, fees, funding, or salary from an organisation that may in any way gain or lose financially from the publication of this manuscript, either now or in the future?
- Do you hold any stocks or shares in an organisation that may in any way gain or lose financially from the publication of this manuscript, either now or in the future?
- Do you hold or are you currently applying for any patents relating to the content of the manuscript?
- Have you received reimbursements, fees, funding, or salary from an organization that holds or has applied for patents relating to the content of the manuscript?
- Do you have any other financial competing interests?
- Do you have any non-financial competing interests in relation to this paper?

If you can answer no to all of the above, write 'I declare that I have no competing interests' below. If your reply is yes to any, please give details below.

I declare that I have no competing interests.

I agree to the open peer review policy of the journal. I understand that my name will be included on my report to the authors and, if the manuscript is accepted for publication, my named report including any attachments I upload will be posted on the website along with the authors' responses. I agree for my report to be made available under an Open Access Creative Commons CC-BY license (http://creativecommons.org/licenses/by/4.0/). I understand that any comments which I do not wish to be included in my named report can be included as confidential comments to the editors, which will not be published.

Choose an item.

To further support our reviewers, we have joined with Publons, where you can gain additional credit to further highlight your hard work (see: https://publons.com/journal/530/gigascience). On publication of this paper, your review will be automatically added to Publons, you can then choose whether or not to claim your Publons credit. I understand this statement.

Yes Choose an item.
