# Supplementary material for: A telomere-to-telomere phased genome of an octoploid strawberry reveals a receptor kinase conferring anthracnose resistance
Source: Gigascience. 2025 Mar 12;14:giaf005. doi: 10.1093/gigascience/giaf005 (PMC11899574; doi:10.1093/gigascience/giaf005)
Supplement: giaf005_Reviewer_2_Report_Revision_1 — Thomas Medford Davis, Ph.D. -- 10/22/2024 [file giaf005_reviewer_2_report_revision_1.pdf]

Reviewer Report

Title: A Telomere-To-Telomere Phased Genome of an Octoploid Strawberry Reveals a Receptor Kinase Conferring Anthracnose Resistance

Version: Revision 1 **Date:** 10/22/2024

Reviewer name: Thomas Medford Davis, Ph.D.

Reviewer Comments to Author:

The authors have addressed all of my minor editorial comments and most of my substantive comments. I recommend that the authors give a bit more attention to the following substantive issues, which I think can be easily done.

In the text (Introduction), the authors have addressed my concern about the subgenome identity model that proposes F. viridis and F. nipponica to be the C and D subgenome donors to the octoploid strawberry genome. However, they continue to invoke this hypothesis in the legend of Figure 4 (lines 645-646). If the authors wish to continue giving credence to that hypothesis, I recommend that they also acknowledge the many criticisms of that hypothesis and particularly the recent report strongly supportive of an alternate hypothesis (Jin et al., 2023).

On lines 172-173, the authors indicate that they had parental information only for the female parent FL 11.31-14, and not for the male parent. Upon my second review, this statement is now puzzling to me, because the identity of the male parent in the cross is reported to be FL 10-153 in the published paper (Whitaker et al., 2019, https://doi.org/10.21273/HORTSCI14327-19) reporting the release and pedigree of 'Florida Brilliance'. The respective pedigree and identity of the male parent are not acknowledged in any way in the present manuscript, which prompts me to speculate that either the FL 10-153 clone has been lost, or that perhaps the male parent was mis-identified in the variety release paper and is in fact unknown. But if the FL 10-153 clone was correctly identified as the male parent and if this clone still exists, it is surprising that the well-funded Florida program did not choose to generate sequence from it for use in trio binning in the present project. Some illumination and clarification are needed here, and the pedigree paper should be cited in some context.

Finally, if the published pedigree is correct, then I think it would be of interest to the readers of this manuscript to know the impact of shared ancestry of parents on the effort to differentiate parental assembly phases. Specifically, the published pedigree reveals that several ancestors are shared by both parents, most notably 'Winterstar' as a parent of both parents. Can the authors comment on the extent to which shared ancestry muddies the waters of phase differentiation?

Methods

Are the methods appropriate to the aims of the study, are they well described, and are necessary controls included? Choose an item.

Conclusions

Are the conclusions adequately supported by the data shown? Choose an item.

Reporting Standards

Does the manuscript adhere to the journal's guidelines on minimum standards of reporting? Choose an item.

Choose an item.

Statistics

Are you able to assess all statistics in the manuscript, including the appropriateness of statistical tests used? Choose an item.

Quality of Written English

Please indicate the quality of language in the manuscript: Choose an item.

Declaration of Competing Interests

Please complete a declaration of competing interests, considering the following questions:

- Have you in the past five years received reimbursements, fees, funding, or salary from an organisation that may in any way gain or lose financially from the publication of this manuscript, either now or in the future?
- Do you hold any stocks or shares in an organisation that may in any way gain or lose financially from the publication of this manuscript, either now or in the future?
- Do you hold or are you currently applying for any patents relating to the content of the manuscript?
- Have you received reimbursements, fees, funding, or salary from an organization that holds or has applied for patents relating to the content of the manuscript?
- Do you have any other financial competing interests?
- Do you have any non-financial competing interests in relation to this paper?

If you can answer no to all of the above, write 'I declare that I have no competing interests' below. If your reply is yes to any, please give details below.

I declare that I have no competing interests.

I agree to the open peer review policy of the journal. I understand that my name will be included on my report to the authors and, if the manuscript is accepted for publication, my named report including any attachments I upload will be posted on the website along with the authors' responses. I agree for my report to be made available under an Open Access Creative Commons CC-BY license (http://creativecommons.org/licenses/by/4.0/). I understand that any comments which I do not wish to

be included in my named report can be included as confidential comments to the editors, which will not be published.

Choose an item.

To further support our reviewers, we have joined with Publons, where you can gain additional credit to further highlight your hard work (see: https://publons.com/journal/530/gigascience). On publication of this paper, your review will be automatically added to Publons, you can then choose whether or not to claim your Publons credit. I understand this statement.

Yes Choose an item.
